# A forecast-to-dispatch workflow for day-ahead operation of a regional natural gas transmission network based on station-level load prediction and linepack target setting

**DOI:** 10.1371/journal.pone.0349981

**Published:** 2026-10-05

**Authors:** Ying Liu, Yong Xiao, Zhiping Tang, Simin Zhong, Chenguang Yong, Yuanyuan Tian

**Affiliations:** 1 Surface Engineering Design Center, PetroChina Southwest Oil & Gasfield Company, Chengdu, China; 2 Development and Planning Department, China National Petroleum Corporation, Beijing, China; 3 Gas Field Development and Management Department, PetroChina Southwest Oil & Gasfield Company, Chengdu, China; 4 Gas Distribution and Transmission Management Department, PetroChina Southwest Oil & Gasfield Company, Chengdu, China; 5 Kunlun Digital Technology Co., Ltd., Beijing, China; Aalto University, FINLAND

## Abstract

Day-ahead operation of regional natural gas transmission networks requires station demand forecasts and their translation into inventory-preparation references. This study evaluated a sequential forecast-to-dispatch workflow for the Chongqing regional gas network. Six forecasting models were assessed under a leakage-controlled 30-day walk-forward protocol. Across nine routine stations, mean station-level MAPE was 8.61% for SARIMAX, 8.95% for XGBoost, and 9.78% for GRU. The corresponding values were 10.93% for LSTM, 11.63% for BPNN, and 11.77% for CNN-BiLSTM-Attention. Station G was assessed separately as a data-quality boundary case; Station K was excluded because only 62 observations were available. An independent archived 18-day hourly dataset reported reconstruction MAPE of 6.7%−8.1% and peak-timing deviations of 1.0–1.5 h. A retrospective West-pipeline case for 22 April 2023 traced historical station forecasts through weighted aggregation, intermediate rounding, hourly reconstruction, and reduced-order linepack calculation. Its LFFX preparation target and the separate NGXDD and DLFX targets were 1.272, 4.702, and 1.571 × 10⁶ m³, respectively. All three targets passed case-specific deterministic inventory checks. The results support station-specific forecast-model selection and an explicit interface between daily prediction and segment-level inventory preparation.

## 1. Introduction

Natural gas infrastructure provides dispatchable flexibility for balancing variable renewable generation, seasonal heating demand, and cross-sector energy interactions in low-carbon energy systems [1]. Pipeline linepack and gas-power coordination can support renewable accommodation, demand balancing, and system resilience. Growing gas consumption has also increased the operational demands placed on regional and urban transmission networks. China's apparent natural gas consumption reached 372.6 billion m³ in 2021, a year-on-year increase of 12.7% [2,3]. Accurate day-ahead load forecasts are therefore central to secure and economical pipeline operation [4].

The Chongqing regional transmission network is a ring-shaped source-network-load-storage system with a daily peak-valley difference exceeding 10 × 10⁶ m³. Gas transport depends mainly on inlet-pressure differences and dispatch adjustments because the network has no internal compressors. Its demand combines relatively regular urban and residential loads with volatile industrial and commercial loads. Temperature, holidays, weekly cycles, and operating-regime changes jointly shape these station-level series [4–7], requiring models that represent both persistent structure and short-term disturbances.

Natural gas load forecasting has progressed from ARIMA- and SARIMA-type models for regular seasonal series to machine-learning and deep-learning methods for nonlinear, multivariate demand [8,9]. Comparative studies have evaluated LSTM, GRU, and hybrid architectures for local fluctuations and longer temporal dependencies [10–12]. Pipeline-operation research has concurrently developed optimization-based approaches for emergency scheduling, hourly day-ahead operation, and integrated gas-power systems with linepack flexibility [13–15]. Forecasting and dispatch nevertheless remain weakly connected in much of this literature: dispatch models often use assumed demand profiles, while forecasting studies commonly stop at predictive accuracy [16–20].

Closed-loop predict-and-optimize research links forecasting to the downstream decision objective. Chen et al. trained a cost-oriented renewable-generation predictor using the objective and constraints of network-constrained unit commitment [21]. Their work distinguishes predictive accuracy from operating cost. The present gas-network workflow uses sequential forecasting and inventory preparation with a linepack-deviation objective.

Related industrial-monitoring research provides additional methodological context for field deployment. Multimodal signal fusion integrates complementary operating measurements for robust diagnosis [22]. Zero-sample and zero-fault-sample methods use physical constraints, digital twins, and virtual-real signal interaction when abnormal observations are scarce [23,24]. Lightweight explainable architectures address deployment and interpretation constraints [25–27], and prior-informed neural networks incorporate engineering knowledge into reliability prediction under uncertainty [28].

Regional gas-network applications require models that accommodate operational-data heterogeneity and station-dependent predictability while producing interpretable dispatch references. Daily station forecasts must be converted into hourly withdrawal profiles before they can support segment-level inventory preparation. Field evidence remains limited on the accuracy of this conversion and the resulting linepack and pressure references.

This study evaluated a sequential forecast-to-dispatch workflow for the Chongqing regional natural gas transmission network. It connects leakage-controlled station forecasting, hourly nodal demand construction using historical intraday patterns, and reduced-order linepack and average-pressure target setting. Separate forecasting and temporal-disaggregation assessments characterize performance under heterogeneous field conditions, including a severely data-sparse station. A retrospective West-pipeline case traces historical forecasts through hourly reconstruction to an initial linepack preparation target.

## 2. Materials and methods

### 2.1 Problem definition and information flow

Day-ahead operation of the Chongqing regional natural gas transmission network requires two sequential tasks: forecasting next-day station demand from historical loads and exogenous variables, and calculating linepack targets for key segments within prescribed pressure and inventory envelopes.

Fig 1 shows the three-stage forecast-to-dispatch workflow. Layer 1 provides next-day station-level daily demand forecasts ${\hat{Q}}_{s,d+1}$, where *s* is the station index and *d* + 1 is the next day. Layer 2 converts these forecasts into hourly profiles using historical intraday allocation factors and maps the withdrawals to the modeled offtake nodes.

**Fig 1:**
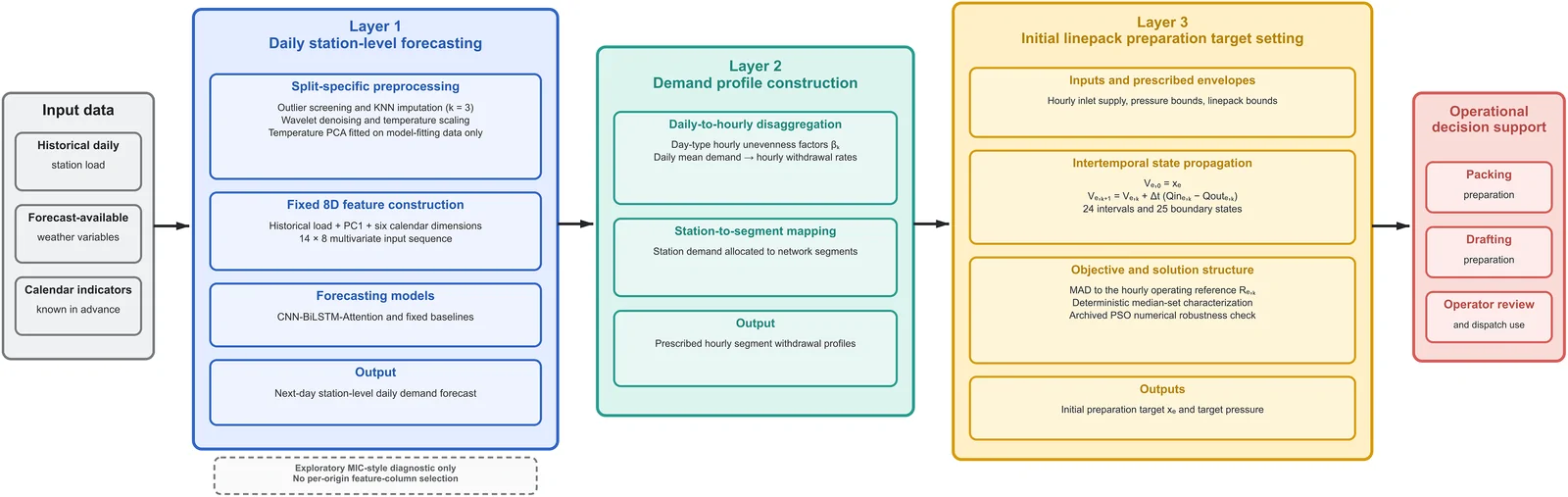
Computational information flow of the forecast-guided operational framework.

Layer 3 aggregates the hourly nodal withdrawals into segment-level demand-rate profiles. The reduced-order quasi-steady model combines these profiles with prescribed inlet supply and pressure and linepack envelopes. It returns the initial linepack preparation value *x*_e_, the corresponding average-pressure reference, and a packing or drafting recommendation for each segment.

Forecasting was assessed over a common 30-day chronological hold-out period, and temporal disaggregation was evaluated using an independent archived 18-day hourly dataset. Retrospective engineering cases traced the subsequent linepack calculations, including the West-pipeline case for 22 April 2023 in Section 3.6.

### 2.2 Study network, data sources, and preprocessing strategy

#### 2.2.1 Study network and station configuration.

Fig 2 shows the looped transmission network, its supply inlets and trunk pipelines, and the downstream station-level demand targets. The study covers transmission pipelines, excluding gathering pipelines, urban distribution pipelines, and underground storage facilities. Forecasting targets were defined at the offtake-station level to match the spatial resolution of day-ahead balancing and linepack management.

**Fig 2:**
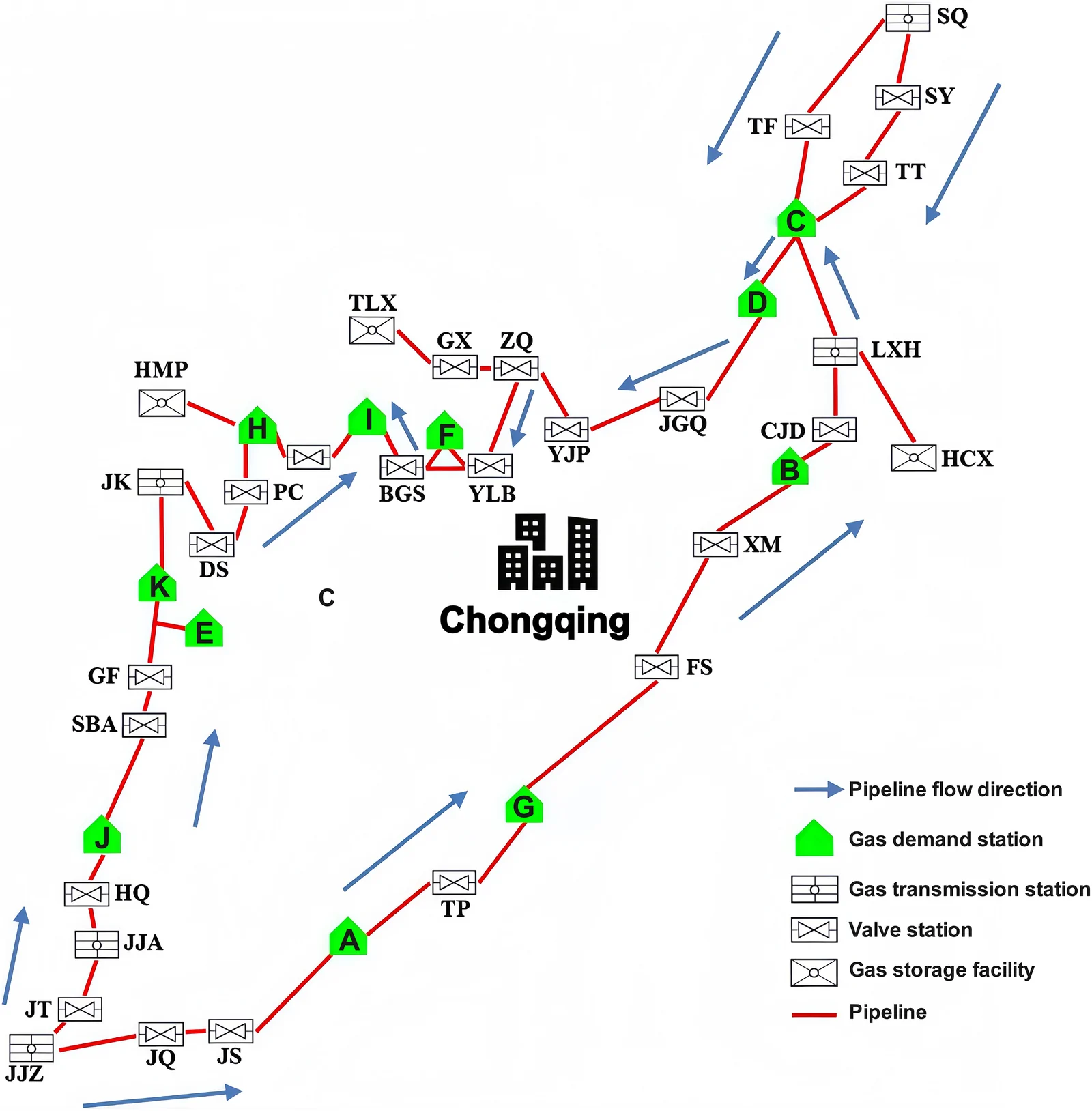
Simplified map of the Chongqing regional transmission network and station-level demand targets.

The hourly operational records span 1 November 2021–24 April 2023. Source-point measurements were grouped into 11 anonymized station targets: A (AL), B (CJX), C (DZX), D (DZ), E (GLS), F (HT), G (JJA), H (LF), I (TYW), J (XP), and K (ZL). These targets cover stable urban and residential demand, mixed industrial and commercial offtake, and intermediate regimes.

Table 1 shows received-archive source-point missingness and the separate audit of the released station-level daily series. The missingness summaries cover records received after data-owner quality control and before research-side imputation. Station missing rates are unweighted means of the constituent source-point rates. Complete-day-gap shares are missing-rate-weighted means of the source-point missing-type proportions, with total missingness as the denominator, not all calendar days.

**Table 1:** Station/source-point correspondence, telemetry completeness, and aggregate daily preprocessing audit.

Station	Source points	Received-archive missing rate (%)	Archived complete-day-gap share (%)	Daily audit outliers, *n* (%)	Daily audit *N* / imputed values / residual cells
A	ALCR, BY, JGM, NC	2.04	61.92	0 (0.00)	532 / 0 / 0
B	CLRQDS, SHRQDS	5.4	5.22	0 (0.00)	532 / 0 / 0
C	LD	1.96	0	0 (0.00)	531 / 0 / 0
D	DL, DZ	20.61	55.25	1 (0.19)	540 / 1 / 0
E	GLS	1.61	34.95	0 (0.00)	532 / 0 / 0
F	BP, CRHT, JYGSHT, JYGSKG, KYJCGL, KYLL, KYLLCNG, MLFDJ, ZRLL, ZRTYW	14.03	9.11	0 (0.00)	540 / 0 / 0
G	JJA	56.18	2.81	0 (0.00)	253 / 0 / 0
H	LF	1.94	29.15	0 (0.00)	531 / 0 / 0
I	TYW, ZRCNG, ZRDS	21.3	1.45	0 (0.00)	540 / 0 / 0
J	CQJASY, GTC, LXX, WSRQ, XNLY, XPCNG, XPJGM, XTX, XYX	10.69	6.64	0 (0.00)	540 / 0 / 0
K	ZL	0.13	0	0 (0.00)	62 / 0 / 0

In the daily-series audit, outlier percentages use the retained daily-row count N as the denominator. The final column reports N, daily-load values imputed after outlier screening, and residual missing cells across eight source/derived columns. These columns are load, three temperatures, month, weekday, weekend, and holiday. No residual missing cells remained. Record-level counters for the original hourly-to-daily preprocessing, including station-level completeness before and after processing, were not retained.

#### 2.2.2 Data sources and variable construction.

The source telemetry contained source identifiers, timestamps, and instantaneous flow-rate records. The operational SSLL records are hourly standard-volume flow rates, m³/d. For a complete day with 24 equally spaced observations, the daily mean flow rate at source point p was calculated as:


$$\begin{array}{c} {\overset{―}{Q}}_{p,d}=\frac{\text{1}}{24}\sum_{h=1}^{24}q_{p,d,h} \end{array}$$ (1)

where *q* is the hourly flow rate at source point *p* on day *d* at hour *h*, m³/d.

The corresponding daily gas volume is:


$$\begin{array}{c} D_{p,d}=\sum_{h=1}^{24}q_{p,d,h}\times \left(\frac{\text{1}}{24}\text{\textbackslash{}hspace\{0.17em}day\}\right) \end{array}$$ (2)

where Δ*t* = 1/24 day. The daily mean rate is expressed in m³/d and the daily volume in m³.

Source-point daily mean flow rates assigned to the same station were then summed to obtain its daily mean demand rate:


$$\begin{array}{c} Q_{s,d}=\sum_{p\in P_{s}}{\overset{―}{Q}}_{p,d} \end{array}$$ (3)

where *P*_s_ is the set of source points assigned to station *s*.

Candidate predictors comprised historical daily load, meteorological variables, and calendar indicators, with their engineering relevance considered during model development.

All forecasting variables were organized at daily resolution. Table 2 shows the source variables, derived quantities, and fixed feature representation used for forecasting.

**Table 2:** Source variables and final forecasting feature representation.

Source / derived variable	Description	Source variables	Transformation	Final dimension	Role in deployed model
ORGNAME	Source-point identifier	ORGNAME	None	0	Metadata only
CREATE_TIME	Timestamp of raw telemetry record	CREATE_TIME	Calendar parsing	0	Metadata only
SSLL	Hourly standard-volume flow-rate record (m³/d)	SSLL	Source-point temporal averaging before spatial aggregation	0	Not a direct model feature
*Q*_*s,d*_	Station-level daily mean demand rate after source-point time averaging and station-level spatial summation (m³/d)	Aggregated SSLL	db4 wavelet denoising	1	Historical-load feature and next-day target
Temperature component	Daily minimum, maximum, and average temperature	LT, HT, AT	Standardization and PCA; retain PC1	1	Meteorological feature
Day-of-week cycle	Day-of-week category	WK / weekday	Sine and cosine encoding	2	Calendar features
Month cycle	Month indicator	Month / month	Sine and cosine encoding	2	Calendar features
Weekend indicator	Saturday/Sunday indicator	is_weekend	Binary encoding	1	Calendar feature
Holiday indicator	Statutory-holiday indicator	HD / is_holiday	Binary encoding	1	Calendar feature
Year	Calendar year	Year	Exploratory metadata only	0	Not retained in deployed feature representation
Final feature vector	Fixed daily feature schema	Rows above with nonzero dimensions	Concatenation	8	Sequence-model input

#### 2.2.3 Preprocessing strategy and leakage control.

Fig 3 shows the preprocessing and fixed feature-construction workflow. Research-side preprocessing treated values below *Q*_1_ - 3*IQR* or above *Q*_3_ + 3*IQR* as missing. Short and partial-day hourly gaps were imputed using distance-weighted KNN with three neighbors. Hourly flow rates were temporally averaged to daily source-point means, which were then summed across source points assigned to the same station. Remaining complete-day gaps were treated at daily resolution using the same KNN configuration.

**Fig 3:**
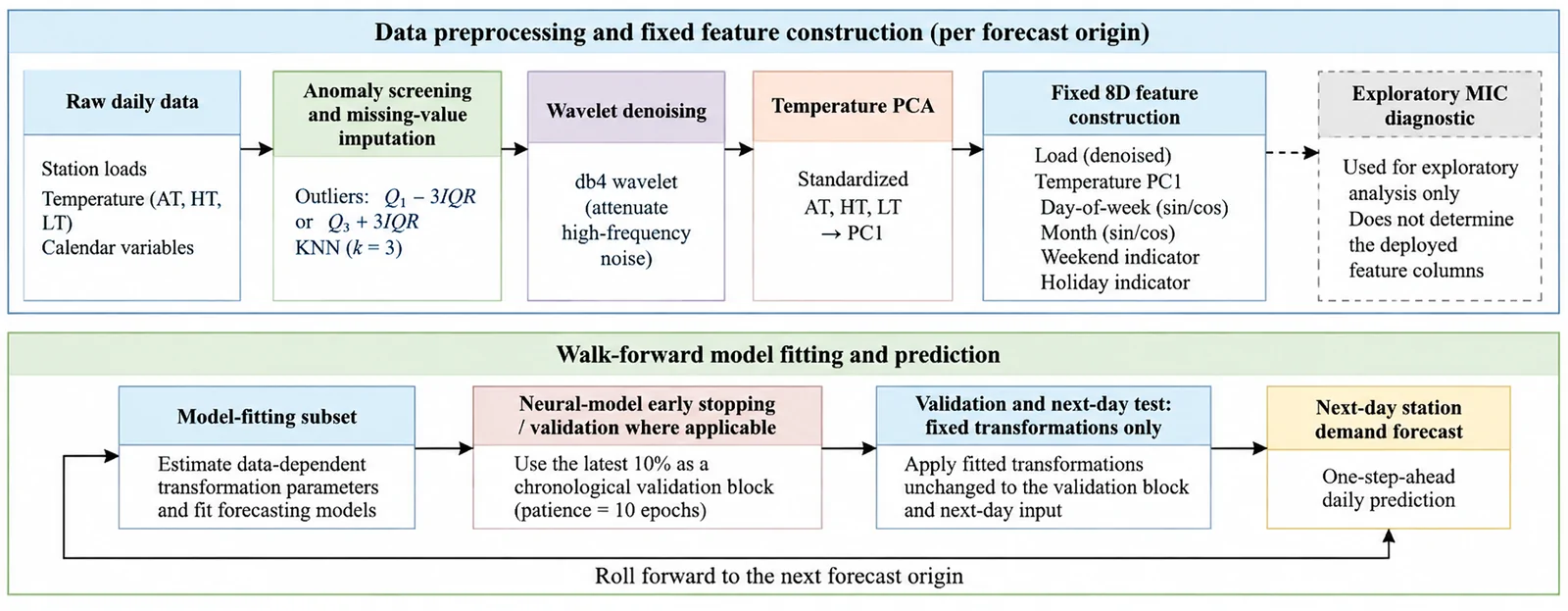
Preprocessing and fixed feature construction within walk-forward validation.

The augmented Dickey-Fuller and Ljung-Box tests characterized stationarity and autocorrelation. Daily load series were denoised with the db4 wavelet to attenuate high-frequency measurement noise. Standardized daily average (AT), maximum (HT), and minimum (LT) temperatures were compressed using principal component analysis (PCA). Temperature PC1 met the 95% cumulative explained-variance threshold in every available preprocessing record and supplied one input dimension.

At each forecast origin, outlier thresholds, the KNN imputer, wavelet thresholds, temperature scaling, PCA parameters, and feature-normalization statistics were fitted on the model-fitting subset. The fitted transformations were applied unchanged to the chronological validation block and next-day input. Validation and test observations did not contribute to transformation fitting.

### 2.3 Daily station-level forecasting model

CNN-BiLSTM-Attention is the focal hybrid architecture because it formed the original engineering forecasting workflow and supports the architectural ablation analysis. The historical hybrid forecasts were retained to reconstruct the original engineering case.

#### 2.3.1 Feature engineering and model inputs.

The forecasting pipeline used a fixed eight-dimensional feature vector at each daily time step. It comprised the wavelet-denoised station load, temperature PC1, sine and cosine encodings of day of week and month, and weekend and holiday indicators. With a 14-day lookback, CNN-BiLSTM-Attention, LSTM, GRU, and the sequence-based ablation models received a 14 × 8 multivariate sequence. BPNN and XGBoost used the flattened 112-element window. SARIMAX used normalized load history and the seven transformed exogenous dimensions obtained by excluding contemporaneous load. The feature schema remained fixed across stations and forecast origins. MIC-style dependence scores served as an exploratory development-stage diagnostic and did not determine feature columns at individual forecast origins.

For station *s*, $z_{s,d}\in {\mathbb{R}}^{m}$ is the daily feature vector with m = 8. The input uses a lookback window of L days:


$$\begin{array}{c} X_{s,d}=\left[z_{s,d-L+1},z_{s,d-L+2},\cdots ,z_{s,d}\right] \end{array}$$ (4)

The next-day prediction target is:


$$\begin{array}{c} y_{s,d}=Q_{s,d+1} \end{array}$$ (5)

where $Q_{s,d+1}$ is the observed next-day station demand rate, m³/d. The model learns the mapping:


$$\begin{array}{c} f:X_{s,d}\to Q_{s,d+1} \end{array}$$ (6)

The resulting forecast is:


$$\begin{array}{c} {\hat{Q}}_{s,d+1}=f\left(X_{s,d}\right) \end{array}$$ (7)

#### 2.3.2 CNN-BiLSTM-Attention architecture.

Fig 4 shows the forecasting architecture, which combines one-dimensional convolution, bidirectional long short-term memory (BiLSTM), and multi-head attention. The 1D-CNN extracts local patterns, including short-horizon fluctuations and abrupt changes, from the multivariate daily sequence. For filter j, the convolution is written as:

**Fig 4:**
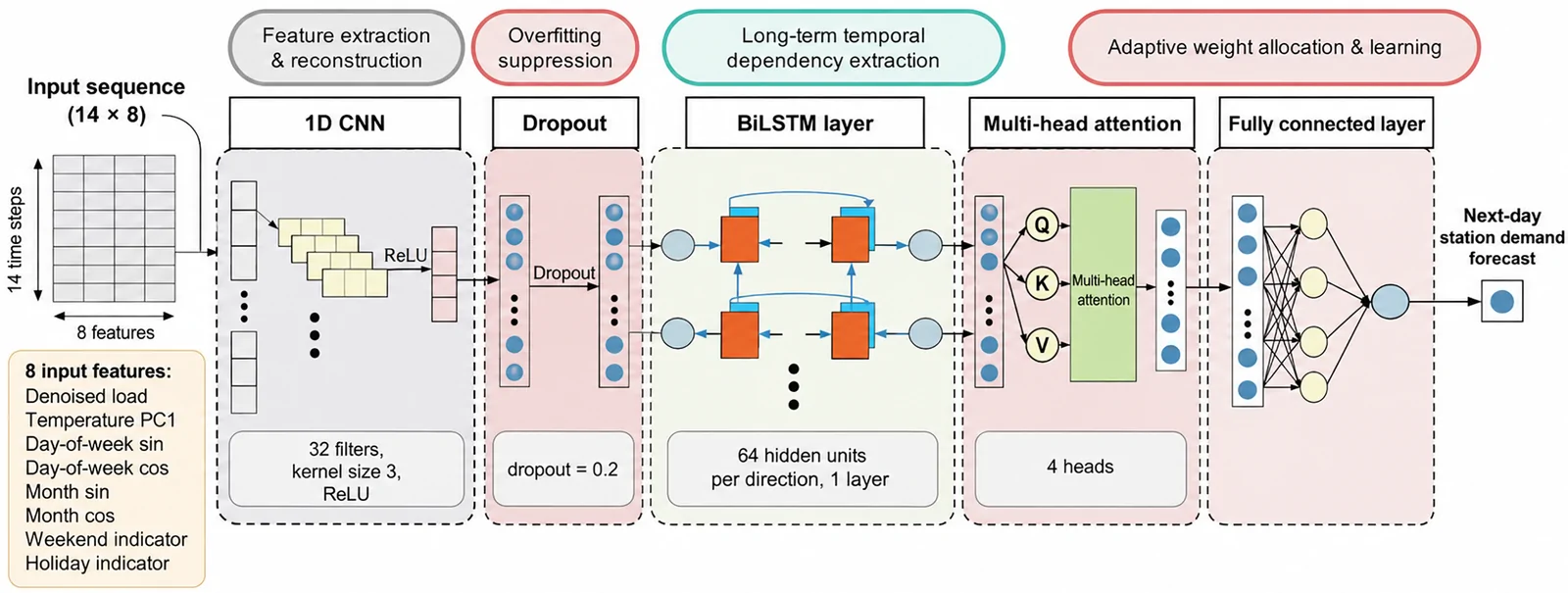
Architecture of the CNN-BiLSTM-Attention forecasting model.


$$\begin{array}{c} \text{f}j=\sigma \left(W_{j}*{𝐱}_{s,d}+b_{j}\right) \end{array}$$ (8)

where * is the convolution operator; $W_{j}$ is the filter kernel; and σ(·) is the ReLU activation function.

The extracted feature maps were then passed to the BiLSTM layer to capture bidirectional temporal dependencies:


$$\begin{array}{c} \begin{array}{c} {\vec{\text{h}}}_{d}={\text{LSTM}}_{f}\left({\text{f}}_{d},{\vec{\text{h}}}_{d-1}\right),\\ {\overset{\leftarrow }{\text{h}}}_{t}={\text{LSTM}}_{b}\left({\text{f}}_{d},{\vec{\text{h}}}_{d+1}\right),\\ {\text{h}}_{d}=\left[\vec{h_{d}}⊕\overset{\leftarrow }{h_{d}}\right] \end{array} \end{array}$$ (9)

Where f_*d*_ is the CNN feature vector at time step *d*; ${\vec{\text{h}}}_{d}$ and${\overset{\leftarrow }{\text{h}}}_{d}$ are the forward and backward hidden states. The hidden representation concatenates the forward and backward states at each sequence step.

An attention mechanism then weighted the hidden states according to their forecasting relevance. The attention module was expressed in compact scaled dot-product form:


$$\begin{array}{c} \text{Attention}\left(Q,K,V\right)=\text{softmax}\left(\frac{QK^{T}}{\sqrt{d_{k}}}\right)V \end{array}$$ (10)

where *Q*, *K*, and *V* are the query, key, and value matrices, respectively, and *d*_*k*_ is the key dimension. A fully connected output layer maps the attention context to the next-day load forecast.

#### 2.3.3 Model training settings and hyperparameters.

A compact model configuration was used for the available sample size. The 14-day lookback covers two weekly cycles and recent demand carryover. Table 3 shows the model hyperparameters.

**Table 3:** Hyperparameter settings of the CNN-BiLSTM-Attention forecasting model.

Category	Hyperparameter	Value
Input construction	Lookback window (L)	14 days
Input construction	Forecast horizon	1 day ahead
Input construction	Final input dimension	8
CNN module	Number of convolution layers	1
CNN module	Filters	32
CNN module	Kernel size	3
CNN module	Stride	1
CNN module	Padding	same
BiLSTM module	Number of BiLSTM layers	1
BiLSTM module	Hidden units per direction	64
Attention module	Number of heads	4
Attention module	Head dimension	32
Regularization	Dropout	0.2
Model complexity	Trainable parameters	116641
Validation	Chronological validation block	Most recent 10% of development window
Training	Early-stopping monitor	Validation MSE
Training	Restore best weights	Yes
Training	Batch size	32
Training	Optimizer	Adam
Training	Initial learning rate	0.001
Training	Maximum epochs	100
Training	Early stopping patience	10
Training	Loss function	MSE
Environment	Programming language	Python
Environment	Deep-learning framework	PyTorch

### 2.4 Construction of hourly nodal demand profiles from daily forecasts

#### 2.4.1 Daily-to-hourly temporal disaggregation.

Linepack analysis requires hourly withdrawal-rate profiles, whereas the forecasting module produces daily station mean demand rates [29,30]. Temporal disaggregation supplies this intermediate operational input.

The predicted daily mean demand rate of each station is converted to an hourly flow-rate profile using day-type-specific historical unevenness factors. For station s, forecast day *d* + 1, hour *k*, and day type *c*, the reconstruction is defined as:


$$\begin{array}{c} {\hat{q}}_{s,d+1,k}={\hat{Q}}_{s,d+1}\beta_{s,k,c} \end{array}$$ (11)

subject to


$$\begin{array}{c} \frac{\text{1}}{24}\sum_{k=1}^{24}\beta_{s,k,c}=1 \end{array}$$ (12)

where ${\hat{q}}_{s,d+1,k}$ is the reconstructed station hourly flow rate, m³/d; ${\hat{Q}}_{s,d+1}$ is the predicted daily mean demand rate, m³/d; and $\beta_{s,k,c}$ is the dimensionless hourly unevenness factor for station *s*, hour *k*, and day type *c*. The volume assigned to interval *k* is:


$$\begin{array}{c} \Delta V_{s,d+1,k}={\hat{q}}_{s,d+1,k}\times \left(\frac{\text{1}}{24}\text{\textbackslash{}hspace\{0.17em}day\}\right) \end{array}$$ (13)

where Δt = 1/24 day, and $\Delta V_{s,d+1,k}$ is the gas volume assigned to interval *k*, m³.

Because the 24 hourly unevenness factors have a mean value of 1:


$$\begin{array}{c} \frac{\text{1}}{24}\sum_{k=1}^{24}{\hat{q}}_{s,d+1,k}={\hat{Q}}_{s,d+1} \end{array}$$ (14)

Temporal disaggregation therefore preserves the predicted daily mean demand rate. Equivalently, the sum of the 24 hourly interval volumes equals the predicted daily gas volume.

Hourly unevenness factors were estimated from the preceding training period and fixed for validation. Tables 4 and 5 show the hourly withdrawal schedules for the West and East pipeline cases. The displayed unevenness factors are rounded to two decimal places; calculations use the full-precision factors.

**Table 4:** Reconstructed hourly offtake schedule for the West pipeline case.

Hour	Hourly unevenness factor, *β_k_*	Offtake rate (10^4^ m³/d)	Hour	Hourly unevenness factor, *β_k_*	Offtake rate (10^4^ m³/d)
00:00	0.88	424.98	12:00	1.09	525.73
01:00	0.89	430.79	13:00	0.98	472.94
02:00	0.92	440.88	14:00	0.97	468.02
03:00	0.94	451.13	15:00	0.98	470.41
04:00	1.05	507.58	16:00	0.98	472.75
05:00	1.16	560.57	17:00	0.98	470.88
06:00	1.16	557.87	18:00	0.97	468.19
07:00	1.15	553.87	19:00	0.97	465.16
08:00	1.14	548.50	20:00	0.87	417.17
09:00	1.12	540.75	21:00	0.87	417.76
10:00	1.11	534.76	22:00	0.86	415.90
11:00	1.10	531.40	23:00	0.86	414.49

**Table 5:** Reconstructed hourly offtake schedule for the East pipeline case.

Hour	Hourly unevenness factor, *β_k_*	Offtake rate(10^4^ m³/d)	Hour	Hourly unevenness factor, *β_k_*	Offtake rate (10^4^ m³/d)
00:00	0.88	846.84	12:00	1.09	1047.59
01:00	0.89	858.41	13:00	1.05	1011.43
02:00	0.92	878.53	14:00	1.16	1117.03
03:00	0.94	898.95	15:00	1.16	1111.64
04:00	0.98	942.41	16:00	0.98	942.02
05:00	0.97	932.59	17:00	0.98	938.30
06:00	0.98	937.36	18:00	0.97	932.94
07:00	1.15	1103.66	19:00	0.97	926.90
08:00	1.14	1092.97	20:00	0.87	831.28
09:00	1.12	1077.53	21:00	0.87	832.45
10:00	1.11	1065.60	22:00	0.86	828.74
11:00	1.10	1058.90	23:00	0.86	825.93

Operational use requires periodic updating of the hourly unevenness factors. Under routine conditions, the factors can be recalibrated from a rolling window of recent comparable days grouped by season and day type. Holidays, abrupt industrial-load changes, abnormal weather, and emergency conditions require updates from the latest hourly telemetry and dispatch-operator review before target setting.

#### 2.4.2 Station-to-node demand mapping.

Hourly station demand rates were mapped to network nodes using the station-offtake correspondence of the Chongqing regional network model. Each nodal input is a withdrawal rate at node n and hour k, m³/d. For a station corresponding directly to a modeled offtake node, the mapping is one-to-one:


$$\begin{array}{c} {\hat{q}}_{n,k}={\hat{q}}_{s,k} \end{array}$$ (15)

If the demand rate represented by station s is distributed over multiple nodes, the station-level hourly rate can be allocated by dimensionless nodal participation factors γ:


$$\begin{array}{c} {\hat{q}}_{n,k}=\gamma_{n|s}{\hat{q}}_{s,k},\text{\textbackslash{}hspace\{1em}\;\}\sum_{n\in N\left(s\right)}\gamma_{n|s}=1 \end{array}$$ (16)

where γ is the dimensionless nodal participation factor and *N*(*s*) is the set of nodes associated with station *s*.

The mapped hourly withdrawal rates were grouped by representative pipeline segment to form the segment-level demand-rate profiles used by the reduced-order target-setting model.

#### 2.4.3 Validation of the disaggregation rule.

Reconstruction-based validation assessed the temporal-disaggregation rule on historical days with complete hourly observations. Hourly profiles were reconstructed from the observed daily mean flow rate using unevenness factors estimated from prior data:


$$\begin{array}{c} {\tilde{q}}_{s,d,k}=Q_{s,d}^{\text{obs}}\beta_{s,k,c} \end{array}$$ (17)

where $Q_{s,d}^{\text{obs}}$ is the observed daily mean demand rate, m³/d, and ${\tilde{q}}_{s,d,k}$ is the reconstructed hourly flow rate, m³/d.

The reconstructed and observed hourly series are flow rates expressed in m³/d. Hourly MAPE and peak-timing error describe profile agreement; daily-volume conservation is checked separately.

The first indicator was hourly MAPE:


$$\begin{array}{c} {\text{MAPE}}_{hour}=\frac{\text{1}}{24}\sum_{k=1}^{24}\left|\frac{q_{s,k}^{obs}-{\tilde{q}}_{s,k}}{q_{s,k}^{obs}}\right|\times 100\% \end{array}$$ (18)

A daily-volume conservation diagnostic is defined as:


$$\begin{array}{c} E_{\text{cum}}=\frac{\left|\sum_{k=1}^{24}{\tilde{q}}_{s,k}\Delta t-\sum_{k=1}^{24}q_{s,k}^{\text{obs}}\Delta t\right|}{\sum_{k=1}^{24}q_{s,k}^{\text{obs}}\Delta t}\times 100\% \end{array}$$ (19)

With the observed daily-mean baseline in Eq. (17), the mean-one condition in Eq. (12), and Δt = 1/24 day, the reconstructed daily volume equals the observed daily volume apart from numerical rounding.

The third indicator was the peak-timing error:


$$\begin{array}{c} {\text{E}}_{peak}=\left|k_{peak}^{obs}-k_{peak}^{rec}\right| \end{array}$$ (20)

where $k_{peak}^{obs}$ and $k_{peak}^{rec}$ are the observed and reconstructed peak-demand hours, respectively.

Temporal disaggregation was assessed using archived 18-day summaries for LFFX, NGXDD, and DLFX. Complete date-level reconstruction inputs and metric calculations needed to rederive every summary entry are unavailable, as are balanced seasonal and abnormal-day statistics.

### 2.5 Linepack-oriented operational optimization model

The linepack target-setting model uses prescribed inlet supply and hourly segment withdrawals to calculate preparation references within pressure and inventory envelopes.

#### 2.5.1 Key pipeline segments and operating zones.

The model represents LFFX as a low-pressure western buffering corridor and NGXDD and DLFX as higher-pressure eastern transmission corridors with larger static inventories. This partition captures the segments where linepack flexibility is concentrated at the resolution of the historical engineering model.

For each segment *e*, the admissible linepack interval follows from prescribed pressure limits and throughput conditions. An interior allowable band applies conservative engineering margins to the minimum and maximum admissible linepack, $V_{e}^{min}$ and $V_{e}^{max}$, respectively. Its lower and upper bounds are:


$$\begin{array}{c} V_{e}^{L}=\left(1+\beta_{L}\right)V_{e}^{min} \end{array}$$ (21)

$$\begin{array}{c} V_{e}^{U}=\left(1-\beta_{U}\right)V_{e}^{max} \end{array}$$ (22)

where $\beta_{L}$ and $\beta_{U}$ are the dimensionless safety-margin factors, $\beta_{L}=0.1$ and $\beta_{U}=0.2$. The initial preparation value x_e_ is selected within $\left[V_{e}^{L},V_{e}^{U}\right]$.

#### 2.5.2 Reduced-order quasi-steady target-setting formulation.

The target-setting model represents each segment by a lumped linepack state at hourly resolution. For segment *e*, *x*_e_ is the initial preparation inventory for the next-day horizon, m³, with *V_e,0_*(*x*_e_) = *x*_e_. Candidate values generate inventory trajectories under prescribed inlet-supply and withdrawal-rate profiles.

Comparing x_e_ with the currently observed linepack determines the preparation direction before the next-day horizon. A higher preparation value indicates packing, a lower value indicates drafting, and a negligible difference indicates no substantial adjustment.

The horizon contains *N_h_* = 24 hourly intervals and 25 boundary states. *V_e,k_* is the segment linepack at boundary *k* = 0,...,*N_h_*, expressed as standard-condition gas volume, m³.

Eq. (23) relates this inventory to segment-average pressure:


$$\begin{array}{c} V_{e,k}=A_{e}L_{e}\frac{P_{e,avg,k}}{P_{std}}\frac{T_{std}}{T_{e}}\frac{\text{1}}{Z_{e,k}} \end{array}$$ (23)

where *A*_*e*_ is the segment cross-sectional area, m²; *L*_*e*_ is the segment l*e*ngth, m; $P_{e,avg,k}$ is the average pressure of segment *e* at hour *k*, MPa; $Z_{e,k}$ is the dimensionless compressibility factor; *T*_*e*_ is the gas temperature, K; *P*_*std*_ i_*s*_ the standard reference pressure, MPa; and *T*_*std*_ is the standard reference temperature, K.

The hourly operating reference is the midpoint of the prescribed throughput-dependent safe-linepack envelope:


$$\begin{array}{c} R_{e,k}=\frac{V_{e,k}^{min}+V_{e,k}^{max}}{\text{2}} \end{array}$$ (24)

where *V_e,k^min^_* and *V_e,k^max^_* are the lower and upper safe-linepack limits at interval *k*, m³. *R*_*e,k*_ is their midpoint, m³.

The initial preparation value minimizes the mean absolute deviation of its linepack trajectory from the hourly operating reference:


$$\begin{array}{c} {min}_{x_{e}}J_{e}\left(x_{e}\right)=\frac{\text{1}}{N_{h}}\sum_{k=0}^{N_{h}-1}\left|V_{e,k}\left(x_{e}\right)-R_{e,k}\right| \end{array}$$ (25)

The objective averages the absolute deviations of the 24 interval-start states *V_e,0_*(*x*_e_),...,*V_e,23_*(*x*_e_) from their hourly references. The terminal state *V_e,24_*(*x*_e_) is included in the end-of-day conservation and envelope-feasibility checks, but not in the 24-term objective.

The preparation value is restricted to the interior allowable band. Every candidate-generated state is checked against the segment-level linepack and associated average-pressure envelopes.

The segment-average pressure associated with each candidate-generated linepack state must remain within the prescribed throughput-dependent pressure envelope:


$$\begin{array}{c} P_{e,k}^{min}\leq P_{e,\text{avg},k}\left(x_{e}\right)\leq P_{e,k}^{max},\text{\textbackslash{}hspace\{1em}\;\}k=0,\ldots ,N_{h} \end{array}$$ (26)

where $P_{e,k}^{min}$ and $P_{e,k}^{max}$ are the lower and upper pressure limits at interval *k*, MPa.

The complete candidate-generated linepack trajectory, including the end-of-day state, must remain within the throughput-dependent linepack envelope, while *x*_e_ must lie in the allowable preparation interval:


$$\begin{array}{c} \begin{array}{c} V_{e,k}^{min}\leq V_{e,k}\left(x_{e}\right)\leq V_{e,k}^{max},\text{\textbackslash{}hspace\{1em}\;\}k=0,\ldots ,N_{h}\\ x_{e}\in \left[V_{e}^{L},V_{e}^{U}\right] \end{array} \end{array}$$ (27)

The segment inventory state is propagated between consecutive hourly boundaries using the prescribed aggregate inlet-supply and withdrawal rates:


$$\begin{array}{c} \begin{array}{c} V_{e,0}\left(x_{e}\right)=x_{e}\\ V_{e,k+1}\left(x_{e}\right)=V_{e,k}\left(x_{e}\right)+\Delta t\left(Q_{e,k}^{\text{in}}-Q_{e,k}^{\text{out}}\right),\text{\textbackslash{}hspace\{1em}\;\}k=0,\ldots ,N_{h}-1 \end{array} \end{array}$$ (28)

where *Q_e,k^in^_* and *Q_e,k^out^_* are the prescribed inlet-supply and aggregate withdrawal rates, standard m³/d; and Δt = 1/24 day is the time step.

For fixed inlet-supply and withdrawal-rate profiles, define the cumulative net inflow before boundary *k* as:


$$\begin{array}{c} C_{e,k}=\sum_{j=0}^{k-1}\Delta t\left(Q_{e,j}^{\text{in}}-Q_{e,j}^{\text{out}}\right),\text{\textbackslash{}hspace\{1em}\;\}C_{e,0}=0 \end{array}$$ (29)

where $C_{e,k}$ is the cumulative net inventory change before boundary *k*, m³.

Accordingly, changing x_e_ shifts the complete candidate trajectory while preserving its hour-to-hour increments:


$$\begin{array}{c} V_{e,k}\left(x_{e}\right)=x_{e}+C_{e,k},\text{\textbackslash{}hspace\{1em}\;\}k=0,\ldots ,N_{h} \end{array}$$ (30)

Inlet supply is prescribed, and segment withdrawals follow the temporal-disaggregation and station-to-node mapping procedures in Section 2.4. The engineering cases use their historical hourly withdrawal inputs, with network topology, valve and compressor status, and nominal flow direction fixed.

#### 2.5.3 Deterministic characterization and PSO robustness analysis.

With prescribed inlet-supply and withdrawal profiles, the one-dimensional L1 objective has a deterministic median-set solution.

Substitution of the cumulative form into Eq. (25) gives:


$$\begin{array}{c} J_{e}\left(x_{e}\right)=\frac{\text{1}}{N_{h}}\sum_{k=0}^{N_{h}-1}\left|x_{e}-\left(R_{e,k}-C_{e,k}\right)\right| \end{array}$$ (31)

Let *r_e,k_* = *R_e,k_* – *C_e,k_*, with the 24 values ordered from *r_e,_*_(1)_ to *r_e,_*_(24)_. The unconstrained minimizing set is:


$$\begin{array}{c} \underset{x_{e}}{argmin}\text{\textbackslash{}hspace\{0.17em}\}J_{e}\left(x_{e}\right)=\left[r_{e,\left(12\right)},r_{e,\left(13\right)}\right] \end{array}$$ (32)

If the median set intersects the feasible candidate interval implied by Eqs. (26) and (27), that intersection is optimal. Otherwise, the nearest feasible boundary minimizes the objective. PSO was the historical numerical implementation and was compared with this deterministic reference.

For each historical case, the archived PSO-selected preparation value is reported as the operating target after confirming inventory feasibility and optimal-set membership. When several minimizers exist, the archived selected member fixes the reported operating value.

Each particle encodes a candidate preparation value *x_i^(g)^_*, m^3^, for segment *e* at iteration *g*. Its state is updated as:


$$\begin{array}{c} v_{i}^{\left(g+1\right)}=\omega v_{i}^{\left(g\right)}+c_{\text{1}}r_{\text{1}}\left(p_{best}^{\left(g\right)}-x_{i}^{\left(g\right)}\right)+c_{\text{2}}r_{\text{2}}\left(g_{best}^{\left(g\right)}-x_{i}^{\left(g\right)}\right) \end{array}$$ (33)

$$\begin{array}{c} x_{i}^{\left(g+1\right)}=x_{i}^{\left(g\right)}+v_{i}^{\left(g+1\right)} \end{array}$$ (34)

where $v_{i}^{\left(g\right)}$ is the search velocity of particle *i* at iteration *g*; ω is the inertia weight; *c*_1_ and *c*_2_ are the co*g*nitive and social acceleration coefficients; $r_{\text{1}}$ and $r_{\text{2}}$ are independent uniform random numbers in [0,1]; and $p_{best}^{\left(g\right)}$ and $g_{best}^{\left(g\right)}$ are the personal best position of particle i and the global best swarm position, respectively.

To ensure feasibility, the updated position is projected back to the allowable interval if necessary:


$$\begin{array}{c} x_{i}^{\left(g+1\right)}=min\left[max\left(x_{i}^{\left(g+1\right)},V_{e}^{L}\right),V_{e}^{U}\right] \end{array}$$ (35)

The search stops at the maximum iteration count or when improvement in the best-so-far objective becomes negligible. Table 6 shows the PSO settings. Convergence is evaluated using the best-so-far objective, separately from instantaneous particle fitness.

**Table 6:** PSO settings for initial linepack preparation.

Item	Symbol	Value	Unit / note
Swarm size	*N*_*sw*_	20	particles
Max iterations	*G*_*max*_	10	iterations
Inertia weight	ω	0.7	dimensionless
Acceleration coefficients	*c*_1_, *c*_2_	1.4, 1.4	dimensionless
Random factors	*r*_1_, *r*_2_	*U*(0,1)	independently sampled
Decision variable	x_e_	initial linepack preparation value for segment e	m^3^
Feasible interval	[*V*_*e*_^*L*^,*V*_*e*_^*U*^]	LFFX: [1181200, 1311200]; NGXDD: [3803000, 5703000]; DLFX: [1389300, 1729300]	m^3^, case-specific PSO search bounds
Boundary handling	/	projection	Eq. (35)

Optimizer robustness was assessed with 30 independent seeds at the reference setting of 20 particles and 10 iterations. Computational-budget sensitivity used 40 particles for 10 iterations and 20 particles for 30 iterations. Each run recorded the final best objective and solver-returned preparation value. The released diagnostics interpolate retained objective-support values and do not reconstruct the full inventory model from original inputs. Deterministic grids use increments of 0.010, 0.050, and 0.020 × 10⁶ m³ for LFFX, NGXDD, and DLFX, respectively. S6 Table shows the case-specific engineering inputs and continuous median-set and state-envelope checks.

### 2.6 Evaluation protocol and baseline models

#### 2.6.1 Forecasting evaluation metrics and walk-forward validation.

Forecast accuracy was evaluated with MAPE, MedianAPE, MAE, and RMSE. MAPE supports scale-independent comparison across stations and with previous gas-demand studies. MedianAPE describes typical relative error when low loads or isolated large errors skew MAPE. MAE reports deviation in demand units, and RMSE assigns greater weight to large errors relevant to dispatch preparation.


$$\begin{array}{c} \text{MAPE}=\frac{\text{1}}{n}\sum_{i=1}^{n}\left|\frac{{\hat{y}}_{i}-y_{i}}{y_{i}}\right|\times 100\% \end{array}$$ (36)

$$\begin{array}{c} \text{MedianAPE}=\text{median}\left(\frac{\left|{\hat{y}}_{\text{1}}-y_{\text{1}}\right|}{\left|y_{\text{1}}\right|},\cdots ,\frac{\left|{\hat{y}}_{n}-y_{n}\right|}{\left|y_{n}\right|}\right) \end{array}$$ (37)

$$\begin{array}{c} \text{MAE}=\frac{\text{1}}{n}\sum_{i=1}^{n}\left|{\hat{y}}_{i}-y_{i}\right| \end{array}$$ (38)

$$\begin{array}{c} \text{RMSE}=\sqrt{\frac{\text{1}}{n}{\sum_{i=1}^{n}\left({\hat{y}}_{i}-y_{i}\right)}^{\text{2}}} \end{array}$$ (39)

where $y_{i}$ and ${\hat{y}}_{i}$ are the observed and predicted daily demand values for sample *i*, respectively, and *n* is the number of evaluated days.

Stations A-F and H-J contained 531–540 consecutive daily observations; Station G contained 253 observations and was analyzed as a data-quality boundary case. The final 30 observations of each evaluable station formed the chronological hold-out period, with a 14-day lookback for one-day-ahead prediction. Station K contained 62 observations and was excluded from the common comparison because it could not support the shared lookback, validation, and hold-out design.

At forecast origin t, the historical development window contained observations available at or before t. Its latest 10% formed the chronological validation block; earlier observations formed the model-fitting subset. Neural models were fitted on the model-fitting subset and used the validation block for early stopping. Baseline hyperparameters were fixed before hold-out evaluation. SARIMAX was refitted to all available transformed history at each origin, whereas XGBoost was fitted on the transformed model-fitting subset.

After the observation at *t* + 1 became available, the origin advanced by one day and the historical window expanded. This process covered all 30 hold-out days, with identical chronological origins and leakage-control procedures across forecasting models.

#### 2.6.2 Baseline and ablation design.

The comparison evaluated accuracy, stability, and deployment suitability under a common operational forecasting protocol. The benchmark set comprised statistical, tree-based, neural-network, and ablation models.

SARIMAX represented linear seasonal forecasting with exogenous meteorological and calendar inputs. Next-day calendar indicators were treated as known in advance, whereas the three temperature covariates used a one-day persistence forecast from the latest available observation. Observed test-day weather was not used.

XGBoost represented nonlinear tabular regression using the flattened 14 × 8 feature window.

BPNN provided a feed-forward neural reference using the flattened 112-element window; LSTM and GRU retained the 14 × 8 sequence representation.

The ablation variants CNN-BiLSTM, BiLSTM-Attention, and CNN-Attention isolated local feature extraction, bidirectional temporal modeling, and temporal weighting. Baseline and ablation models used the same preprocessing, walk-forward origins, and metrics.

For prospective deployment, candidate models should be compared by chronological validation within each station. The selected model would then generate that station's day-ahead forecast.

#### 2.6.3 Data splitting, implementation settings, and reproducibility.

Table 7 shows the fixed baseline-model input representations and configurations used in hold-out evaluation.

**Table 7:** Fixed baseline-model input representations and configurations for hold-out evaluation.

Model	Input	Fixed configuration	Validation use
SARIMAX	Normalized load history and seven transformed exogenous dimensions; next-day temperature by one-day persistence and calendar indicators known in advance	order=(1,0,1); seasonal order=(1,0,0,7)	No validation tuning; refitted at each origin
XGBoost	Flattened 14 × 8 window (112 elements)	300 estimators; depth = 3; learning rate = 0.05; subsample = 0.8; colsample = 0.8	No validation tuning; fitted on model-fitting subset
BPNN	Flattened 14 × 8 window (112 elements)	Hidden layers=(64,32); dropout = 0.2; Adam; learning rate = 0.001	Chronological validation for early stopping
LSTM	14 × 8 multivariate sequence	64 hidden units; 1 layer; dropout = 0.2; Adam; learning rate = 0.001	Chronological validation for early stopping
GRU	14 × 8 multivariate sequence	64 hidden units; 1 layer; dropout = 0.2; Adam; learning rate = 0.001	Chronological validation for early stopping

Uncertainty in model-level MAPE was quantified with a paired station bootstrap across the nine routine evaluable stations. Each of 10,000 fixed-seed resamples drew stations with replacement while retaining paired model results. Station G was excluded because it was pre-specified as a data-quality boundary case.

S1 Code contains forecasting scripts, fixed configurations, recorded outputs, illustrative hourly reconstruction, and support-based PSO diagnostics. It also contains the numerical inputs and script for the retrospective West-pipeline calculation. S1 Data contains the indexed daily series and forecasting results.

## 3. Results and discussion

### 3.1 Demand characteristics and forecasting context

#### 3.1.1 Station-level heterogeneity, periodicity, and operational context.

The 11 station targets represent distinct demand regimes. Stations A, B, I, and J show comparatively regular urban or composite demand. Stations F and H contain stronger industrial-commercial variation and more frequent regime changes. Stations C, D, E, and K occupy intermediate regimes, while Station G is a data-sparse boundary case.

Source-point composition adds another dimension of heterogeneity. Station F aggregates 10 source points and Station J nine; Stations C, E, G, H, and K each contain one source point. Station-level forecasting preserves these spatial differences for the subsequent dispatch analysis.

Fig 5 shows the contrasting temporal patterns of Stations A and F. Station A had smoother annual and monthly profiles and a recurring weekly cycle, including lower Sunday demand. Station F showed more frequent short-term oscillations, abrupt local extrema, and stronger within-month variation. Annual, monthly, and weekly patterns therefore varied in strength across stations.

**Fig 5:**
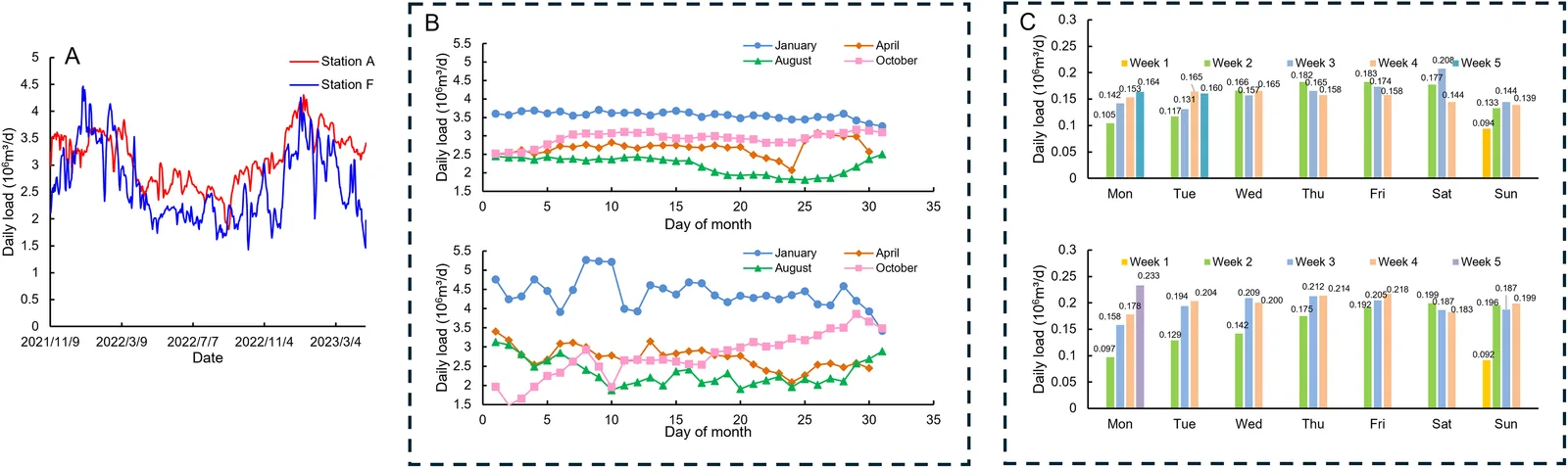
Multi-timescale variation patterns of daily load at Stations A and F. A) Annual daily load profiles of Stations A and F. B) Monthly daily load profiles of representative months in each season (January for winter, April for spring, August for summer, and October for autumn) for Stations A and F. C) Weekly daily load profiles in April and October at Station A.

#### 3.1.2 Temperature and calendar effects on daily load.

Fig 6 shows the temperature dependence of Station F's mixed load. Mean daily demand decreased from 5.04 × 10⁶ m³/d at 0–5 °C to 2.47 × 10⁶ m³/d at 25–30 °C. It reached 2.20 × 10⁶ m³/d at 35–40 °C, following a small rebound at 30–35 °C. Figs 7 and 8 show the variation in load across holidays. New Year's Day differed little from the non-holiday reference, whereas Qingming Festival, Labor Day, Dragon Boat Festival, National Day, and Spring Festival had lower demand. National Day and Spring Festival had the largest reductions. These nonlinear temperature and calendar effects support their inclusion in the candidate feature set.

**Fig 6:**
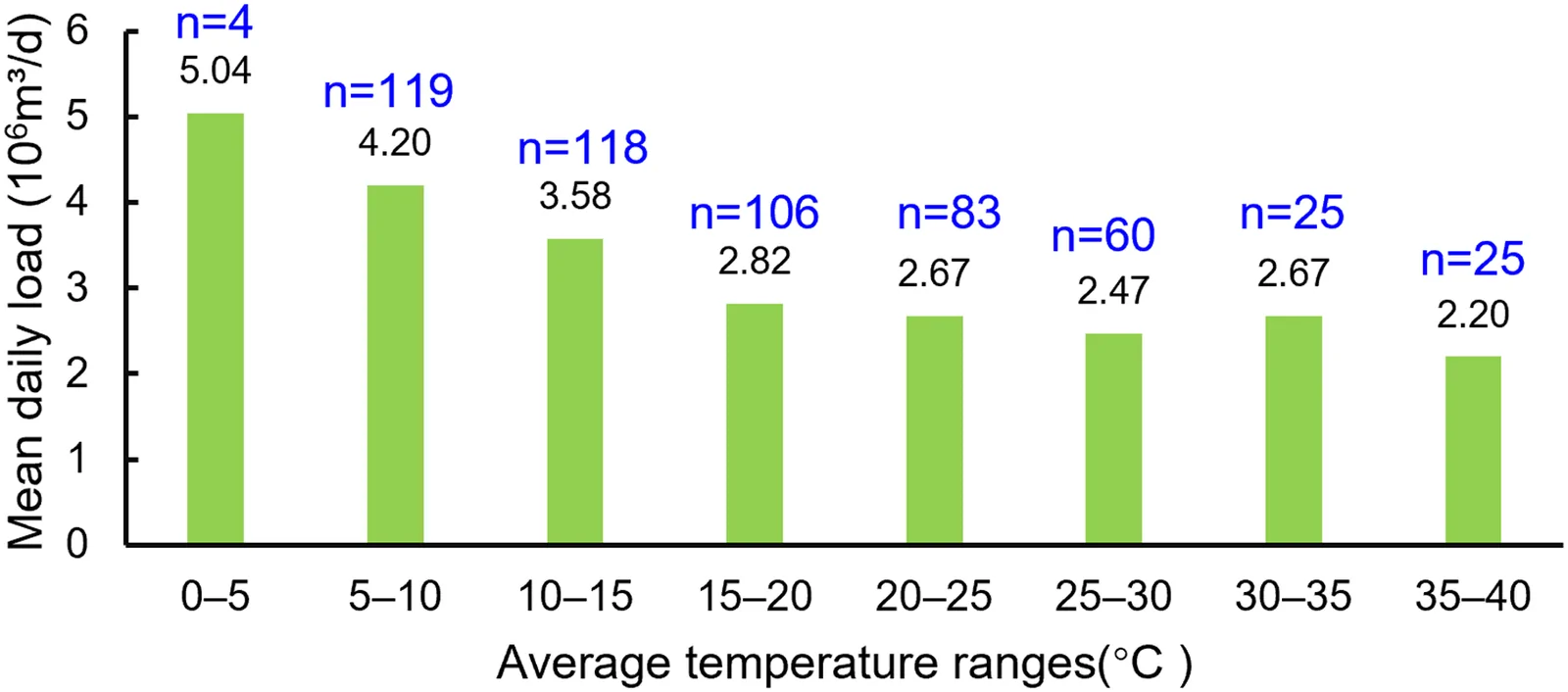
Mean daily load across daily average temperature ranges at Station F.

**Fig 7:**
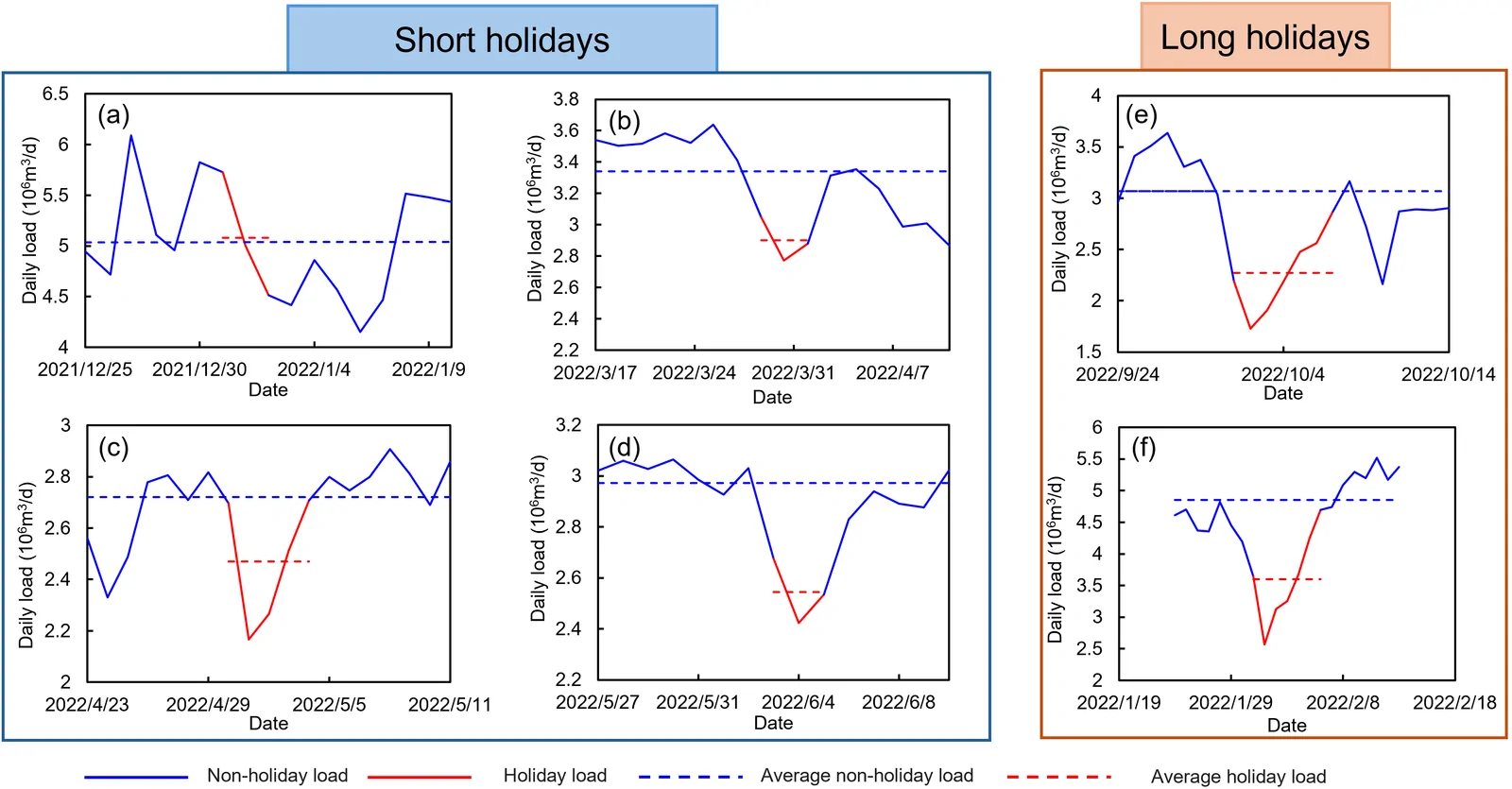
Daily gas load during holidays and non-holiday periods at Station F.

**Fig 8:**
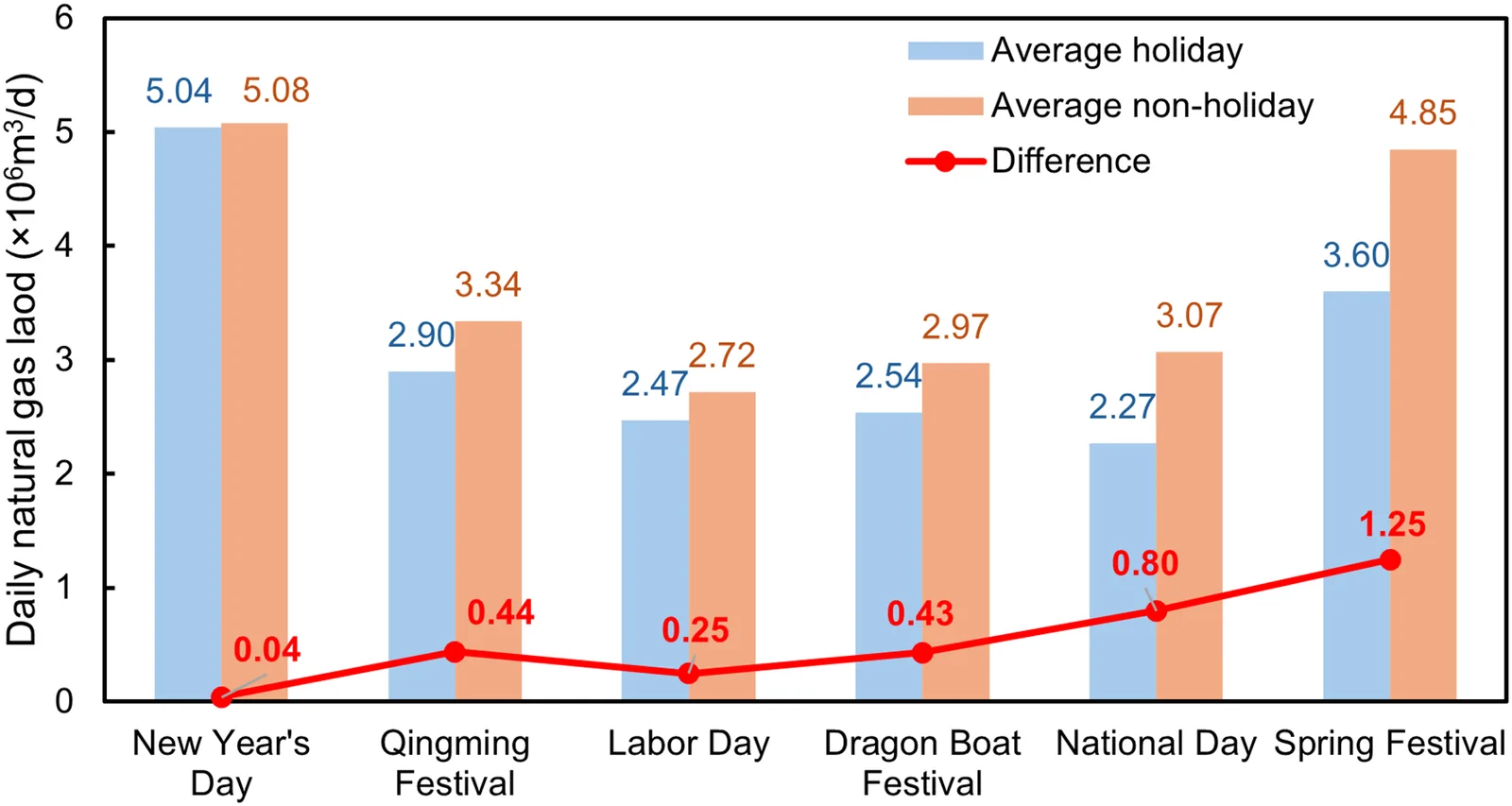
Average daily gas load and differences between holiday and non-holiday periods at Station F.

### 3.2 Feature construction and dimensionality reduction

#### 3.2.1 Effectiveness of wavelet threshold denoising.

Fig 9 shows the effect of wavelet denoising on representative daily load series. Denoising attenuated isolated spikes and short irregular oscillations while preserving the broader load profile and recurrent rhythms.

**Fig 9:**
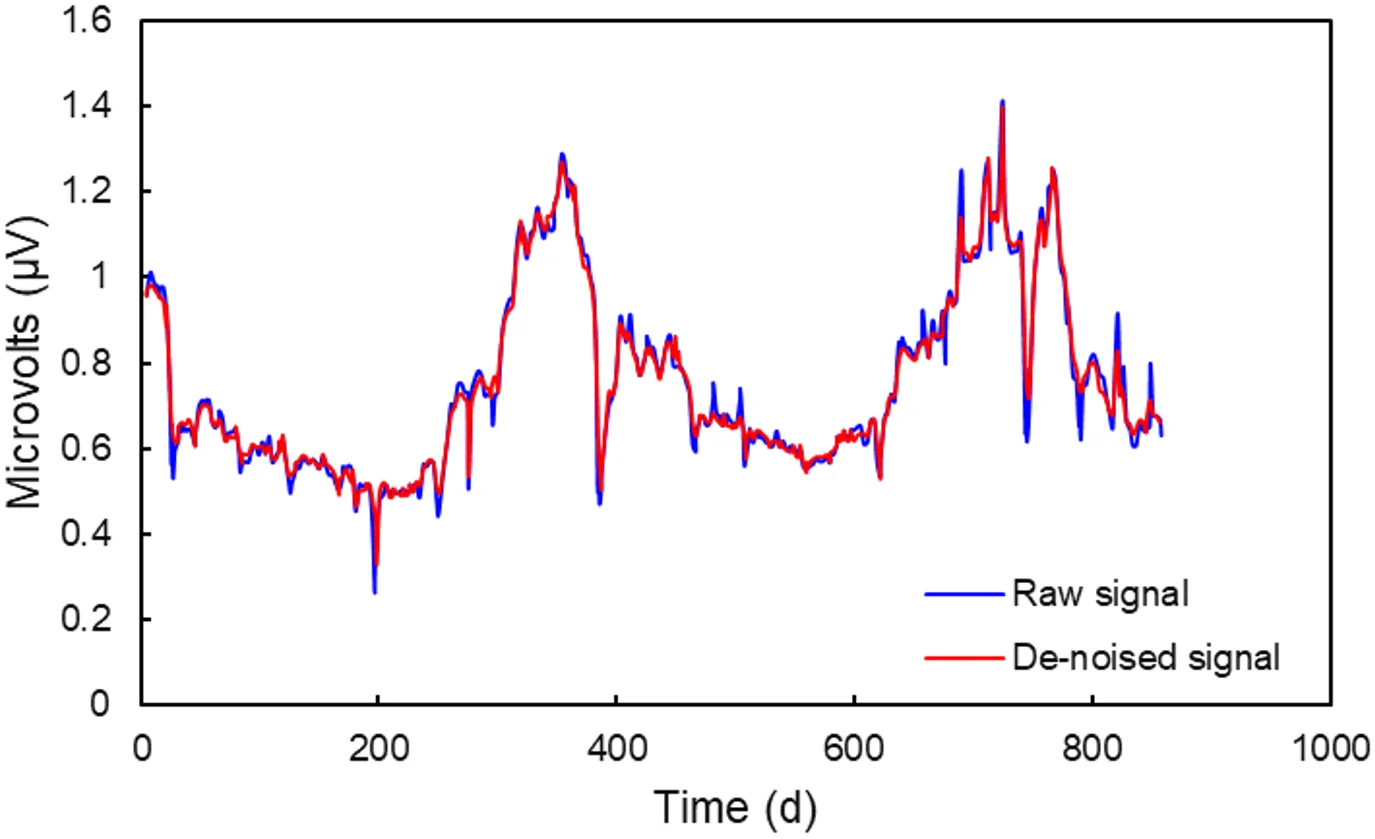
Wavelet denoising of representative daily load series.

#### 3.2.2 Final feature representation and temperature compression.

Temperature compression reduced the three correlated measurements to a single principal component in the final input, alongside the denoised load and calendar features.

### 3.3 Forecasting performance and forecastability boundary

#### 3.3.1 Overall station-level forecasting accuracy.

Table 8 shows station-level MAPE and MedianAPE for CNN-BiLSTM-Attention under the 30-day walk-forward evaluation. Mean station-level MAPE across the nine routine evaluable stations was 11.77%.

**Table 8:** Station-level MAPE and MedianAPE of CNN-BiLSTM-Attention forecasts.

Station	MAPE (%)	MedianAPE (%)	Evaluation status
A	4.84	4.09	Routine evaluable
B	21.03	15.29	Routine evaluable
C	12.86	8.70	Routine evaluable
D	6.55	4.99	Routine evaluable
E	18.53	15.76	Routine evaluable
F	19.66	17.37	Routine evaluable
G	51.42	34.47	Data-quality boundary
H	10.88	8.12	Routine evaluable
I	5.52	4.13	Routine evaluable
J	6.10	5.14	Routine evaluable
K	–	–	Insufficient history (62 days)

Predictability varied with demand regularity and telemetry continuity. CNN-BiLSTM-Attention achieved MAPEs of 4.84%, 5.52%, and 6.10% at Stations A, I, and J, respectively, and values above 18% at Stations B, E, and F. Station G reached 51.42%, marking the practical boundary associated with its sparse telemetry.

#### 3.3.2 Multi-baseline comparison under walk-forward evaluation.

Table 9 shows station-wise metrics averaged across the six forecasting models. S1–S4 Tables show all station-model metrics, and S5 Table shows the model-wise bootstrap summary.

**Table 9:** Station-wise mean forecasting metrics across six representative models.

Station No.	Mean MAPE (%)	Mean MedianAPE (%)	Mean MAE (10⁴ m³/d)	Mean RMSE (10⁴ m³/d)
A	4.54	3.94	14.97	17.84
B	18.45	9.75	47.89	74.48
C	11.31	9.11	1.60	1.98
D	5.74	3.98	22.28	29.23
E	15.16	11.61	0.24	0.32
F	16.89	13.84	37.24	45.88
G	48.00	28.09	19.87	24.29
H	11.13	9.30	2.58	3.17
I	4.15	3.31	3.68	4.65
J	5.15	4.33	20.19	24.04

The model-averaged results showed a consistent station-difficulty pattern. Stations A, I, and J had the lowest mean MAPE, while Stations B, E, and F were difficult for all models.

Across the nine routine stations, mean MAPE was 8.61% for SARIMAX, 8.95% for XGBoost, 9.78% for GRU, 10.93% for LSTM, 11.63% for BPNN, and 11.77% for CNN-BiLSTM-Attention. Paired differences relative to CNN-BiLSTM-Attention were −3.16 percentage points for SARIMAX (95% CI: −4.72 to −1.63), −2.82 for XGBoost (−4.10 to −1.67), −1.99 for GRU (−2.75 to −1.35), −0.85 for LSTM (−1.94 to 0.31), and −0.15 for BPNN (−1.37 to 0.93).

The best-performing model differed by station. SARIMAX and XGBoost were competitive at several regular and volatile stations, while recurrent and hybrid models performed well at other sites. These results support station-specific model selection for prospective deployment, with CNN-BiLSTM-Attention considered alongside the other candidate models.

#### 3.3.3 Representative forecast trajectories at selected stations.

Fig 10 shows CNN-BiLSTM-Attention hold-out trajectories for Stations A, F, and G. Agreement was closest at Station A. Predictions smoothed much of Station F's volatility, while Station G showed large amplitude and shape errors consistent with its limited historical continuity.

**Fig 10:**
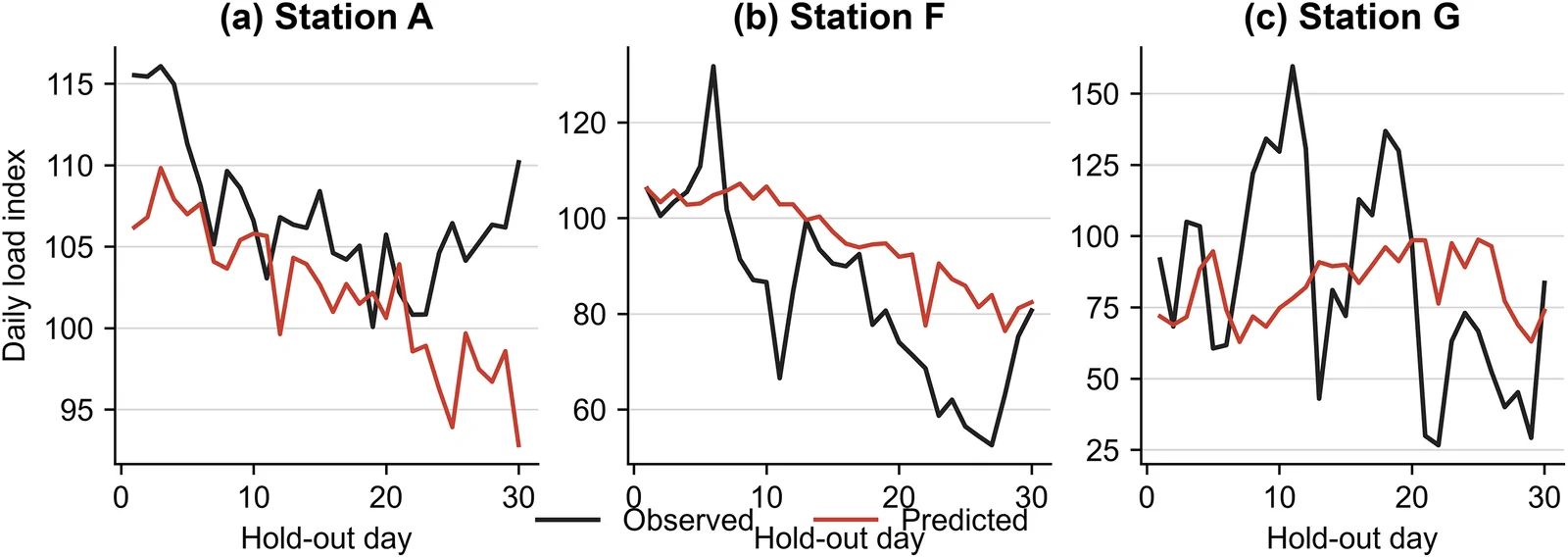
Observed and predicted daily gas-load trajectories at representative stations. A) Station A. B) Station F. C) Station G.

S3 Fig shows training and validation MSE at the first hold-out origin for Stations A, F, and G. Convergence differed by station, and the data-sparse Station G had the largest validation loss.

### 3.4 Daily-to-hourly demand reconstruction and temporal applicability

Table 10 shows the archived 18-day temporal-reconstruction results for LFFX, NGXDD, and DLFX. Hourly MAPE was 7.5%, 6.7%, and 8.1%, respectively. Peak-timing errors were 1.0, 1.0, and 1.5 h, and peak-demand magnitude errors were 5.6%, 5.1%, and 6.6%, respectively.

**Table 10:** Archived 18-day temporal-reconstruction validation summary.

Pipeline segment	Validation days (n)	Hourly MAPE (%)	Peak-timing error (h)	Peak-demand magnitude error (%)
West Pipeline-LFFX	18	7.5	1.0	5.6
East Pipeline-NGXDD	18	6.7	1.0	5.1
East Pipeline-DLFX	18	8.1	1.5	6.6

Fig 11 shows the reconstructed and observed hourly profiles for the three segments. LFFX and NGXDD agreed closely with observations over most hours, with an approximately 1-h shift near the morning peak. DLFX showed a larger mismatch around its principal peak.

**Fig 11:**
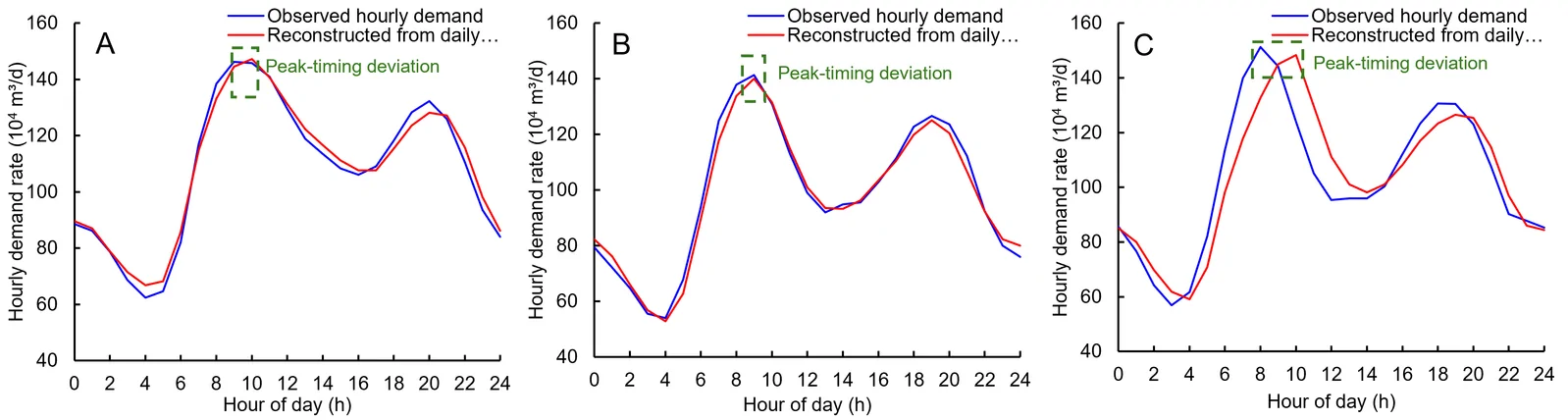
Validation of the daily-to-hourly demand transformation for the three pipeline segments. A) West Pipeline-LFFX. B) East Pipeline-NGXDD. C) East Pipeline-DLFX.

### 3.5 Ablation study: Contribution of architectural components

Table 11 shows ablation results for Stations A, F, and G at the same 30 walk-forward origins. Each variant used the common inputs, preprocessing sequence, and training protocol.

**Table 11:** Ablation results for representative Stations A, F, and G.

Model variant	Station A MAPE (%)	Station F MAPE (%)	Station G MAPE (%)
BiLSTM only	7.29	15.69	49.64
CNN only	2.99	16.21	51.76
Attention only	3.12	15.78	46.43
CNN-BiLSTM	6.15	15.48	52.90
BiLSTM-Attention	3.82	18.52	56.49
CNN-Attention	5.94	20.32	58.72
CNN-BiLSTM-Attention	4.84	19.66	51.42

At Station A, CNN only and attention only achieved MAPEs of 2.99% and 3.12%, compared with 4.84% for the full model. At Station F, CNN-BiLSTM and BiLSTM only achieved 15.48% and 15.69%, compared with 19.66% for the full model. Added architectural complexity was not consistently beneficial at the available sample size.

All Station G variants remained operationally inadequate: MAPE ranged from 46.43% for attention only to 58.72% for CNN-Attention, with 51.42% for the full model. Architecture changes did not overcome sparse telemetry.

The ablation results support simpler models where station-level validation favors them. At data-impaired sites, telemetry repair is the priority for improving prospective forecasts.

### 3.6 Archived engineering linepack target-setting results

Table 12 and Fig 12 show the selected preparation values for the three historical engineering cases. The LFFX, NGXDD, and DLFX linepack targets were 1.272, 4.702, and 1.571 × 10⁶ m³, respectively, and passed case-specific deterministic inventory checks. These targets imply packing for LFFX and drafting for NGXDD and DLFX. The NGXDD pressure of 4.86 MPa is an archived reported value; its conversion could not be independently reproduced from the available records.

**Table 12:** Selected linepack preparation values and corresponding pressures.

Pipeline segment	Current linepack (10⁶ m³)	Archived selected preparation (10⁶ m³)	Current pressure (MPa)	Preparation-target pressure (MPa)	Strategy
LFFX	1.151	1.272	2.98	3.23	Packing
NGXDD	5.303	4.702	5.31	4.86	Drafting
DLFX	1.729	1.571	5.19	4.79	Drafting

**Fig 12:**
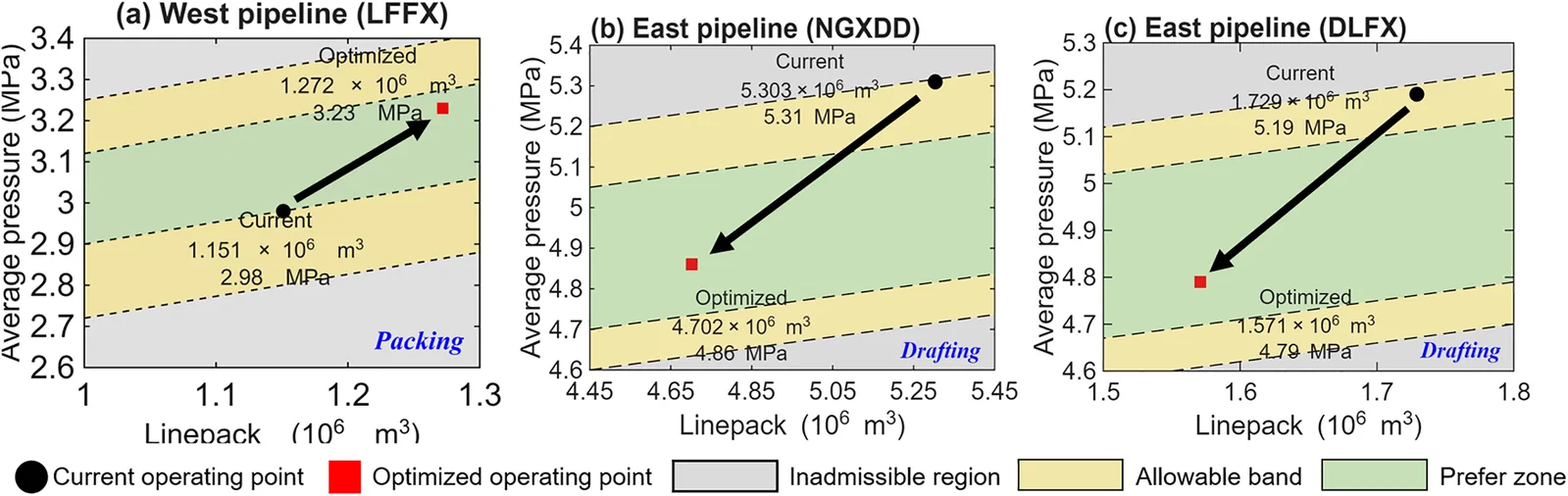
Observed and selected preparation points. (A) West pipeline-LFFX. (B) East pipeline-NGXDD. (C) East pipeline-DLFX. Colored bands show the prescribed linepack-pressure envelopes. NGXDD pressure: archived reported value.

For LFFX, the observed state lies near the lower boundary of the preferred zone. The preparation target increases the linepack reserve within the historical operating scenario [29].

The DLFX selection was 1,571,469.794304801 m³. With supply and withdrawal baselines of 530 × 10⁴ m³/d and the corresponding safe-envelope calibration, the continuous minimizing set was 1,559,382.189–1,573,735.267 m³. The selected value attained the minimum mean absolute deviation of 71,567.257 m³. All 25 inventory states and their associated pressure envelopes satisfied the constraints, including the terminal state. The equivalent-pipe pressure conversion, with a 1,000 m³ stopping tolerance, returned 4.792964230 MPa, consistent with the reported 4.79 MPa.

For NGXDD, inlet-supply and daily withdrawal baselines were both 480 × 10⁴ m³/d. The hourly withdrawal profile was reconstructed using the historical unevenness factors. The selected preparation value of 4,701,756.095919851 m³ lay within the continuous minimizing interval of 4,695,278.411–4,707,161.027 m³. It attained the minimum mean absolute deviation of 64,746.628 m³. All 25 inventory states satisfied the inventory constraints, supporting the reported preparation value of 4.702 × 10⁶ m³.

S4 Fig shows best-so-far objective histories for the support-based PSO diagnostic, as mean and standard deviation across 30 runs. All three objectives stabilized within 10 iterations. LFFX reached the same minimum in every run, consistent with an equivalent-minimum interval. NGXDD dispersion decreased over the first several iterations, while DLFX retained minor dispersion.

Table 13 shows the PSO results for the three computational budgets and the deterministic grid references. PSO results are reported as mean ± standard deviation over 30 runs. Table 12 shows the selected operating targets separately from these solver statistics. Under the 20-particle, 10-iteration setting, mean differences from the sampled-grid objective minima were 0.000%, 0.172%, and 0.008% for LFFX, NGXDD, and DLFX, respectively. The two optimal LFFX grid samples, 1.2712 and 1.2812 × 10⁶ m³, differ from the full continuous minimizing interval of approximately 1.269828333–1.289124243 × 10⁶ m³.

**Table 13:** Repeated-run PSO diagnostics and sampled-grid references.

Segment	Metric	20 × 10	40 × 10	20 × 30	Deterministic grid
LFFX	Objective (10⁴ m³)	6.4169 ± 0.0000	6.4169 ± 0.0000	6.4169 ± 0.0000	6.4169
	Preparation value (10⁶ m³)	1.2799 ± 0.0007	1.2798 ± 0.0006	1.2799 ± 0.0007	1.2712, 1.2812
NGXDD	Objective (10⁴ m³)	6.4858 ± 0.0132	6.4791 ± 0.0035	6.4753 ± 0.0008	6.4747
	Preparation value (10⁶ m³)	4.7028 ± 0.0010	4.7029 ± 0.0003	4.7030 ± 0.0001	4.7030
DLFX	Objective (10⁴ m³)	7.1573 ± 0.0005	7.1571 ± 0.0006	7.1568 ± 0.0001	7.1567
	Preparation value (10⁶ m³)	1.5692 ± 0.0001	1.5693 ± 0.0001	1.5693 ± 0.0000	1.5693

The West-pipeline case for 22 April 2023 was reconstructed from historical station-demand inputs, participation coefficients, and engineering calculation records. The forecast origin was the end of 21 April 2023. Table 14 shows the calculation sequence for the 22 April 2023 West-pipeline case. S6 Table shows the input sources and full-precision numerical trace. Whether these historical inputs match predictions from the current common 30-day benchmark remains unverified.

**Table 14:** Numerical trace of the reconstructed West-pipeline engineering case for 22 April 2023.

Stage or quantity	Input or rule	Result
Station forecasts	Stations J, E, K, H, I; coefficients 1, 1, 1, 1, 0.5	Individual forecasts and contributions are listed in S6 Table.
Raw aggregation	Weighted sum of the five station forecasts	4,817,675.298713333 m³/d
Engineering rounding	Round the aggregate to two decimal places in 10⁴ m³/d before disaggregation	481.77 × 10⁴ m³/d
Hourly reconstruction	Use the high-precision β factors used in the reconstruction; sum = 24, mean = 1	24 withdrawal rates matching Table 4
Inventory propagation	Constant supply of 481.77 × 10⁴ m³/d; Eq. (28), Δt = 1/24 day	25 boundary states and a continuous optimal interval of 1,269,828.333–1,289,124.243 m³
Archived selected member	Verify the retained PSO point against the independently computed optimal set	1,271,536.975841198 m³; minimum mean absolute deviation 64,168.628 m³
Pressure and preparation	Archived equivalent-pipe conversion with 1,000 m³ stopping tolerance	3.231302521 MPa; packing relative to 1,151,150.949 m³

The daily mean demand forecasts were 4,097,629.44125, 13,405.20833, 84,157.875, 205,933.9583, and 833,097.631666666 m³/d for Stations J, E, K, H, and I, respectively. Their West-pipeline participation coefficients were 1, 1, 1, 1, and 0.5. The station-level forecasts were first aggregated using this participation rule, after which the aggregated daily demand was temporally disaggregated into the 24-h LFFX withdrawal profile.

Station K's historical forecast is used only in this case reconstruction and is excluded from model-level performance statistics.

The weighted station forecasts gave an unrounded daily aggregate of 4,817,675.298713333 m³/d, or 481.7675298713333 × 10⁴ m³/d. The engineering implementation rounded this aggregate to two decimal places in units of 10⁴ m³/d, giving a baseline of 481.77 × 10⁴ m³/d. Multiplication by the 24 full-precision hourly unevenness factors reproduced the withdrawal rates in Table 4 at the reported precision.

The inlet supply was constant at 481.77 × 10⁴ m³/d. Eq. (28), with Δt = 1/24 day, propagated each candidate preparation value through the 24 hourly intervals. Quadratic safe-linepack envelopes were reconstructed from the archived throughput calibration at 100–800 × 10⁴ m³/d and evaluated at the mean inlet and withdrawal rate. Their midpoint defined the hourly reference in Eq. (25). The terminal state was checked against the final hourly envelope.

The median-set calculation yielded a continuous optimal interval of approximately 1,269,828.333–1,289,124.243 m³. The PSO-selected preparation value, 1,271,536.975841198 m³, lay within this feasible interval and attained the minimum mean absolute deviation of 64,168.628 m³.

The observed state was 1,151,150.949 m³ at a reported average pressure of 2.98 MPa. The selected preparation value was higher by 120,386.027 m³, supporting a packing recommendation. The pressure conversion used an equivalent diameter of 711 mm, length of 91.47 km, and temperature of 293.15 K. Its compressibility factor of 0.938948103 was fixed at the baseline state. An iterative search with a 1,000 m³ volume tolerance returned 3.231302521 MPa, with a volume residual of approximately 37.7 m³. The reported preparation values were therefore 1.272 × 10⁶ m³ and 3.23 MPa. The observed linepack came from the archived topology workbook, whereas pressure conversion used the archived equivalent-pipe approximation.

## 4. Engineering implications and limitations

The workflow links station forecasts to segment inventory preparation through explicit hourly withdrawal profiles. The historical targets imply packing for LFFX and drafting for NGXDD and DLFX. Station-dependent forecast errors and intraday profile shifts make model selection and coefficient updating central to prospective use.

The selected linepack is a segment-level preparation reference for the start of the next-day horizon. The reduced-order calculation checks inventory and average-pressure envelopes under fixed boundary conditions. It omits actuator sequences, valve switching, compressor dispatch, flow reversal, nodal pressure redistribution, and transient dynamics. Compressor fuel, procurement cost, and pressure-fluctuation penalties are outside the objective. Operational implementation requires current boundary data, operator review, and transient hydraulic verification. MPC was not benchmarked because the formulation lacks the closed-loop dynamic states and control actions needed for a matched comparison.

Forecast and reconstruction errors were evaluated separately, without quantifying their propagation into transient pressure and flow responses. The effects of the observed 1.0–1.5 h peak-timing errors on preparation linepack and average-pressure targets were not quantified. Broader multi-day and multi-season end-to-end validation requires synchronized forecast, withdrawal, supply, and linepack-state records. At data-impaired sites such as Station G, telemetry repair remains a priority for reliable forecasting.

## 5. Conclusions

This study evaluated a forecast-to-dispatch workflow linking daily station forecasting, hourly withdrawal-profile construction, and reduced-order segment-level linepack target setting for the Chongqing regional gas network.

Forecast accuracy varied substantially across stations. Among nine routine stations, SARIMAX achieved the lowest mean MAPE, 8.61%, compared with 11.77% for CNN-BiLSTM-Attention. Station G had a CNN-BiLSTM-Attention MAPE of 51.42%. Baseline and ablation results support station-specific model selection for prospective deployment and highlight the forecasting limits imposed by sparse telemetry.

The independent archived 18-day reconstruction dataset reported hourly MAPE of 6.7%−8.1% and peak-timing deviations of 1.0–1.5 h. The hourly interface supplies the withdrawal profiles needed for segment-level inventory preparation.

The retrospective LFFX case and the separate NGXDD and DLFX cases supported linepack preparation values of 1.272, 4.702, and 1.571 × 10⁶ m³, respectively. All three values passed case-specific deterministic inventory checks. Operational implementation requires current boundary data and transient hydraulic verification.

## Supporting information

S1 Table Station-wise MAPE of representative forecasting models. (DOCX)

S2 Table Station-wise MedianAPE of representative forecasting models. (DOCX)

S3 Table Station-wise MAE of representative forecasting models. (DOCX)

S4 Table Station-wise RMSE of representative forecasting models. (DOCX)

S5 Table Model-wise forecasting performance across the nine routine evaluable stations (A–F and H–J). Values include mean MAPE and paired differences relative to CNN-BiLSTM-Attention with 95% bootstrap confidence intervals.
(DOCX)

S6 Table Historical West-pipeline reconstruction and case-specific verification of the three archived linepack selections. (DOCX)

S1 Fig Throughput-dependent linepack control-zone envelopes of the west pipeline segment (LFFX). (TIF)

S2 Fig Throughput-dependent linepack control-zone envelopes of the east pipeline segments. (a) NGXDD. (b) DLFX.
(TIF)

S3 Fig Training and validation MSE histories for representative Stations A, F, and G at the first hold-out origin. (TIF)

S4 Fig Best-so-far objective convergence for the support-based PSO diagnostic under the 20-particle, 10-iteration setting. Curves show the mean and standard deviation across 30 runs using interpolated objective-support values.
(TIF)

S1 Code Forecasting scripts, configurations and recorded outputs; illustrative hourly reconstruction; support-based PSO diagnostics; and the retrospective West-pipeline numerical reconstruction. (ZIP)

S1 Data Anonymized indexed daily series, forecasting outputs and metrics, archived reconstruction and linepack summaries, and documentation of processing-stage and data-access limits. (ZIP)
