# Supplementary material for: A forecast-to-dispatch workflow for day-ahead operation of a regional natural gas transmission network based on station-level load prediction and linepack target setting
Source: PLoS One. 2026 Oct 5;21(10):e0349981. doi: 10.1371/journal.pone.0349981 (PMC13637901; doi:10.1371/journal.pone.0349981)
Supplement: Supplementary file 1 [file pone.0349981.s001.docx]

**S1 Table. Station-wise MAPE of representative forecasting models.**

| **Station** | **CNN-BiLSTM-Attention (%)** | **SARIMAX (%)** | **XGBoost (%)** | **GRU (%)** | **LSTM (%)** | **BPNN (%)** |
| --- | --- | --- | --- | --- | --- | --- |
| A | 4.84 | 2.48 | 2.66 | 2.81 | 6.74 | 7.71 |
| B | 21.03 | 16.67 | 18.08 | 16.58 | 17.95 | 20.39 |
| C | 12.86 | 8.86 | 8.08 | 11.24 | 14.45 | 12.39 |
| D | 6.55 | 4.44 | 4.66 | 5.61 | 5.91 | 7.28 |
| E | 18.53 | 13.03 | 11.71 | 16.54 | 16.77 | 14.35 |
| F | 19.66 | 12.04 | 16.26 | 16.53 | 17.13 | 19.69 |
| G | 51.42 | 44.42 | 44.28 | 49.09 | 48.16 | 50.64 |
| H | 10.88 | 11.84 | 10.81 | 10.19 | 11.51 | 11.52 |
| I | 5.52 | 3.66 | 3.59 | 3.75 | 2.97 | 5.41 |
| J | 6.10 | 4.47 | 4.70 | 4.79 | 4.93 | 5.91 |
