# Supplementary material for: A forecast-to-dispatch workflow for day-ahead operation of a regional natural gas transmission network based on station-level load prediction and linepack target setting
Source: PLoS One. 2026 Oct 5;21(10):e0349981. doi: 10.1371/journal.pone.0349981 (PMC13637901; doi:10.1371/journal.pone.0349981)
Supplement: Supplementary file 2 [file pone.0349981.s002.docx]

**S2 Table. Station-wise MedianAPE of representative forecasting models.**

| **Station** | **CNN-BiLSTM-Attention (%)** | **SARIMAX (%)** | **XGBoost (%)** | **GRU (%)** | **LSTM (%)** | **BPNN (%)** |
| --- | --- | --- | --- | --- | --- | --- |
| A | 4.09 | 2.03 | 2.12 | 2.52 | 5.36 | 7.51 |
| B | 15.29 | 5.66 | 5.48 | 8.10 | 8.08 | 15.89 |
| C | 8.70 | 6.57 | 7.23 | 10.24 | 11.75 | 10.15 |
| D | 4.99 | 4.17 | 3.70 | 3.57 | 3.12 | 4.30 |
| E | 15.76 | 8.17 | 6.06 | 12.30 | 14.36 | 13.02 |
| F | 17.37 | 10.43 | 12.32 | 13.60 | 14.45 | 14.90 |
| G | 34.47 | 28.40 | 19.95 | 29.34 | 26.82 | 29.58 |
| H | 8.12 | 9.79 | 9.83 | 8.29 | 11.13 | 8.66 |
| I | 4.13 | 2.81 | 2.49 | 3.25 | 2.43 | 4.77 |
| J | 5.14 | 4.17 | 4.14 | 3.97 | 3.75 | 4.83 |
