# Supplementary material for: A forecast-to-dispatch workflow for day-ahead operation of a regional natural gas transmission network based on station-level load prediction and linepack target setting
Source: PLoS One. 2026 Oct 5;21(10):e0349981. doi: 10.1371/journal.pone.0349981 (PMC13637901; doi:10.1371/journal.pone.0349981)
Supplement: Supplementary file 3 [file pone.0349981.s003.docx]

**S3 Table. Station-wise MAE of representative forecasting models.**

| **Station** | **CNN-BiLSTM-Attention (10^4^ m^3^/d)** | **SARIMAX (10^4^ m^3^/d)** | **XGBoost (10^4^ m^3^/d)** | **GRU (10^4^ m^3^/d)** | **LSTM (10^4^ m^3^/d)** | **BPNN (10^4^ m^3^/d)** |
| --- | --- | --- | --- | --- | --- | --- |
| A | 16.05 | 8.08 | 8.73 | 9.29 | 22.20 | 25.50 |
| B | 52.76 | 43.90 | 48.20 | 43.62 | 46.82 | 52.00 |
| C | 1.81 | 1.31 | 1.14 | 1.60 | 2.03 | 1.74 |
| D | 25.22 | 17.38 | 18.31 | 21.82 | 22.86 | 28.07 |
| E | 0.29 | 0.20 | 0.18 | 0.26 | 0.27 | 0.24 |
| F | 42.12 | 29.25 | 36.05 | 36.51 | 37.09 | 42.42 |
| G | 23.32 | 18.28 | 16.94 | 20.26 | 19.89 | 20.52 |
| H | 2.48 | 2.72 | 2.51 | 2.44 | 2.69 | 2.64 |
| I | 4.87 | 3.25 | 3.21 | 3.31 | 2.66 | 4.78 |
| J | 23.79 | 17.66 | 18.57 | 18.83 | 19.39 | 22.92 |
