# Supplementary material for: A forecast-to-dispatch workflow for day-ahead operation of a regional natural gas transmission network based on station-level load prediction and linepack target setting
Source: PLoS One. 2026 Oct 5;21(10):e0349981. doi: 10.1371/journal.pone.0349981 (PMC13637901; doi:10.1371/journal.pone.0349981)
Supplement: Supplementary file 4 [file pone.0349981.s004.docx]

**S4 Table. Station-wise RMSE of representative forecasting models.**

| **Station** | **CNN-BiLSTM-Attention (10^4^ m^3^/d)** | **SARIMAX (10^4^ m^3^/d)** | **XGBoost (10^4^ m^3^/d)** | **GRU (10^4^ m^3^/d)** | **LSTM (10^4^ m^3^/d)** | **BPNN (10^4^ m^3^/d)** |
| --- | --- | --- | --- | --- | --- | --- |
| A | 19.78 | 10.11 | 10.70 | 11.30 | 26.29 | 28.85 |
| B | 68.85 | 79.40 | 80.92 | 73.19 | 75.27 | 69.27 |
| C | 2.31 | 1.77 | 1.41 | 1.91 | 2.36 | 2.14 |
| D | 33.18 | 22.28 | 23.05 | 28.88 | 31.32 | 36.64 |
| E | 0.36 | 0.25 | 0.25 | 0.34 | 0.36 | 0.32 |
| F | 53.18 | 36.80 | 43.84 | 43.27 | 45.77 | 52.41 |
| G | 27.07 | 23.36 | 22.51 | 23.89 | 24.32 | 24.61 |
| H | 3.21 | 3.37 | 3.10 | 3.04 | 3.08 | 3.24 |
| I | 5.87 | 4.45 | 4.15 | 4.18 | 3.42 | 5.82 |
| J | 28.89 | 20.49 | 21.87 | 21.98 | 22.63 | 28.39 |
