# Supplementary material for: A forecast-to-dispatch workflow for day-ahead operation of a regional natural gas transmission network based on station-level load prediction and linepack target setting
Source: PLoS One. 2026 Oct 5;21(10):e0349981. doi: 10.1371/journal.pone.0349981 (PMC13637901; doi:10.1371/journal.pone.0349981)
Supplement: Supplementary file 5 [file pone.0349981.s005.docx]

**S5 Table. Model-wise forecasting performance across the nine routine evaluable stations.**

| **Model** | **Mean MAPE (%, 95% CI)** | **Mean MedianAPE (%)** | **Mean MAE**  **(10^4^ m^3^/d)** | **Mean RMSE**  **(10^4^ m^3^/d)** | **Paired MAPE difference vs CNN-BiLSTM-Attention (percentage points, 95% CI)** |
| --- | --- | --- | --- | --- | --- |
| CNN-BiLSTM-Attention | 11.77 (7.85, 15.88) | 9.29 | 18.82 | 23.96 | Reference |
| SARIMAX | 8.61 (5.55, 11.79) | 5.98 | 13.75 | 19.88 | -3.16 (-4.72, -1.63) |
| XGBoost | 8.95 (5.70, 12.63) | 5.93 | 15.21 | 21.03 | -2.82 (-4.10, -1.67) |
| GRU | 9.78 (6.32, 13.33) | 7.32 | 15.30 | 20.90 | -1.99 (-2.75, -1.35) |
| LSTM | 10.93 (7.40, 14.44) | 8.27 | 17.33 | 23.39 | -0.85 (-1.94, 0.31) |
| BPNN | 11.63 (8.32, 15.28) | 9.34 | 20.03 | 25.23 | -0.15 (-1.37, 0.93) |
