# Supplementary material for: A forecast-to-dispatch workflow for day-ahead operation of a regional natural gas transmission network based on station-level load prediction and linepack target setting
Source: PLoS One. 2026 Oct 5;21(10):e0349981. doi: 10.1371/journal.pone.0349981 (PMC13637901; doi:10.1371/journal.pone.0349981)
Supplement: Supplementary file 6 [file pone.0349981.s006.docx]

**S6 Table. Historical West-pipeline reconstruction and case-specific verification of the three archived linepack selections.**

West-pipeline forecast target: 22 April 2023; forecast origin: end of 21 April 2023. Sources: revised manuscript, archived-engineering verification records, and hourly schedules in Tables 4 and 5.

**S6A. West-pipeline station inputs and aggregation**

| **Station** | **Source label** | **Daily forecast (m³/d)** | **Participation coefficient** | **Contribution (m³/d)** | **Contribution (10⁴ m³/d)** |
| --- | --- | --- | --- | --- | --- |
| J | XP | 4097629.44125 | 1 | 4097629.44125 | 409.762944125 |
| E | GLS | 13405.20833 | 1 | 13405.20833 | 1.340520833 |
| K | ZL | 84157.875 | 1 | 84157.875 | 8.4157875 |
| H | LF | 205933.9583 | 1 | 205933.9583 | 20.59339583 |
| I | TYW | 833097.631666666 | 0.5 | 416548.815833333 | 41.6548815833333 |

The aggregated daily demand was rounded to two decimal places in units of 10⁴ m³/d before hourly disaggregation.

| **Quantity** | **Value** | **Unit** |
| --- | --- | --- |
| Raw weighted aggregate | 4817675.298713333 | m³/d |
| Raw aggregate in 10⁴ m³/d | 481.7675298713333 | 10⁴ m³/d |
| Engineering rounded baseline | 481.77 | 10⁴ m³/d |
| Engineering rounded baseline | 4817700 | m³/d |
| Selected LFFX preparation value | 1271536.975841198 | m³ |
| Observed/current LFFX linepack | 1151150.949 | m³ |
| Preparation difference | 120386.02684119786 | m³ |
| Strategy | Packing |  |
| Raw aggregate expected | 4817675.298713333 | m³/d |

Hourly factors in this supplement are reconstructed from the reported hourly schedule divided by its documented daily baseline. They reproduce Tables 4 and 5 at reported precision; they are not presented as literal archived worksheet-cell values.

**S6B. LFFX hourly reconstruction and inventory trajectory**

βₖ = Table 4 hourly rate / 481.77; qout,ₖ = 4,817,700βₖ m³/d; qin,ₖ = 4,817,700 m³/d.

ΔVₖ = (qin,ₖ − qout,ₖ)/24; C₀ = 0; Cₖ₊₁ = Cₖ + ΔVₖ; Vₖ = selected preparation + Cₖ. The 24 rows include the 25 states V₀–V₂₄. Factors are displayed to nine decimals and inventory values to three decimals; calculation uses the unrounded worksheet formulas.

| **k** | **Hour** | **Table 4 rate (10⁴ m³/d)** | **Reconstructed βₖ** | **βₖ (2 d.p.)** | **qout,ₖ (m³/d)** | **qin,ₖ (m³/d)** | **ΔVₖ (m³)** | **Cₖ (m³)** | **Vₖ (m³)** | **Vₖ₊₁ (m³)** |
| --- | --- | --- | --- | --- | --- | --- | --- | --- | --- | --- |
| 0 | 00:00 | 424.98 | 0.882122174 | 0.88 | 4249800.000 | 4817700 | 23662.500 | 0.000 | 1271536.976 | 1295199.476 |
| 1 | 01:00 | 430.79 | 0.894181871 | 0.89 | 4307900.000 | 4817700 | 21241.667 | 23662.500 | 1295199.476 | 1316441.143 |
| 2 | 02:00 | 440.88 | 0.915125475 | 0.92 | 4408800.000 | 4817700 | 17037.500 | 44904.167 | 1316441.143 | 1333478.643 |
| 3 | 03:00 | 451.13 | 0.936401187 | 0.94 | 4511300.000 | 4817700 | 12766.667 | 61941.667 | 1333478.643 | 1346245.309 |
| 4 | 04:00 | 507.58 | 1.053573282 | 1.05 | 5075800.000 | 4817700 | -10754.167 | 74708.333 | 1346245.309 | 1335491.143 |
| 5 | 05:00 | 560.57 | 1.163563526 | 1.16 | 5605700.000 | 4817700 | -32833.333 | 63954.167 | 1335491.143 | 1302657.809 |
| 6 | 06:00 | 557.87 | 1.157959192 | 1.16 | 5578700.000 | 4817700 | -31708.333 | 31120.833 | 1302657.809 | 1270949.476 |
| 7 | 07:00 | 553.87 | 1.149656475 | 1.15 | 5538700.000 | 4817700 | -30041.667 | -587.500 | 1270949.476 | 1240907.809 |
| 8 | 08:00 | 548.50 | 1.138510077 | 1.14 | 5485000.000 | 4817700 | -27804.167 | -30629.167 | 1240907.809 | 1213103.643 |
| 9 | 09:00 | 540.75 | 1.122423563 | 1.12 | 5407500.000 | 4817700 | -24575.000 | -58433.333 | 1213103.643 | 1188528.643 |
| 10 | 10:00 | 534.76 | 1.109990244 | 1.11 | 5347600.000 | 4817700 | -22079.167 | -83008.333 | 1188528.643 | 1166449.476 |
| 11 | 11:00 | 531.40 | 1.103015962 | 1.10 | 5314000.000 | 4817700 | -20679.167 | -105087.500 | 1166449.476 | 1145770.309 |
| 12 | 12:00 | 525.73 | 1.091246861 | 1.09 | 5257300.000 | 4817700 | -18316.667 | -125766.667 | 1145770.309 | 1127453.643 |
| 13 | 13:00 | 472.94 | 0.981671752 | 0.98 | 4729400.000 | 4817700 | 3679.167 | -144083.333 | 1127453.643 | 1131132.809 |
| 14 | 14:00 | 468.02 | 0.971459410 | 0.97 | 4680200.000 | 4817700 | 5729.167 | -140404.167 | 1131132.809 | 1136861.976 |
| 15 | 15:00 | 470.41 | 0.976420284 | 0.98 | 4704100.000 | 4817700 | 4733.333 | -134675.000 | 1136861.976 | 1141595.309 |
| 16 | 16:00 | 472.75 | 0.981277373 | 0.98 | 4727500.000 | 4817700 | 3758.333 | -129941.667 | 1141595.309 | 1145353.643 |
| 17 | 17:00 | 470.88 | 0.977395853 | 0.98 | 4708800.000 | 4817700 | 4537.500 | -126183.333 | 1145353.643 | 1149891.143 |
| 18 | 18:00 | 468.19 | 0.971812276 | 0.97 | 4681900.000 | 4817700 | 5658.333 | -121645.833 | 1149891.143 | 1155549.476 |
| 19 | 19:00 | 465.16 | 0.965522967 | 0.97 | 4651600.000 | 4817700 | 6920.833 | -115987.500 | 1155549.476 | 1162470.309 |
| 20 | 20:00 | 417.17 | 0.865911119 | 0.87 | 4171700.000 | 4817700 | 26916.667 | -109066.667 | 1162470.309 | 1189386.976 |
| 21 | 21:00 | 417.76 | 0.867135770 | 0.87 | 4177600.000 | 4817700 | 26670.833 | -82150.000 | 1189386.976 | 1216057.809 |
| 22 | 22:00 | 415.90 | 0.863275007 | 0.86 | 4159000.000 | 4817700 | 27445.833 | -55479.167 | 1216057.809 | 1243503.643 |
| 23 | 23:00 | 414.49 | 0.860348299 | 0.86 | 4144900.000 | 4817700 | 28033.333 | -28033.333 | 1243503.643 | 1271536.976 |

β sum: 24; β mean: 1; Mean hourly rate: 481.77 10⁴ m³/d; Terminal V24: 1271536.976 m³; V24 - V0: 0.000 m³.

**S6C. Case-specific verification of archived linepack selections**

| **Quantity** | **LFFX** | **NGXDD** | **DLFX** |
| --- | --- | --- | --- |
| Supply baseline (10⁴ m³/d) | 481.77 | 480 | 530 |
| Withdrawal baseline (10⁴ m³/d) | 481.77 | 480 | 530 |
| Current linepack (m³) | 1151150.949 | 5303000 | 1729300 |
| Selected preparation (m³) | 1271536.975841198 | 4701756.095919851 | 1571469.794304801 |
| Optimal interval low (m³) | 1269828.333 | 4695278.411 | 1559382.189 |
| Optimal interval high (m³) | 1289124.243 | 4707161.027 | 1573735.267 |
| Selected in interval? | Yes | Yes | Yes |
| Minimum MAD (m³) | 64168.628 | 64746.628 | 71567.257 |
| Current pressure (MPa) | 2.98 | 5.31 | 5.19 |
| Preparation pressure (MPa) | 3.231302521 | 4.86 | 4.79296423 |
| Pressure status | Independently replayed | Archived reported value; conversion worksheet unavailable | Independently replayed |
| Inventory status | 25-state inventory trace reproduced in S6B | 25-state inventory trace reproduced in S6D | 25-state inventory trace reproduced in S6E |
| Strategy | Packing | Drafting | Drafting |
| Source/basis | 22 Apr 2023 West reconstruction | Segment-specific 480/480 baseline | Segment-specific 530/530 baseline |

Pressure replay status follows the revised manuscript: LFFX, 3.231302521 MPa (reported as 3.23 MPa); DLFX, 4.792964230 MPa (reported as 4.79 MPa). NGXDD, 4.86 MPa, is retained from the archived engineering record because the matching pressure-conversion worksheet was unavailable. The membership checks below use the supplied minimizing intervals; they do not recalculate those intervals or replay pressure conversion.

**S6D. NGXDD hourly reconstruction and inventory trajectory**

βₖ = Table 5 hourly rate / 960; qout,ₖ = 480 × 10⁴βₖ m³/d; qin,ₖ = 480 × 10⁴ m³/d.

ΔVₖ = (qin,ₖ − qout,ₖ)/24; C₀ = 0; Cₖ₊₁ = Cₖ + ΔVₖ; Vₖ = selected preparation + Cₖ. The 24 rows include the 25 states V₀–V₂₄. Factors are displayed to nine decimals and inventory values to three decimals; calculation uses the unrounded worksheet formulas.

| **k** | **Hour** | **Table 5 rate (10⁴ m³/d)** | **Reconstructed βₖ** | **Segment rate (10⁴ m³/d)** | **qout,ₖ (m³/d)** | **qin,ₖ (m³/d)** | **ΔVₖ (m³)** | **Cₖ (m³)** | **Vₖ (m³)** | **Vₖ₊₁ (m³)** |
| --- | --- | --- | --- | --- | --- | --- | --- | --- | --- | --- |
| 0 | 00:00 | 846.84 | 0.882125000 | 423.420000 | 4234200.000 | 4800000 | 23575.000 | 0.000 | 4701756.096 | 4725331.096 |
| 1 | 01:00 | 858.41 | 0.894177083 | 429.205000 | 4292050.000 | 4800000 | 21164.583 | 23575.000 | 4725331.096 | 4746495.679 |
| 2 | 02:00 | 878.53 | 0.915135417 | 439.265000 | 4392650.000 | 4800000 | 16972.917 | 44739.583 | 4746495.679 | 4763468.596 |
| 3 | 03:00 | 898.95 | 0.936406250 | 449.475000 | 4494750.000 | 4800000 | 12718.750 | 61712.500 | 4763468.596 | 4776187.346 |
| 4 | 04:00 | 942.41 | 0.981677083 | 471.205000 | 4712050.000 | 4800000 | 3664.583 | 74431.250 | 4776187.346 | 4779851.929 |
| 5 | 05:00 | 932.59 | 0.971447917 | 466.295000 | 4662950.000 | 4800000 | 5710.417 | 78095.833 | 4779851.929 | 4785562.346 |
| 6 | 06:00 | 937.36 | 0.976416667 | 468.680000 | 4686800.000 | 4800000 | 4716.667 | 83806.250 | 4785562.346 | 4790279.013 |
| 7 | 07:00 | 1103.66 | 1.149645833 | 551.830000 | 5518300.000 | 4800000 | -29929.167 | 88522.917 | 4790279.013 | 4760349.846 |
| 8 | 08:00 | 1092.97 | 1.138510417 | 546.485000 | 5464850.000 | 4800000 | -27702.083 | 58593.750 | 4760349.846 | 4732647.763 |
| 9 | 09:00 | 1077.53 | 1.122427083 | 538.765000 | 5387650.000 | 4800000 | -24485.417 | 30891.667 | 4732647.763 | 4708162.346 |
| 10 | 10:00 | 1065.60 | 1.110000000 | 532.800000 | 5328000.000 | 4800000 | -22000.000 | 6406.250 | 4708162.346 | 4686162.346 |
| 11 | 11:00 | 1058.90 | 1.103020833 | 529.450000 | 5294500.000 | 4800000 | -20604.167 | -15593.750 | 4686162.346 | 4665558.179 |
| 12 | 12:00 | 1047.59 | 1.091239583 | 523.795000 | 5237950.000 | 4800000 | -18247.917 | -36197.917 | 4665558.179 | 4647310.263 |
| 13 | 13:00 | 1011.43 | 1.053572917 | 505.715000 | 5057150.000 | 4800000 | -10714.583 | -54445.833 | 4647310.263 | 4636595.679 |
| 14 | 14:00 | 1117.03 | 1.163572917 | 558.515000 | 5585150.000 | 4800000 | -32714.583 | -65160.417 | 4636595.679 | 4603881.096 |
| 15 | 15:00 | 1111.64 | 1.157958333 | 555.820000 | 5558200.000 | 4800000 | -31591.667 | -97875.000 | 4603881.096 | 4572289.429 |
| 16 | 16:00 | 942.02 | 0.981270833 | 471.010000 | 4710100.000 | 4800000 | 3745.833 | -129466.667 | 4572289.429 | 4576035.263 |
| 17 | 17:00 | 938.30 | 0.977395833 | 469.150000 | 4691500.000 | 4800000 | 4520.833 | -125720.833 | 4576035.263 | 4580556.096 |
| 18 | 18:00 | 932.94 | 0.971812500 | 466.470000 | 4664700.000 | 4800000 | 5637.500 | -121200.000 | 4580556.096 | 4586193.596 |
| 19 | 19:00 | 926.90 | 0.965520833 | 463.450000 | 4634500.000 | 4800000 | 6895.833 | -115562.500 | 4586193.596 | 4593089.429 |
| 20 | 20:00 | 831.28 | 0.865916667 | 415.640000 | 4156400.000 | 4800000 | 26816.667 | -108666.667 | 4593089.429 | 4619906.096 |
| 21 | 21:00 | 832.45 | 0.867135417 | 416.225000 | 4162250.000 | 4800000 | 26572.917 | -81850.000 | 4619906.096 | 4646479.013 |
| 22 | 22:00 | 828.74 | 0.863270833 | 414.370000 | 4143700.000 | 4800000 | 27345.833 | -55277.083 | 4646479.013 | 4673824.846 |
| 23 | 23:00 | 825.93 | 0.860343750 | 412.965000 | 4129650.000 | 4800000 | 27931.250 | -27931.250 | 4673824.846 | 4701756.096 |

β sum: 24; β mean: 1; Terminal V24: 4701756.096 m³; V24 - V0: 0.000 m³.

**S6E. DLFX hourly reconstruction and inventory trajectory**

βₖ = Table 5 hourly rate / 960; qout,ₖ = 530 × 10⁴βₖ m³/d; qin,ₖ = 530 × 10⁴ m³/d.

ΔVₖ = (qin,ₖ − qout,ₖ)/24; C₀ = 0; Cₖ₊₁ = Cₖ + ΔVₖ; Vₖ = selected preparation + Cₖ. The 24 rows include the 25 states V₀–V₂₄. Factors are displayed to nine decimals and inventory values to three decimals; calculation uses the unrounded worksheet formulas.

| **k** | **Hour** | **Table 5 rate (10⁴ m³/d)** | **Reconstructed βₖ** | **Segment rate (10⁴ m³/d)** | **qout,ₖ (m³/d)** | **qin,ₖ (m³/d)** | **ΔVₖ (m³)** | **Cₖ (m³)** | **Vₖ (m³)** | **Vₖ₊₁ (m³)** |
| --- | --- | --- | --- | --- | --- | --- | --- | --- | --- | --- |
| 0 | 00:00 | 846.84 | 0.882125000 | 467.526250 | 4675262.500 | 5300000 | 26030.729 | 0.000 | 1571469.794 | 1597500.523 |
| 1 | 01:00 | 858.41 | 0.894177083 | 473.913854 | 4739138.542 | 5300000 | 23369.227 | 26030.729 | 1597500.523 | 1620869.751 |
| 2 | 02:00 | 878.53 | 0.915135417 | 485.021771 | 4850217.708 | 5300000 | 18740.929 | 49399.957 | 1620869.751 | 1639610.680 |
| 3 | 03:00 | 898.95 | 0.936406250 | 496.295313 | 4962953.125 | 5300000 | 14043.620 | 68140.885 | 1639610.680 | 1653654.300 |
| 4 | 04:00 | 942.41 | 0.981677083 | 520.288854 | 5202888.542 | 5300000 | 4046.311 | 82184.505 | 1653654.300 | 1657700.610 |
| 5 | 05:00 | 932.59 | 0.971447917 | 514.867396 | 5148673.958 | 5300000 | 6305.252 | 86230.816 | 1657700.610 | 1664005.862 |
| 6 | 06:00 | 937.36 | 0.976416667 | 517.500833 | 5175008.333 | 5300000 | 5207.986 | 92536.068 | 1664005.862 | 1669213.848 |
| 7 | 07:00 | 1103.66 | 1.149645833 | 609.312292 | 6093122.917 | 5300000 | -33046.788 | 97744.054 | 1669213.848 | 1636167.060 |
| 8 | 08:00 | 1092.97 | 1.138510417 | 603.410521 | 6034105.208 | 5300000 | -30587.717 | 64697.266 | 1636167.060 | 1605579.343 |
| 9 | 09:00 | 1077.53 | 1.122427083 | 594.886354 | 5948863.542 | 5300000 | -27035.981 | 34109.549 | 1605579.343 | 1578543.362 |
| 10 | 10:00 | 1065.60 | 1.110000000 | 588.300000 | 5883000.000 | 5300000 | -24291.667 | 7073.568 | 1578543.362 | 1554251.695 |
| 11 | 11:00 | 1058.90 | 1.103020833 | 584.601042 | 5846010.417 | 5300000 | -22750.434 | -17218.099 | 1554251.695 | 1531501.261 |
| 12 | 12:00 | 1047.59 | 1.091239583 | 578.356979 | 5783569.792 | 5300000 | -20148.741 | -39968.533 | 1531501.261 | 1511352.520 |
| 13 | 13:00 | 1011.43 | 1.053572917 | 558.393646 | 5583936.458 | 5300000 | -11830.686 | -60117.274 | 1511352.520 | 1499521.834 |
| 14 | 14:00 | 1117.03 | 1.163572917 | 616.693646 | 6166936.458 | 5300000 | -36122.352 | -71947.960 | 1499521.834 | 1463399.482 |
| 15 | 15:00 | 1111.64 | 1.157958333 | 613.717917 | 6137179.167 | 5300000 | -34882.465 | -108070.312 | 1463399.482 | 1428517.017 |
| 16 | 16:00 | 942.02 | 0.981270833 | 520.073542 | 5200735.417 | 5300000 | 4136.024 | -142952.778 | 1428517.017 | 1432653.041 |
| 17 | 17:00 | 938.30 | 0.977395833 | 518.019792 | 5180197.917 | 5300000 | 4991.753 | -138816.753 | 1432653.041 | 1437644.794 |
| 18 | 18:00 | 932.94 | 0.971812500 | 515.060625 | 5150606.250 | 5300000 | 6224.740 | -133825.000 | 1437644.794 | 1443869.534 |
| 19 | 19:00 | 926.90 | 0.965520833 | 511.726042 | 5117260.417 | 5300000 | 7614.149 | -127600.260 | 1443869.534 | 1451483.683 |
| 20 | 20:00 | 831.28 | 0.865916667 | 458.935833 | 4589358.333 | 5300000 | 29610.069 | -119986.111 | 1451483.683 | 1481093.753 |
| 21 | 21:00 | 832.45 | 0.867135417 | 459.581771 | 4595817.708 | 5300000 | 29340.929 | -90376.042 | 1481093.753 | 1510434.681 |
| 22 | 22:00 | 828.74 | 0.863270833 | 457.533542 | 4575335.417 | 5300000 | 30194.358 | -61035.113 | 1510434.681 | 1540629.039 |
| 23 | 23:00 | 825.93 | 0.860343750 | 455.982187 | 4559821.875 | 5300000 | 30840.755 | -30840.755 | 1540629.039 | 1571469.794 |

β sum: 24; β mean: 1; Terminal V24: 1571469.794 m³; V24 - V0: 0.000 m³.

**S6F. Workbook consistency checks**

Results below are evaluated from the supplied workbook formulas. Numeric comparisons use the workbook tolerance of 10⁻⁶. These checks cover aggregation, normalization, end-of-day inventory closure, and selected-value membership in the supplied optimal intervals.

| **Check** | **Formula / basis** | **Result** | **Expected** | **Status** |
| --- | --- | --- | --- | --- |
| West raw aggregate | SUM station contributions | 4817675.298713333 | 4817675.298713333 | Pass |
| West rounded baseline | ROUND(raw/10⁴, 2) | 481.77 | 481.77 | Pass |
| West beta sum | SUM reconstructed beta | 24 | 24 | Pass |
| West beta mean | AVERAGE reconstructed beta | 1 | 1 | Pass |
| West V24-V0 | 24-interval conservation | 0 | 0 | Pass |
| LFFX selected in optimal interval | interval membership | Yes | Yes | Pass |
| NGXDD beta sum | SUM east beta | 24 | 24 | Pass |
| NGXDD V24-V0 | 24-interval conservation | 0 | 0 | Pass |
| NGXDD selected in optimal interval | interval membership | Yes | Yes | Pass |
| DLFX beta sum | SUM east beta | 24 | 24 | Pass |
| DLFX V24-V0 | 24-interval conservation | 0 | 0 | Pass |
| DLFX selected in optimal interval | interval membership | Yes | Yes | Pass |
