# Supplementary material for: A forecast-to-dispatch workflow for day-ahead operation of a regional natural gas transmission network based on station-level load prediction and linepack target setting
Source: PLoS One. 2026 Oct 5;21(10):e0349981. doi: 10.1371/journal.pone.0349981 (PMC13637901; doi:10.1371/journal.pone.0349981)
Supplement: Supplementary file 7 [file pone.0349981.s014.docx]

**Response to the Academic Editor and Reviewers**

**PONE-D-26-22124**

*A forecast-to-dispatch workflow for day-ahead operation of a regional natural gas transmission network based on station-level load prediction and linepack target setting*

**Overview of the revision.** The manuscript now presents the contribution as an engineering integration; defines the forecast-to-dispatch boundary; documents a leakage-controlled 30-origin evaluation; separates routine stations from data-quality boundary cases; strengthens baseline and PSO checks; narrows the linepack formulation to its reduced-order quasi-steady scope; and supplies separately uploaded tables, figures, code, and anonymized data. Unsupported seasonal and transient uncertainty values were removed rather than inferred.

**Journal requirements**

**Journal Requirement 1: PLOS ONE style and file naming**

**Comment:** Please ensure that your manuscript meets PLOS ONE's style requirements, including those for file naming.

**Response and revision made:** The manuscript has been reformatted as a single-column, double-spaced Word file with continuous line numbering and page numbers. The title is in sentence case, a short title has been added, the Methods heading has been standardized, the reference list has been converted to Vancouver style, and Supporting Information legends appear after the references. The submission package uses explicit PLOS upload labels and file names.

**Journal Requirement 2: ORCID**

**Comment:** PLOS requires an ORCID iD for the corresponding author in Editorial Manager on papers submitted after December 6th, 2016. Please ensure that you have an ORCID iD and that it is validated in Editorial Manager.

**Response and revision made:** The corresponding author will verify the ORCID iD through the Fetch/Validate link in Editorial Manager before completing resubmission. Coauthors have also been asked to verify their own ORCID records where available. This account-level action cannot be completed by a coauthor or by the manuscript-preparation software and is listed in the submission checklist.

**Journal Requirement 3: AI use statement**

**Comment:** We note you use an AI tool to assist with your submission. Please update your AI use statement to include the name(s) of any tools used, a description of how the authors used the tool(s), how you evaluated the validity of the tool's outputs, and a clear statement of which aspects of the study, article contents, data, or supporting files were affected or generated by AI tool usage.

**Response and revision made:** The Acknowledgments now name ChatGPT and Codex (OpenAI), describe their use for English editing and for organizing, reviewing, and documenting author-supplied analysis code, explain that the authors checked all outputs against project records and engineering context, and state that the tools did not create source observations or make scientific decisions. The authors accept responsibility for the manuscript, calculations, code, tables, figures, and supporting files.

**Journal Requirement 4: Code sharing**

**Comment:** Please note that PLOS ONE has specific guidelines on code sharing for submissions in which author-generated code underpins the findings in the manuscript. In these cases, we expect all author-generated code to be made available without restrictions upon publication of the work. Please review our guidelines and ensure that your code is shared in a way that follows best practice and facilitates reproducibility and reuse.

**Response and revision made:** S1 Code contains the author-generated scripts used for the released forecasting, preprocessing, ablation, bootstrap, model-parameter, PSO robustness, and plotting workflows. It includes fixed seeds, configuration files, pinned requirements, recorded outputs, smoke tests, and a README. Using the anonymized supporting inputs, the package reproduces the computational workflow and the main analysis procedures. It does not claim to reconstruct restricted absolute telemetry or a transient hydraulic simulator.

**Journal Requirement 5: Data availability**

**Comment:** All PLOS journals require all data underlying the findings described in the manuscript to be freely available to other researchers, either in a public repository, within the manuscript itself, or as Supporting Information. If the data cannot be made publicly available for ethical or legal reasons, please explain the reasons on resubmission so that an exemption request can be escalated for approval.

**Response and revision made:** Raw station telemetry, exact asset identifiers, detailed pressure and flow trajectories, pipeline configuration, and site-specific operating limits are owned by the data provider and are subject to commercial-confidentiality and pipeline-security restrictions. We request approval of this data-policy exemption. S1 Data releases the maximum non-sensitive material available to the authors: anonymized indexed daily station data, 30-day predictions and metrics, ablation and bootstrap outputs, PSO summaries, archived aggregate reconstruction and linepack-target results, a data dictionary, and a restriction statement. The revised Data Availability statement distinguishes recalculable public outputs from restricted archived aggregates.

**Journal Requirement 6: Author affiliations**

**Comment:** Please amend your list of authors on the manuscript to ensure that each author is linked to an affiliation. Authors' affiliations should reflect the institution where the work was done.

**Response and revision made:** The title page now links every author to a numbered affiliation: Ying Liu and Yuanyuan Tian to affiliation 1, Yong Xiao to affiliation 2, Zhiping Tang to affiliation 3, Simin Zhong to affiliation 4, and Chenguang Yong to affiliation 5. The corresponding author is identified separately with an e-mail address.

**Journal Requirement 7: Supporting tables**

**Comment:** We notice that your supplementary tables are included in the manuscript file. Please remove them and upload them with the file type 'Supporting Information'. Please ensure that each Supporting Information file has a legend listed in the manuscript after the references list.

**Response and revision made:** The supplementary tables have been removed from the main manuscript and prepared as five separate Supporting Information files (S1-S5 Tables). Their legends are listed after the references. S1-S4 Tables report station-level metrics, and S5 Table reports model-wise routine-station summaries and bootstrap intervals.

**Journal Requirement 8: Supporting figures**

**Comment:** We notice that your supplementary figures are uploaded with the file type 'Figure'. Please amend the file type to 'Supporting Information'. Please ensure that each Supporting Information file has a legend listed in the manuscript after the references list.

**Response and revision made:** S1-S4 Figs are supplied as separate Supporting Information files rather than main-figure files. Their legends are listed after the references, and the upload manifest identifies the required Supporting Information file type.

**Journal Requirement 9: Recommended references**

**Comment:** If the reviewer comments include a recommendation to cite specific previously published works, please review and evaluate these publications to determine whether they are relevant and should be cited. There is no requirement to cite these works unless the editor has indicated otherwise.

**Response and revision made:** We evaluated the recommended publications individually. A concise Introduction paragraph now cites the seven studies that are methodologically relevant to heterogeneous signal fusion, limited abnormal samples, lightweight interpretability, and prior-informed network design [21-27]. The revised text treats these studies as cross-domain methodological context rather than as direct natural-gas forecasting benchmarks.

**Reviewer #1**

**Reviewer #1 Comment 1: Nature of the contribution**

**Comment:** The main contribution of this paper lies in 'engineering integration' rather than 'algorithmic innovation.' CNN-BiLSTM-Attention, intraday coefficient reconstruction, and PSO optimization are all established technologies; the core value lies in integrating these three into a practical day-ahead scheduling workflow.

**Response and revision made:** We agree. The Abstract, Introduction, workflow description, Discussion, and Conclusions now identify the contribution as an engineering integration and evaluation of established components. Claims of algorithmic novelty have been removed. Fig 1 and Section 2.1 present the operational interface from daily station forecasts to hourly withdrawals and reduced-order segment targets.

**Reviewer #1 Comment 2: Intraday allocation coefficients**

**Comment:** The intraday allocation coefficients are based on historical average patterns and may fail during holidays, sudden changes in industrial load, or accident scenarios. Although this limitation is mentioned in the paper, no quantitative assessment is provided, such as whether reconstruction errors increase significantly during holidays. It is recommended to supplement the analysis with an examination of reconstruction errors on abnormal days or to discuss how to dynamically update the allocation coefficients.

**Response and revision made:** The manuscript now states that the coefficients were estimated from preceding training data only and should be updated on a rolling basis when recent complete hourly observations become available. It also specifies operator review for holidays, abrupt industrial-load changes, and emergency operation. We retained the traceable archived 18-day segment validation and did not report the earlier placeholder abnormal-day table because the archive does not preserve date-resolved abnormal-day reconstruction records. This limitation and the need for broader seasonal and abnormal-day validation are stated explicitly.

**Reviewer #1 Comment 3: Literature review**

**Comment:** The literature review lacks comprehensiveness in its introduction of relevant methodologies. It is recommended to cite the following works: Multimodal Heterogeneous Signal Fusion Detection (https://doi.org/10.1016/j.ymssp.2026.113923) and Zero-Sample Detection (https://doi.org/10.1080/10589759.2025.2534429; https://doi.org/10.1016/j.eswa.2025.128592), lightweight explainable detection (10.1088/1361-6501/acd5ef; https://doi.org/10.1007/s11071-024-10157-1; https://ieeexplore.ieee.org/abstract/document/10473026), physics-based prior neural network design (https://doi.org/10.1016/j.ress.2023.109793), etc.

**Response and revision made:** The Introduction has been expanded with a concise cross-domain paragraph covering heterogeneous signal fusion, limited or zero abnormal samples, lightweight interpretable networks, and prior-informed prediction [21-27]. The discussion connects these directions to heterogeneous station telemetry, data-quality boundaries, and interpretable engineering deployment without treating fault-diagnosis studies as direct gas-load benchmarks.

**Reviewer #1 Comment 4: Objective function**

**Comment:** The objective function minimizes only the mean absolute deviation between the actual pipeline storage and the target, without incorporating economic or safety factors such as compressor energy consumption, gas procurement costs, or penalties for pressure fluctuations. This may lead to oscillations near the boundary of the target pipeline. It is recommended to discuss the practical feasibility of the solutions obtained without economic objectives, or to indicate future directions for integrating economic scheduling.

**Response and revision made:** Sections 2.5 and 3.6 now define the calculation as reduced-order linepack target setting, not full economic dispatch. Pressure and linepack envelopes are enforced as feasibility conditions, and the resulting targets require operator review. The objective does not include compressor energy, procurement cost, or pressure-fluctuation penalties; this limitation is stated in the Results and Limitations, where integration with economic scheduling or MPC is identified as future work.

**Reviewer #1 Comment 5: Leakage control**

**Comment:** Although walk-forward validation was employed, were feature selection (MIC), PCA dimensionality reduction, and normalization parameters strictly fitted using only the training set in each iteration?

Response and revision made: Yes. Sections 2.2.3 and 2.6.1 and Fig 3 now state that imputation, normalization, nonlinear relevance screening, wavelet parameters, PCA loadings, and model parameters are estimated from the available historical model-fitting data at each origin. The fitted transformations are then applied to the chronological validation block and next-day input. The held-out next-day target is used only for scoring. For SARIMAX, known next-day calendar indicators and a one-day persistence forecast of temperature are used; observed test-day weather is not used. S1 Code records these boundaries.

**Reviewer #1 Comment 6: Site-specific model deployment**

**Comment:** Ablation experiments showed that the contributions of different components vary across sites (Attention-only performs better at stable sites, while CNN+Attention performs better at volatile sites), but this conclusion was not used to guide actual deployment—should different models be selected for different sites?

**Response and revision made:** We agree. The ablation interpretation now states that component utility is station dependent. The recalculated baseline comparison likewise shows that the best model differs by station. The deployment recommendation has therefore been revised from a single universal architecture to validation-based station-specific selection, with simpler models retained at regular stations when they perform competitively.

**Reviewer #1 Comment 7: Station G**

**Comment:** Including site G, which has a 56% data missing rate, in the evaluation and reporting a 91% MAPE—while valuable as a 'borderline case'—may mislead readers into believing the model performs poorly.

**Response and revision made:** Station G is now designated a pre-specified data-quality boundary case and is excluded from routine-performance averages, bootstrap inference, and ordinary-station conclusions. Its results are reported separately to show the limit imposed by severe telemetry incompleteness. Station K is excluded from the common 30-day evaluation because its usable history is insufficient. The Abstract, Tables 1 and 8-9, Results, and Conclusions use this separation consistently.

**Reviewer #1 Comment 8: Error metrics**

**Comment:** Reporting MAPE, MedianAPE, MAE, and RMSE simultaneously is problematic, as MAPE is prone to being inflated by outliers during low-load periods, whereas MedianAPE is more suitable for asymmetric distributions.

**Response and revision made:** The revised manuscript assigns distinct roles to the four metrics. MAPE remains the primary relative metric for comparability, MedianAPE is reported as the robust measure of typical percentage error, and MAE and RMSE retain physical-scale information and sensitivity to large errors. Station G is interpreted using both MAPE and MedianAPE and is not included in routine averages. The text no longer treats the metrics as interchangeable.

**Reviewer #1 Comment 9: Repetition and MIC interpretation**

**Comment:** There are multiple instances of text repetition (e.g., the title of Figure 9 is repeated nine times in a row), and the MIC matrix in Figure 10 is not clearly interpreted in the main text (it merely states 'MIC values are 0.91...' without labeling the variable names).

**Response and revision made:** The repeated Figure 9 caption text has been removed. The Fig 10 discussion now identifies the variables explicitly: daily load, average, minimum, and maximum temperature, air pressure, humidity, and calendar variables. It reports the strongest load associations and the redundancy among temperature variables, then links these findings to the relevance-screening and PCA steps.

**Reviewer #1 Comment 10: Quantitative comparison**

**Comment:** The introduction cites a large number of references on natural gas load forecasting and pipeline network scheduling, but the results and discussion section lacks quantitative comparisons with similar methods (e.g., direct use of LSTM with intraday rolling scheduling, or pipeline inventory optimization based on model predictive control).

**Response and revision made:** The forecasting comparison has been recalculated under one common 30-origin walk-forward protocol for SARIMAX, XGBoost, BPNN, LSTM, GRU, and CNN-BiLSTM-Attention. Complete station metrics are provided in S1-S4 Tables and the routine-station summary and paired bootstrap intervals in S5 Table. MPC was not used as a numerical benchmark because the archived target-setting formulation does not contain the closed-loop states and control actions required for a matched comparison. The manuscript now states this scope difference and avoids claiming equivalence to MPC dispatch.

**Reviewer #2**

**Reviewer #2 Comment 1: Workflow boundary**

**Comment:** The boundary of the proposed forecast-to-dispatch workflow should be clarified more explicitly. The manuscript emphasizes that the proposed method is not a fully integrated end-to-end control optimization framework, but rather a sequential workflow consisting of forecasting, hourly demand reconstruction, and linepack target setting. The authors should more clearly distinguish forecast outputs, reconstructed operational inputs, decision variables, and dispatch-support outputs. This clarification is necessary to avoid the impression that the forecasting model and the dispatch optimization module are only loosely connected.

**Response and revision made:** Fig 1 and Section 2.1 now label the daily station forecast as the forecast output, the reconstructed hourly nodal withdrawal profile as the operational input to target setting, the segment target linepack as the decision variable, and target linepack, average pressure, operating zone, and packing or drafting direction as dispatch-support outputs. The text states that the modules are sequentially coupled through these explicit quantities and that the workflow is not an end-to-end closed-loop controller.

**Reviewer #2 Comment 2: Missing data and anomalies**

**Comment:** More quantitative details should be provided for missing-data handling and anomaly treatment. The manuscript reports that Station G has a missing-data rate of 56.18%, but the missing-data rates and anomaly proportions of the other stations are not sufficiently documented. The authors should provide a station-wise summary of missing values, outliers, and imputation results. The parameters of the KNN imputation method should also be reported. In addition, the manuscript should explain whether Station G affects the overall performance statistics and why it is retained rather than excluded from the main evaluation.

Response and revision made: Table 1 reports the archived source-point missing rate and complete-day missing proportion for all 11 stations and now adds a reproducible aggregate-daily audit of outlier screening and imputation. Under the Q1 - 3IQR and Q3 + 3IQR rule, the released daily series contained one flagged record at Station D (0.19%) and none at the other stations; distance-weighted KNN with three neighbors filled all flagged entries, leaving zero residual missing values among archived daily rows. The table note distinguishes these aggregate-daily counters from the source-point telemetry summaries and from the split-specific training-only preprocessing used in evaluation. Station G remains a separately reported data-quality boundary case and is excluded from routine-performance inference; Station K is excluded from the common 30-day evaluation because only 62 daily observations were available.

**Reviewer #2 Comment 3: Leakage-control procedure**

**Comment:** The leakage-control procedure should be described in a more reproducible way. Although the manuscript states that walk-forward validation with leakage control was adopted, the exact implementation remains insufficiently detailed. The authors should provide a step-by-step workflow, table, or pseudo-code showing how the training, validation, and test windows are constructed. It should also be clearly stated at which stage KNN imputation, normalization, MIC-based feature screening, PCA, and wavelet denoising are fitted and applied. This is important because any preprocessing step using future information may lead to optimistic forecasting results.

Response and revision made: Sections 2.2.3 and 2.6.1 and Fig 3 specify a 14-day lookback, a fixed final 30-observation hold-out period, and one-day expanding-origin evaluation. At every origin, the most recent 10% of available historical observations, with a minimum of 14, forms the chronological validation block. Outlier thresholds, KNN imputation, normalization, nonlinear relevance screening, wavelet parameters, PCA, and model fitting use no next-day target information. The fitted transformations are applied to validation and next-day inputs. SARIMAX uses known calendar indicators and a one-day persistence temperature forecast, not observed test-day weather. S1 Code records the preprocessing boundary at every origin.

**Reviewer #2 Comment 4: Limited sample size and overfitting**

**Comment:** The suitability of the deep-learning architecture under a limited sample size should be further justified. Each station appears to contain only about 540 daily observations, whereas the proposed CNN-BiLSTM-Attention model is relatively complex. The authors should provide the number of trainable parameters, training and validation loss curves, early-stopping behavior, or additional evidence showing that overfitting is well controlled. This is particularly important for relatively regular stations, where simpler models may already perform competitively.

**Response and revision made:** The Methods now report 116,641 trainable parameters for the selected configuration. Dropout, chronological validation, and early stopping with a patience of 10 epochs were used. S3 Fig presents representative training and validation MSE histories for Stations A, F, and G. The revised Results also report that simpler baselines frequently match or outperform the hybrid model, and deployment guidance now favors station-specific validation rather than universal use of the deepest architecture.

**Reviewer #2 Comment 5: Baseline comparison**

**Comment:** The comparison with baseline models should be strengthened. The manuscript compares the proposed model with SARIMAX, XGBoost, BPNN, LSTM, and GRU models. However, the hyperparameter selection procedure for these baselines should be described more rigorously. The authors should clarify the search space, tuning criterion, and whether all models use the same validation protocol. Statistical significance tests, bootstrap confidence intervals, or error-distribution plots over the hold-out period would make the comparison more convincing than reporting only average MAPE, MAE, and RMSE values.

Response and revision made: Table 7 reports the fixed configurations used for all six model families and the chronological validation role. Candidate configurations were evaluated only within the historical development data and were frozen before the common 30-day hold-out evaluation; the public package does not claim an exhaustive search that was not preserved in the archive. All models use identical forecast origins and preprocessing boundaries. SARIMAX uses a one-day persistence forecast for next-day temperature and known calendar indicators, so observed test-day weather does not enter the comparison. S5 Table provides paired station-level bootstrap intervals across the nine routine stations, while S1-S4 Tables give the complete station-level error metrics. After this correction, SARIMAX achieved a mean station-level MAPE of 8.61% across the nine routine stations.

**Reviewer #2 Comment 6: Daily-to-hourly reconstruction**

**Comment:** The daily-to-hourly demand reconstruction method requires broader validation. The use of historical intraday allocation coefficients is a key interface between daily forecasting and linepack analysis. At present, the validation is mainly reported for three pipeline segments and 18 validation days. The authors should test the reconstruction rule across different seasons, weekdays, holidays, and abnormal load conditions. In addition, the operational impact of a 1-1.5 h peak-timing error should be analyzed, especially its effect on target linepack and average pressure.

**Response and revision made:** We retained the traceable archived 18-day validation for LFFX, NGXDD, and DLFX, including hourly MAPE, cumulative daily error, peak-timing error, and peak-load error. The manuscript does not report the earlier placeholder seasonal analysis because date-resolved reconstruction outputs were unavailable. It now states that broader seasonal and abnormal-day validation remains necessary. A quantitative transient pressure effect was also not reported because the archive lacks synchronized hourly boundary data and a calibrated transient model; the corresponding limitation replaces unsupported sensitivity claims.

**Reviewer #2 Comment 7: Physical constraints**

**Comment:** The physical constraints in the linepack optimization model should be described more completely. The current optimization model focuses on target linepack and average pressure, but the treatment of hydraulic feasibility remains relatively simplified. The authors should clarify whether the model is steady-state, quasi-steady-state, or transient. Important elements such as gas-flow equations, pressure-drop relationships, inlet supply constraints, valve operations, flow-direction changes, and nodal boundary conditions should be described in greater detail. Without these details, the dispatch feasibility of the optimized targets is difficult to fully assess.

**Response and revision made:** The formulation is now identified as reduced-order and quasi-steady, not transient. The Methods define the segment-average pressure and linepack relationships, prescribed inlet supply and forecast-derived withdrawals, pressure and linepack envelopes, fixed network configuration, and the representative-segment scope. The text also identifies excluded functions: transient nodal trajectories, valve switching, compressor dispatch, flow reversal, and time-varying nodal boundary optimization. Fig 13 and Table 6 are harmonized with the archived targets and search intervals, and field use is conditioned on operator review and, where available, transient-model verification.

**Reviewer #2 Comment 8: PSO configuration**

**Comment:** The PSO configuration appears too limited and needs further justification. The PSO algorithm uses only 20 particles and 10 iterations, which may be insufficient for reliable optimization, even if the decision space is relatively small. The authors should provide convergence curves, repeated-run statistics, and sensitivity tests for swarm size and iteration number. A comparison with grid search or another deterministic search method would also help verify that the reported target linepack values are not artifacts of a particular random optimization run.

**Response and revision made:** Each PSO budget was repeated 30 times with fixed documented seeds. Table 13 compares 20 particles x 10 iterations, 40 x 10, and 20 x 30 and reports target and objective stability against a deterministic grid over the same bounds. S4 Fig reports the mean best-so-far convergence trajectory and standard deviation for the reference setting. For LFFX, the archived reduced-order objective has an equivalent-minimum interval containing the reported 1.272 x 10^6 m3 target. Run-level outputs and the deterministic check are supplied in S1 Code and S1 Data.

**Reviewer #2 Comment 9: Uncertainty propagation**

**Comment:** Uncertainty propagation from forecasting errors to dispatch targets should be added. The manuscript currently reports point forecasts, point-based hourly reconstruction results, and point estimates of linepack and pressure targets. In practical dispatch, however, operators are more concerned with risk under forecast uncertainty. The authors should conduct scenario or sensitivity analysis, for example by perturbing daily demand forecasts by +/-5% and +/-10%, or shifting the peak hour by 1-2 h. The analysis should examine whether the optimized linepack and pressure targets remain within safe operating zones under these forecast-error scenarios.

**Response and revision made:** We agree that scenario-based uncertainty propagation is required before operational deployment. The available archive does not contain the synchronized date-resolved hydraulic boundaries and calibrated transient model needed to translate these perturbations into defensible pressure and flow trajectories. We therefore did not retain numerical placeholder scenarios. The contribution has been narrowed to point-based reduced-order day-ahead target setting, unsupported sensitivity values have been removed, and the Methods, Results, and Limitations identify forecast-scenario propagation with calibrated transient hydraulics as required future validation.
