# Supplementary material for: A forecast-to-dispatch workflow for day-ahead operation of a regional natural gas transmission network based on station-level load prediction and linepack target setting
Source: PLoS One. 2026 Oct 5;21(10):e0349981. doi: 10.1371/journal.pone.0349981 (PMC13637901; doi:10.1371/journal.pone.0349981)
Supplement: Supplementary file 8 [file pone.0349981.s015.docx]

Response to the Academic Editor and Reviewers

Manuscript ID: PONE-D-26-22124R1

A Forecast-to-Dispatch Workflow for Day-Ahead Operation of a Regional Natural Gas Transmission Network Based on Station-Level Load Prediction and Linepack Target Setting

We appreciate the opportunity to revise the manuscript. This response covers the Academic Editor and Journal Requirements, Reviewer 3 Comments 1-5, and Reviewer 4 Comments 1-7. The revisions clarify the operational evidence chain, units, input features, inventory state equation, and target-reporting convention. Remaining gaps in the archived records are stated explicitly. No forecasting models, full ablation experiments, or repeated-seed PSO studies were rerun in this final revision.

# Responses to the Academic Editor and Journal Requirements

## Editor

### Comment

Please revise

### Response

We have revised the manuscript to distinguish the common forecasting benchmark, the independent archived hourly-reconstruction evidence, and the retrospective engineering cases. The revision makes the hourly-rate units, fixed forecasting inputs, intertemporal inventory equation, and historical target-reporting rule explicit. We also added the recommended predict-and-optimize reference and consolidated the language and supporting-file descriptions.

The West-pipeline example is a retrospective reconstruction based on author-confirmed historical inputs and retained engineering records. Its correspondence to the predictions in the common 30-day benchmark remains unverified. The manuscript also reports the independently verified NGXDD inventory target, the separate limitation on replaying its archived pressure conversion, and the unavailable preprocessing records. The unverifiable archived cumulative-error column has been removed from Table 10.

We ask the Editor to consider the narrowed evidential scope and the explicitly identified archival gaps when assessing this revision.

### Changes in the manuscript

Sections 2.2-2.6, 3.2-3.6, Engineering implications and limitations, Conclusions, Tables 1, 2, 10, 12-14, and S6 Table.

## Submission files

### Comment

A letter that responds to each point raised by the academic editor and reviewer(s). You should upload this letter as a separate file labeled 'Response to Reviewers'.
A marked-up copy of your manuscript that highlights changes made to the original version. You should upload this as a separate file labeled 'Revised Manuscript with Track Changes'.
An unmarked version of your revised paper without tracked changes. You should upload this as a separate file labeled 'Manuscript'.
If you would like to make changes to your financial disclosure, please include your updated statement in your cover letter. Guidelines for resubmitting your figure files are available below the reviewer comments at the end of this letter.

### Response

The resubmission includes a clean revised manuscript, a cumulative marked-up manuscript showing the revisions from the version reviewed, this point-by-point response, and the updated Supporting Information files. The funding and competing-interest statements remain unchanged.

### Changes in the manuscript

Clean revised manuscript; cumulative marked-up manuscript; complete point-by-point response; updated Supporting Information.

## Journal Requirement 1

### Comment

If the reviewer comments include a recommendation to cite specific previously published works, please review and evaluate these publications to determine whether they are relevant and should be cited. There is no requirement to cite these works unless the editor has indicated otherwise.

### Response

We evaluated the recommended Chen et al. study for its relevance to the forecast-to-dispatch interface. It presents closed-loop, cost-oriented prediction for network-constrained unit commitment. We added it to the Introduction as reference [21] and distinguish its economic training objective from the sequential forecasting and inventory-deviation objective used here.

### Changes in the manuscript

Introduction, paragraph beginning "Closed-loop predict-and-optimize research"; reference [21].

### Revised text

Closed-loop predict-and-optimize research links forecasting to the downstream decision objective. Chen et al. trained a cost-oriented renewable-generation predictor using the objective and constraints of network-constrained unit commitment [21]. Their work distinguishes predictive accuracy from operating cost. The present gas-network workflow uses sequential forecasting and inventory preparation with a linepack-deviation objective.

[21] Chen X, Yang Y, Liu Y, Wu L. Feature-driven economic improvement for network-constrained unit commitment: A closed-loop predict-and-optimize framework. IEEE Transactions on Power Systems. 2022;37(4):3104-3118. doi:10.1109/TPWRS.2021.3128485.

## Journal Requirement 2

### Comment

Please review your reference list to ensure that it is complete and correct. If you have cited papers that have been retracted, please include the rationale for doing so in the manuscript text, or remove these references and replace them with relevant current references. Any changes to the reference list should be mentioned in the rebuttal letter that accompanies your revised manuscript. If you need to cite a retracted article, indicate the article’s retracted status in the References list and also include a citation and full reference for the retraction notice.

### Response

We checked the correspondence between numbered in-text citations and bibliography entries, added Chen et al. as reference [21], and renumbered subsequent citations. Six uncited entries remaining from earlier versions were removed: ElMahdy et al. (2025), Carpenter (2024), Liu et al. (2020), Cafaro et al. (2022), Guzzo et al. (2024), and Vidas et al. (2022). The resulting list contains 30 cited entries.

We checked the 30 retained references against Crossref bibliographic metadata and verified the new Chen citation against the institutional publication record. The metadata check corrected reference [4] to the 2026 volume 6, issue 1 record and removed an extra word from the title of reference [11]. No retraction or withdrawal update was flagged in the returned Crossref records. This check is limited to the available metadata and does not constitute exhaustive publisher-by-publisher clearance.

### Changes in the manuscript

Introduction and References; numbering throughout the manuscript.

### Revised text

[21] Chen X, Yang Y, Liu Y, Wu L. Feature-driven economic improvement for network-constrained unit commitment: A closed-loop predict-and-optimize framework. IEEE Transactions on Power Systems. 2022;37(4):3104-3118. doi:10.1109/TPWRS.2021.3128485.

# Responses to Reviewer 3

## Comment 1

### Comment

The forecasting evaluation uses a 30-day walk-forward test, whereas the temporal-disaggregation validation is based on a separate archived 18-day dataset. It remains unclear whether the reported linepack targets are generated from the same forecast origins evaluated in the forecasting section. At least one fully traceable example from station forecast → hourly reconstruction → segment demand → linepack target should be provided.

### Response

We cannot establish from the retained records that the operating targets in Table 12 were generated from the exact forecast outputs evaluated in Section 3.3. The 30-day forecasting benchmark and 18-day hourly-reconstruction archive are separate evaluations. We have preserved that distinction and added a retrospective West-pipeline example for 22 April 2023 in Section 3.6, Table 14, and S6 Table.

The authors confirmed the case date, the end-of-day forecast origin of 21 April 2023, the five historical station forecast inputs, and the participation rule. The weighted sum is 4,817,675.298713333 m³/d. The archived engineering implementation rounded this to 481.77 × 10⁴ m³/d before hourly disaggregation. Multiplication by the hourly factors used in the reconstruction reproduces all 24 Table 4 withdrawal rates at the displayed precision. With the archived supply and safe-envelope calibration, the inventory recurrence gives 25 boundary states. The selected LFFX preparation value of 1,271,536.975841198 m³ lies in the continuous optimal interval and attains the deterministic minimum.

The example provides a retrospective numerical chain conditional on author-confirmed historical inputs; their correspondence to the current common benchmark remains unverified. Station K contributes to this historical engineering input but remains excluded from the shared performance comparison.

### Changes in the manuscript

Sections 2.1, 2.4.3, 3.6; Table 14; S6 Table; Engineering implications and limitations.

### Revised text

The West-pipeline case for 22 April 2023 was reconstructed from historical station-demand inputs, participation coefficients, and engineering calculation records. The forecast origin was the end of 21 April 2023. Table 14 shows the calculation sequence for the 22 April 2023 West-pipeline case. S6 Table shows the input sources and full-precision numerical trace. Whether these historical inputs match predictions from the current common 30-day benchmark remains unverified.

The weighted station forecasts gave an unrounded daily aggregate of 4,817,675.298713333 m³/d, or 481.7675298713333 × 10⁴ m³/d. The engineering implementation rounded this aggregate to two decimal places in units of 10⁴ m³/d, giving a baseline of 481.77 × 10⁴ m³/d. Multiplication by the 24 full-precision hourly unevenness factors reproduced the withdrawal rates in Table 4 at the reported precision.

The median-set calculation yielded a continuous optimal interval of approximately 1,269,828.333–1,289,124.243 m³. The PSO-selected preparation value, 1,271,536.975841198 m³, lay within this feasible interval and attained the minimum mean absolute deviation of 64,168.628 m³.

## Comment 2

### Comment

The manuscript should explain more explicitly how source-point missingness is propagated through hourly imputation, station aggregation, and daily-load construction, and report station-level completeness before and after imputation.

### Response

Thank you for this comment. We revised Sections 2.2.1–2.2.3 and Table 1 to clarify the missing-data pathway from the received source-point archive to the station-level daily modeling series.

The revised manuscript distinguishes the data owner’s routine operational quality control from the research-side preprocessing performed after data receipt. Remaining source-point gaps were handled at hourly resolution, hourly flow rates were converted to daily mean rates, source points assigned to the same station were aggregated spatially, and remaining complete-day gaps were handled at daily resolution.

Table 1 now reports the available received-archive source-point missingness summaries by station and a separate audit of the released station-level daily data. The released daily audit contained no residual missing cells after research-side processing.

Exact station-level completeness counts before and after the original hourly-to-daily preprocessing could not be reconstructed because the record-level preprocessing counters were not retained. We have stated this limitation explicitly and report the retained missingness summaries and released-daily audit instead of inferring unsupported counts.

### Revision made

Section 2.2.1: clarified data-owner quality control before data delivery.

Section 2.2.1 and the text adjacent to Table 1: clarified received-archive missingness, aggregation, denominators, and the separate released-daily-data audit.

Section 2.2.3: clarified hourly treatment, daily averaging, station aggregation, and daily gap treatment. The unavailable original before/after counters are stated once in Section 2.2.1.

### Revised text

Table 1 shows received-archive source-point missingness and the separate audit of the released station-level daily series. The missingness summaries cover records received after data-owner quality control and before research-side imputation. Station missing rates are unweighted means of the constituent source-point rates. Complete-day-gap shares are missing-rate-weighted means of the source-point missing-type proportions, with total missingness as the denominator, not all calendar days.

In the daily-series audit, outlier percentages use the retained daily-row count N as the denominator. The final column reports N, daily-load values imputed after outlier screening, and residual missing cells across eight source/derived columns. These columns are load, three temperatures, month, weekday, weekend, and holiday. No residual missing cells remained. Record-level counters for the original hourly-to-daily preprocessing, including station-level completeness before and after processing, were not retained.

## Comment 3

### Comment

The authors should either implement station-specific model selection in the actual forecast-to-dispatch workflow or clearly justify why the hybrid model is retained for downstream target setting.

### Response

We follow the second option in the comment: the manuscript now explains why the historical forecasting inputs are retained. CNN-BiLSTM-Attention is the focal architecture for the architectural and ablation analysis and formed the original engineering forecasting workflow. Retaining the historical station forecasts preserves the provenance of the retrospective West-pipeline calculation; substituting models selected after the revised benchmark would define a different case.

The benchmark does not support imposing the hybrid model at every station. We recommend station-specific model selection using chronological validation for prospective deployment. This recommendation has not been implemented retrospectively, and the 30-day hold-out outcomes were not used to select new deployed models or regenerate the historical inputs. No forecasting model was retrained during this final revision.

### Changes in the manuscript

Sections 2.3, 2.6.2, 3.3.2, and 3.5; Conclusions.

### Revised text

CNN-BiLSTM-Attention is the focal hybrid architecture because it formed the original engineering forecasting workflow and supports the architectural ablation analysis. The historical hybrid forecasts were retained to reconstruct the original engineering case.

For prospective deployment, candidate models should be compared by chronological validation within each station. The selected model would then generate that station's day-ahead forecast.

The best-performing model differed by station. SARIMAX and XGBoost were competitive at several regular and volatile stations, while recurrent and hybrid models performed well at other sites. These results support station-specific model selection for prospective deployment, with CNN-BiLSTM-Attention considered alongside the other candidate models.

## Comment 4

### Comment

A more fundamental issue exists in Eq. (22). The equation is written as an instantaneous flow/mass-balance constraint but contains no intertemporal linepack accumulation term. Therefore, it is unclear how Eq. (22) can “generate the 24-h linepack trajectory” as stated in Section 2.5.3. A state-transition relation such as V(k+1) = V(k) + Δt × (inflow − outflow), or an equivalent inventory equation, appears necessary.

### Response

We agree that the previous instantaneous balance did not define an inventory trajectory. In the revised manuscript, Eq. (28) gives the intertemporal state transition and its initial condition. The decision variable is the initial preparation inventory xₑ, with Vₑ,₀(xₑ) = xₑ. Each step adds Δt times the prescribed inlet-minus-withdrawal rate; both rates are in standard m³/d and Δt is 1/24 day.

The 24 hourly intervals generate 25 boundary states, including Vₑ,₂₄. The objective in Eq. (25) retains the 24 interval-start deviations from the hourly reference trajectory. The terminal state is checked for conservation and envelope compliance but is not inserted as an extra objective term. Eqs. (29) and (30) give the cumulative-state representation, and Section 2.5.3 gives the resulting median-set characterization. The NGXDD inventory calculation and all 25 boundary states have been independently verified under its retained segment-specific inputs.

### Changes in the manuscript

Section 2.5.2, Eqs. (28)-(30), and Section 2.5.3.

### Revised text

The objective averages the absolute deviations of the 24 interval-start states Vₑ,₀(xₑ),...,Vₑ,₂₃(xₑ) from their hourly references. The terminal state Vₑ,₂₄(xₑ) is included in the end-of-day conservation and envelope-feasibility checks, but not in the 24-term objective.

where Qₑ,ₖⁱⁿ and Qₑ,ₖᵒᵘᵗ are the prescribed inlet-supply and aggregate withdrawal rates, standard m³/d; and Δt = 1/24 day is the time step.

The horizon contains *Nₕ* = 24 hourly intervals and 25 boundary states. *Vₑ,ₖ* is the segment linepack at boundary *k* = 0,...,*Nₕ*, expressed as standard-condition gas volume, m³.

Eq. (23) relates this inventory to segment-average pressure:

## Comment 5

### Comment

The operating targets reported in Table 12 are not exactly the same as the reference PSO or deterministic results in Table 13. For example, LFFX is reported as 1.272 × 10⁶ m³ in Table 12, whereas the mean PSO target is approximately 1.280 × 10⁶ m³. Although the former lies within an equivalent-minimum interval, the manuscript should specify a unique target-selection rule and consistently use the selected values in the Abstract, figures, tables, and conclusions.

### Response

The reporting rule now distinguishes the mathematical minimizing set, the single historical value reported for a case, and repeated-run solver statistics. The retained PSO selection is used as the operating target only when its inventory feasibility and optimal-set membership can be verified under the documented case inputs. Solver means and sampled-grid points remain diagnostics and do not replace that selection.

For LFFX, the archived value is 1,271,536.975841198 m³; the continuous optimal interval is approximately 1,269,828.333-1,289,124.243 m³ and the minimum mean absolute deviation is 64,168.628 m³. Its objective gap is zero. The two sampled-grid minima, 1.2712 and 1.2812 × 10⁶ m³, are distinguished from the complete continuous interval. For DLFX, the retained selection is 1,571,469.794304801 m³ within 1,559,382.189-1,573,735.267 m³, with a minimum mean absolute deviation of 71,567.257 m³ and zero gap. Both cases pass all-25-state inventory and associated pressure-envelope checks. Their displayed operating values are unchanged.

For NGXDD, the previously reported 480/530 discrepancy was resolved by restoring the correct segment-specific daily withdrawal baseline of 480 × 10⁴ m³/d. Both the inlet-supply and withdrawal baselines are therefore 480 × 10⁴ m³/d, and hourly withdrawals use the retained hourly unevenness factors. Independent deterministic verification places the archived selected linepack value of 4,701,756.095919851 m³ within the continuous minimizing interval of 4,695,278.411–4,707,161.027 m³. It attains the minimum mean absolute deviation of 64,746.628 m³, with zero objective gap, and the complete 25-state inventory trajectory satisfies the retained inventory constraints. Table 12 consequently retains 4.702 × 10⁶ m³ as the selected operating target. The reported preparation pressure of 4.86 MPa is retained from the archived engineering record because the matching pressure-conversion worksheet was not preserved and was unavailable for independent replay. This pressure-specific limitation does not invalidate the independent inventory verification.

Table 13 reports solver-returned values and PSO and sampled-grid diagnostics based on retained objective-support data; its numerical values are unchanged and do not replace the historical selected operating targets in Table 12. The Abstract, Section 3.6, Table 12, and Conclusions consistently report independently verified linepack preparation values for all three segments. The text adjacent to Table 12 and the Fig 12 caption identify the NGXDD pressure as an archived reported value. S6 Table records the NGXDD 480 baseline, hourly withdrawal reconstruction, continuous minimizing interval, minimum mean absolute deviation, and V₀–V₂₄ inventory checks, with the unavailable pressure-conversion worksheet identified separately.

### Changes in the manuscript

Section 2.5.3 reporting rule; Section 3.6; Fig 12 caption; Table 12 and its adjacent text; Table 13 and its adjacent text (numerical values unchanged); Section 4; Abstract and Conclusions; S6 Table.

### Revised text

For each historical case, the archived PSO-selected preparation value is reported as the operating target after confirming inventory feasibility and optimal-set membership. When several minimizers exist, the archived selected member fixes the reported operating value.

Table 12 and Fig 12 show the selected preparation values for the three historical engineering cases. The LFFX, NGXDD, and DLFX linepack targets were 1.272, 4.702, and 1.571 × 10⁶ m³, respectively, and passed case-specific deterministic inventory checks. These targets imply packing for LFFX and drafting for NGXDD and DLFX. The NGXDD pressure of 4.86 MPa is an archived reported value; its conversion could not be independently reproduced from the available records.

Table 13 shows the PSO results for the three computational budgets and the deterministic grid references. PSO results are reported as mean ± standard deviation over 30 runs. Table 12 shows the selected operating targets separately from these solver statistics. Under the 20-particle, 10-iteration setting, mean differences from the sampled-grid objective minima were 0.000%, 0.172%, and 0.008% for LFFX, NGXDD, and DLFX, respectively. The two optimal LFFX grid samples, 1.2712 and 1.2812 × 10⁶ m³, differ from the full continuous minimizing interval of approximately 1.269828333–1.289124243 × 10⁶ m³.

For NGXDD, inlet-supply and daily withdrawal baselines were both 480 × 10⁴ m³/d. The hourly withdrawal profile was reconstructed using the historical unevenness factors. The selected preparation value of 4,701,756.095919851 m³ lay within the continuous minimizing interval of 4,695,278.411–4,707,161.027 m³. It attained the minimum mean absolute deviation of 64,746.628 m³. All 25 inventory states satisfied the inventory constraints, supporting the reported preparation value of 4.702 × 10⁶ m³.

# Responses to Reviewer 4

## Comment 1

### Comment

I could not determine whether the operating targets in Table 12 were actually calculated from the out-of-sample forecasts reported in Section 3.3. The forecasting assessment uses a 30-day hold-out period, while the temporal reconstruction uses a separate archived 18-day case, and the connection between these records is not shown.

### Response

The available archive does not establish that Table 12 was calculated from the exact out-of-sample predictions reported in Section 3.3. The manuscript now states the distinct roles of the 30-day benchmark, the independent archived 18-day reconstruction evidence, and the historical engineering records. It does not present them as a single common-benchmark end-to-end validation.

Section 3.6, Table 14, and S6 Table reconstruct the West-pipeline case for 22 April 2023 using the authors' confirmed historical station inputs and participation rule. The five station forecasts aggregate to 4,817,675.298713333 m³/d, which the archived implementation rounded to 481.77 × 10⁴ m³/d. Multiplication by the high-precision hourly factors used in the reconstruction gives 24 LFFX withdrawal rates. The calculation then propagates 25 inventory boundary states and verifies the selected preparation value and pressure conversion. The historical Station K forecast contributes to this case only and is excluded from the shared model-performance statistics.

The example is a retrospective reconstruction with documented numerical conventions; its correspondence to the current benchmark forecasts remains unverified. The eastern cases remain separate records, and the NGXDD inventory target is independently verified while its archived pressure-conversion limitation is reported separately.

### Changes in the manuscript

Sections 2.1, 2.4.3, 3.6; Tables 12 and 14; Fig 12 caption; S6 Table; limitations.

### Revised text

The West-pipeline case for 22 April 2023 was reconstructed from historical station-demand inputs, participation coefficients, and engineering calculation records. The forecast origin was the end of 21 April 2023. Table 14 shows the calculation sequence for the 22 April 2023 West-pipeline case. S6 Table shows the input sources and full-precision numerical trace. Whether these historical inputs match predictions from the current common 30-day benchmark remains unverified.

The daily mean demand forecasts were 4,097,629.44125, 13,405.20833, 84,157.875, 205,933.9583, and 833,097.631666666 m³/d for Stations J, E, K, H, and I, respectively. Their West-pipeline participation coefficients were 1, 1, 1, 1, and 0.5. The station-level forecasts were first aggregated using this participation rule, after which the aggregated daily demand was temporally disaggregated into the 24-h LFFX withdrawal profile.

The weighted station forecasts gave an unrounded daily aggregate of 4,817,675.298713333 m³/d, or 481.7675298713333 × 10⁴ m³/d. The engineering implementation rounded this aggregate to two decimal places in units of 10⁴ m³/d, giving a baseline of 481.77 × 10⁴ m³/d. Multiplication by the 24 full-precision hourly unevenness factors reproduced the withdrawal rates in Table 4 at the reported precision.

## Comment 2

### Comment

The intraday coefficients are not defined consistently. Equations 8 and 9 describe them as shares whose total equals one, but the values in Tables 4 and 5 are centered around one.

### Response

The revised text defines the coefficients as dimensionless hourly unevenness factors with mean 1 across the 24 hourly intervals, equivalent to a sum of 24. Each hourly flow rate is the daily mean demand rate multiplied by its factor. Hourly interval volume is obtained by multiplication by 1/24 day. Eqs. (11)-(14) and the text introducing Tables 4 and 5 use this definition consistently; the archived hourly values are unchanged.

The West-pipeline example records the additional historical numerical convention: the aggregated daily forecast was rounded to two decimals in units of 10⁴ m³/d before multiplication by the high-precision hourly factors used in the reconstruction. S6 Table gives those factors and reproduces all 24 Table 4 values.

With the observed daily-mean baseline and mean-one factors, Eq. (19) provides the daily-volume conservation diagnostic. The date-level inputs and calculation records underlying the archived cumulative-error values of 2.2%, 1.9%, and 2.5% were not retained, and these values cannot be reconciled with the documented conservation relation. We therefore removed that column and its three entries from Table 10 and removed the associated cumulative-error statements from the main results. The entries were not replaced with zero or relabeled as another metric. Hourly MAPE, peak-timing error, and peak-demand magnitude error remain unchanged. The April intermediate rounding is not offered as an explanation for these percent-level values.

### Changes in the manuscript

Sections 2.4.1 and 2.4.3; Tables 4, 5, and 10; Section 3.6; S6 Table.

### Revised text

where $\hat{q}_{s,d+1,k}$ is the reconstructed station hourly flow rate, m³/d; $\hat{Q}_{s,d+1}$ is the predicted daily mean demand rate, m³/d; and $\beta_{s,k,c}$ is the dimensionless hourly unevenness factor for station *s*, hour *k*, and day type *c*. The volume assigned to interval *k* is:

$$\Delta V_{s,d+1,k}=\hat{q}_{s,d+1,k}\times\left(\frac{1}{24} \text{day}\right)$$

where Δt = 1/24 day, and $\Delta V_{s,d+1,k}$ is the gas volume assigned to interval *k*, m³.

Because the 24 hourly unevenness factors have a mean value of 1:

$$\frac{1}{24}\sum_{k=1}^{24} \hat{q}_{s,d+1,k}=\hat{Q}_{s,d+1}$$

Temporal disaggregation therefore preserves the predicted daily mean demand rate. Equivalently, the sum of the 24 hourly interval volumes equals the predicted daily gas volume.

With the observed daily-mean baseline in Eq. (17), the mean-one condition in Eq. (12), and Δt = 1/24 day, the reconstructed daily volume equals the observed daily volume apart from numerical rounding.

Forecast and reconstruction errors were evaluated separately, without quantifying their propagation into transient pressure and flow responses. The effects of the observed 1.0-1.5 h peak-timing errors on preparation linepack and average-pressure targets were not quantified. Broader multi-day and multi-season end-to-end validation requires synchronized forecast, withdrawal, supply, and linepack-state records. At data-impaired sites such as Station G, telemetry repair remains a priority for reliable forecasting.

## Comment 3

### Comment

The construction of daily station demand also needs clarification. Section 2.2.2 says that hourly instantaneous flows measured in cubic metres per day were summed to obtain daily load, but a flow rate normally must be integrated over the sampling interval.

### Response

Section 2.2.2 now distinguishes time aggregation from spatial aggregation. The hourly SSLL records are standard-volume flow rates in m³/d. Their complete-day arithmetic mean is the daily mean rate; summing the 24 hourly rates multiplied by 1/24 day gives daily volume in m³. Daily source-point mean rates are then summed spatially across the source points assigned to each station.

The daily mean rate and one-day volume have equal numerical values for a complete one-day interval but different units and physical meanings. The temporal-disaggregation and linepack sections use the same rate basis and explicit time-step conversion. This corrects the description and unit chain; it does not alter the archived Table 4 schedule.

### Changes in the manuscript

Section 2.2.2, Eqs. (1)-(3), Table 2, and Section 2.4.1.

### Revised text

The source telemetry contained source identifiers, timestamps, and instantaneous flow-rate records. The operational SSLL records are hourly standard-volume flow rates, m³/d. For a complete day with 24 equally spaced observations, the daily mean flow rate at source point p was calculated as:

$$\bar{Q}_{p,d}=\frac{1}{24}\sum_{h=1}^{24} q_{p,d,h}$$

where *q* is the hourly flow rate at source point *p* on day *d* at hour *h*, m³/d.

The corresponding daily gas volume is:

$$D_{p,d}=\sum_{h=1}^{24} q_{p,d,h}\times\left(\frac{1}{24} \text{day}\right)$$

where Δ*t* = 1/24 day. The daily mean rate is expressed in m³/d and the daily volume in m³.

Source-point daily mean flow rates assigned to the same station were then summed to obtain its daily mean demand rate:

$$Q_{s,d}=\sum_{p\in P_{s}} \bar{Q}_{p,d}$$

where *P*_s_ is the set of source points assigned to station *s*.

## Comment 4

### Comment

If inlet supply and withdrawals already determine the linepack trajectory, selecting a constant target that minimizes absolute deviations is essentially a one-dimensional median problem.

### Response

We agree. The initial preparation inventory is the scalar decision variable; the prescribed supply and withdrawal rates determine cumulative increments Cₑ,ₖ. A candidate therefore generates Vₑ,ₖ(xₑ) = xₑ + Cₑ,ₖ. The hourly reference Rₑ,ₖ is the midpoint of the prescribed safe envelope. Substitution into the archived mean absolute-deviation objective gives the median problem for the 24 transformed references Rₑ,ₖ - Cₑ,ₖ.

Section 2.5.3 states the continuous median interval and its intersection with the feasible candidate interval; if there is no intersection, the nearest feasible boundary minimizes the objective. PSO is retained as the archived numerical implementation, with repeated-run statistics used only as solver diagnostics. The selected historical value is distinguished from a unique mathematical minimizer. The terminal state is checked under the constraints but is excluded from the archived 24-term objective.

### Changes in the manuscript

Sections 2.5.2 and 2.5.3; Eq. (25) and Eqs. (29)-(32); Section 3.6 and Tables 12 and 13.

### Revised text

The objective averages the absolute deviations of the 24 interval-start states Vₑ,₀(xₑ),...,Vₑ,₂₃(xₑ) from their hourly references. The terminal state Vₑ,₂₄(xₑ) is included in the end-of-day conservation and envelope-feasibility checks, but not in the 24-term objective.

With prescribed inlet-supply and withdrawal profiles, the one-dimensional L1 objective has a deterministic median-set solution.

Let rₑ,ₖ = Rₑ,ₖ - Cₑ,ₖ, with the 24 values ordered from rₑ,₍₁₎ to rₑ,₍₂₄₎. The unconstrained minimizing set is:

$$\mathop{\mathrm{argmin}}\limits_{x_{e}} J_{e}(x_{e})=\left[r_{e,(12)},r_{e,(13)}\right]$$

If the median set intersects the feasible candidate interval implied by Eqs. (26) and (27), that intersection is optimal. Otherwise, the nearest feasible boundary minimizes the objective. PSO was the historical numerical implementation and was compared with this deterministic reference.

For each historical case, the archived PSO-selected preparation value is reported as the operating target after confirming inventory feasibility and optimal-set membership. When several minimizers exist, the archived selected member fixes the reported operating value.

## Comment 5

### Comment

I think the manuscript should distinguish more carefully between compliance with prescribed envelopes and hydraulic feasibility.

### Response

The manuscript now uses segment-level envelope compliance to describe the reduced-order checks. The model propagates a lumped inventory state and converts it to an associated average-pressure reference under the retained equivalent-pipe assumptions. It does not solve transient nodal pressure redistribution, flow reversal, valve actions, compressor dispatch, or the control trajectory required to establish the initial preparation inventory.

The reported quantities are inventory-preparation and average-pressure references for operator review. Section 4 describes the transient hydraulic verification required before operational implementation. Section 3.6 identifies the independently verified NGXDD linepack preparation value and the archived reported pressure of 4.86 MPa; the matching pressure-conversion worksheet was unavailable. The Fig 12 caption labels the NGXDD pressure as an archived reported value. Its appearance within a plotted band is not presented as proof of transient hydraulic feasibility.

### Changes in the manuscript

Sections 2.5.2, 3.6, and 4; Conclusions; the text adjacent to Table 12; Fig 12 caption.

### Revised text

The preparation value is restricted to the interior allowable band. Every candidate-generated state is checked against the segment-level linepack and associated average-pressure envelopes.

Inlet supply is prescribed, and segment withdrawals follow the temporal-disaggregation and station-to-node mapping procedures in Section 2.4. The engineering cases use their historical hourly withdrawal inputs, with network topology, valve and compressor status, and nominal flow direction fixed.

Fig 12. Observed and selected preparation points. (A) West pipeline-LFFX. (B) East pipeline-NGXDD. (C) East pipeline-DLFX. Colored bands show the prescribed linepack-pressure envelopes. NGXDD pressure: archived reported value.

The selected linepack is a segment-level preparation reference for the start of the next-day horizon. The reduced-order calculation checks inventory and average-pressure envelopes under fixed boundary conditions. It omits actuator sequences, valve switching, compressor dispatch, flow reversal, nodal pressure redistribution, and transient dynamics. Compressor fuel, procurement cost, and pressure-fluctuation penalties are outside the objective. Operational implementation requires current boundary data, operator review, and transient hydraulic verification. MPC was not benchmarked because the formulation lacks the closed-loop dynamic states and control actions needed for a matched comparison.

## Comment 6

### Comment

The feature-selection results are not sufficiently reproducible from the manuscript. Fig 10 does not identify the station, forecast origin, or training interval, and it contains pressure and humidity variables that are absent from Table 2. Please reconcile the predictor lists and show how the final eight inputs were obtained across stations and forecast origins.

### Response

We removed the earlier MIC figure because its retained matrix did not identify the station, forecast origin, or fitting interval. The former Figs 11-13 are renumbered as Figs 10-12, with corresponding updates to captions and references.

The revised text describes the fixed eight-dimensional input schema used in the implementation. It contains denoised daily load, temperature PC1, sine/cosine encodings for day of week and month, and weekend and holiday indicators. At each origin, the temperature scaling, PCA, and other fitted transformations use the chronological model-fitting subset. Archived preprocessing records support one temperature component meeting the 95% explained-variance criterion. The schema stays fixed across stations and origins; MIC scores do not select columns at individual forecast origins.

Table 2 separates source variables from the final model representation. The sequence models receive 14 × 8 inputs; BPNN and XGBoost use the flattened 112-element window, and SARIMAX uses load history plus the seven exogenous dimensions. Pressure and humidity are not part of this final schema. Sections 2.3.1 and 3.2.2 and the revised workflow/architecture figures describe these actual inputs. The S1 Code documentation is synchronized with the fixed-feature implementation.

### Changes in the manuscript

Table 2; Sections 2.2.2, 2.2.3, 2.3.1, and 3.2.2; Figs 3 and 4; deletion of former MIC Fig 10; renumbered Figs 10-12; S1 Code documentation.

### Revised text

Candidate predictors comprised historical daily load, meteorological variables, and calendar indicators, with their engineering relevance considered during model development.

The forecasting pipeline used a fixed eight-dimensional feature vector at each daily time step. It comprised the wavelet-denoised station load, temperature PC1, sine and cosine encodings of day of week and month, and weekend and holiday indicators. With a 14-day lookback, CNN-BiLSTM-Attention, LSTM, GRU, and the sequence-based ablation models received a 14 × 8 multivariate sequence. BPNN and XGBoost used the flattened 112-element window. SARIMAX used normalized load history and the seven transformed exogenous dimensions obtained by excluding contemporaneous load. The feature schema remained fixed across stations and forecast origins. MIC-style dependence scores served as an exploratory development-stage diagnostic and did not determine feature columns at individual forecast origins.

Temperature compression reduced the three correlated measurements to a single principal component in the final input, alongside the denoised load and calendar features.

## Comment 7

### Comment

In terms of the topic of forecast-to-dispatch, I wouldl ike to recommend a relevant paper, Feature-driven Economic Improvement for Network-constrained Unit Commitment: A Closed-loop Predict-and-optimize Framework, which may help authors enhance your literature section.

### Response

We added Chen et al. as reference [21] after evaluating its relevance to the forecasting-to-operation interface. Its closed-loop framework trains cost-oriented renewable-generation predictions using the objective and constraints of network-constrained unit commitment. The added Introduction paragraph distinguishes that closed-loop approach from the sequential forecast-guided inventory preparation and linepack-deviation objective used in this study.

The citation metadata were verified against the authors' institutional publication record: Chen X, Yang Y, Liu Y, Wu L; IEEE Transactions on Power Systems; 2022;37(4):3104-3118; doi:10.1109/TPWRS.2021.3128485.

### Changes in the manuscript

Introduction, paragraph following the literature on forecasting and dispatch; reference [21].

### Revised text

Closed-loop predict-and-optimize research links forecasting to the downstream decision objective. Chen et al. trained a cost-oriented renewable-generation predictor using the objective and constraints of network-constrained unit commitment [21]. Their work distinguishes predictive accuracy from operating cost. The present gas-network workflow uses sequential forecasting and inventory preparation with a linepack-deviation objective.

[21] Chen X, Yang Y, Liu Y, Wu L. Feature-driven economic improvement for network-constrained unit commitment: A closed-loop predict-and-optimize framework. IEEE Transactions on Power Systems. 2022;37(4):3104-3118. doi:10.1109/TPWRS.2021.3128485.
