# Supplementary material for: A forecast-to-dispatch workflow for day-ahead operation of a regional natural gas transmission network based on station-level load prediction and linepack target setting
Source: PLoS One. 2026 Oct 5;21(10):e0349981. doi: 10.1371/journal.pone.0349981 (PMC13637901; doi:10.1371/journal.pone.0349981)
Supplement: Peer review history

# Peer review: Decision Letter 0

**DOI:** 10.1371/journal.pone.0349981.r001

**Authors:** Zhengmao Li

1 Jul 2026

Dear Dr. Tian,

Thank you for submitting your manuscript to PLOS ONE. After careful consideration, we feel that it has merit but does not fully meet PLOS ONE’s publication criteria as it currently stands. Therefore, we invite you to submit a revised version of the manuscript that addresses the points raised during the review process.

==============================

**ACADEMIC EDITOR: Please revise**

==============================

Please submit your revised manuscript by Aug 30 2026 11:59PM. If you will need more time than this to complete your revisions, please reply to this message or contact the journal office at plosone@plos.org. When you're ready to submit your revision, log on to https://www.editorialmanager.com/pone/ and select the 'Submissions Needing Revision' folder to locate your manuscript file.

- A letter that responds to each point raised by the academic editor and reviewer(s). You should upload this letter as a separate file labeled 'Response to Reviewers'.
- A marked-up copy of your manuscript that highlights changes made to the original version. You should upload this as a separate file labeled 'Revised Manuscript with Track Changes'.
- An unmarked version of your revised paper without tracked changes. You should upload this as a separate file labeled 'Manuscript'.

If you would like to make changes to your financial disclosure, please include your updated statement in your cover letter. Guidelines for resubmitting your figure files are available below the reviewer comments at the end of this letter.

If applicable, we recommend that you deposit your laboratory protocols in protocols.io to enhance the reproducibility of your results. Protocols.io assigns your protocol its own identifier (DOI) so that it can be cited independently in the future. For instructions see: https://journals.plos.org/plosone/s/submission-guidelines#loc-laboratory-protocols. Additionally, PLOS ONE offers an option for publishing peer-reviewed Lab Protocol articles, which describe protocols hosted on protocols.io. Read more information on sharing protocols at https://plos.org/protocols?utm_medium=editorial-email&utm_source=authorletters&utm_campaign=protocols.

As the corresponding author, your ORCID iD is verified in the submission system and will appear in the published article. PLOS supports the use of ORCID, and we encourage all coauthors to register for an ORCID iD and use it as well. Please encourage your coauthors to verify their ORCID iD within the submission system before final acceptance, as unverified ORCID iDs will not appear in the published article. *Only* the individual author can complete the verification step; PLOS staff *cannot* verify ORCID iDs on behalf of authors.

We look forward to receiving your revised manuscript.

Kind regards,

Zhengmao Li

Academic Editor

PLOS One

Journal Requirements:

When submitting your revision, we need you to address these additional requirements.

1. Please ensure that your manuscript meets PLOS ONE's style requirements, including those for file naming. The PLOS ONE style templates can be found at

https://journals.plos.org/plosone/s/file?id=wjVg/PLOSOne_formatting_sample_main_body.pdfand https://journals.plos.org/plosone/s/file?id=ba62/PLOSOne_formatting_sample_title_authors_affiliations.pdf

2. PLOS requires an ORCID iD for the corresponding author in Editorial Manager on papers submitted after December 6th, 2016. Please ensure that you have an ORCID iD and that it is validated in Editorial Manager. To do this, go to ‘Update my Information’ (in the upper left-hand corner of the main menu), and click on the Fetch/Validate link next to the ORCID field. This will take you to the ORCID site and allow you to create a new iD or authenticate a pre-existing iD in Editorial Manager.

3. We note you use an AI tool to assist with your submission. Please update your AI use statement to include the following information in line with out policy (https://journals.plos.org/plosone/s/ethical-publishing-practice#loc-artificial-intelligence-tools-and-technologies). Please include:

1) The name(s) of any tools used

2) A description of how the authors used the tool(s)

3) How you evaluated the validity of the tool’s outputs

4) A clear statement of which aspects of the study, article contents, data, or supporting files were affected/generated by AI tool usage.

This should be either in your Methods or in the Acknowledgement section.

4. Please note that PLOS One has specific guidelines on code sharing for submissions in which author-generated code underpins the findings in the manuscript. In these cases, we expect all author-generated code to be made available without restrictions upon publication of the work. Please review our guidelines at https://journals.plos.org/plosone/s/materials-and-software-sharing#loc-sharing-code and ensure that your code is shared in a way that follows best practice and facilitates reproducibility and reuse.

5. In the online submission form, you indicated that the raw operational telemetry data used in this study were obtained from the Chongqing regional natural gas transmission network and contain commercially sensitive station-level production and pipeline-operation information. Therefore, the raw data cannot be made publicly available without authorization from the data owner. Station identifiers were anonymized in the manuscript. Aggregated and anonymized data sufficient to reproduce the main forecasting and linepack-analysis results, together with the core modeling scripts, are available through a controlled-access procedure subject to approval by the data owner. Requests for access may be directed to the corresponding author, Yuanyuan Tian, at tianyuanyuancnpc@163.com

All PLOS journals now require all data underlying the findings described in their manuscript to be freely available to other researchers, either 1. In a public repository, 2. Within the manuscript itself, or 3. Uploaded as supplementary information.

This policy applies to all data except where public deposition would breach compliance with the protocol approved by your research ethics board. If your data cannot be made publicly available for ethical or legal reasons (e.g., public availability would compromise patient privacy), please explain your reasons on resubmission and your exemption request will be escalated for approval.

6. Please amend your list of authors on the manuscript to ensure that each author is linked to an affiliation. Authors’ affiliations should reflect the institution where the work was done (if authors moved subsequently, you can also list the new affiliation stating “current affiliation:….” as necessary).

7. We notice that your supplementary [tables] are included in the manuscript file. Please remove them and upload them with the file type 'Supporting Information'. Please ensure that each Supporting Information file has a legend listed in the manuscript after the references list.

8. We notice that your supplementary figures are uploaded with the file type 'Figure'. Please amend the file type to 'Supporting Information'. Please ensure that each Supporting Information file has a legend listed in the manuscript after the references list.

9. If the reviewer comments include a recommendation to cite specific previously published works, please review and evaluate these publications to determine whether they are relevant and should be cited. There is no requirement to cite these works unless the editor has indicated otherwise.

[Note: HTML markup is below. Please do not edit.]

Reviewers' comments:

Reviewer's Responses to Questions

**Comments to the Author**

1. Is the manuscript technically sound, and do the data support the conclusions?

Reviewer #1: Yes

Reviewer #2: Partly

**********

2. Has the statistical analysis been performed appropriately and rigorously?

Reviewer #1: Yes

Reviewer #2: N/A

**********

3. Have the authors made all data underlying the findings in their manuscript fully available?

The PLOS Data policy

Reviewer #1: Yes

Reviewer #2: No

**********

4. Is the manuscript presented in an intelligible fashion and written in standard English?

Reviewer #1: Yes

Reviewer #2: Yes

**********

Reviewer #1: 1. The main contribution of this paper lies in “engineering integration” rather than “algorithmic innovation.” CNN-BiLSTM-Attention, intraday coefficient reconstruction, and PSO optimization are all established technologies; the core value lies in integrating these three into a practical day-ahead scheduling workflow.

2. The intraday allocation coefficients are based on historical average patterns and may fail during holidays, sudden changes in industrial load, or accident scenarios. Although this limitation is mentioned in the paper, no quantitative assessment is provided (e.g., whether reconstruction errors increase significantly during holidays). It is recommended to supplement the analysis with an examination of reconstruction errors on abnormal days or to discuss how to dynamically update the allocation coefficients.

3. The literature review lacks comprehensiveness in its introduction of relevant methodologies. It is recommended to cite the following works: Multimodal Heterogeneous Signal Fusion Detection (https://doi.org/10.1016/j.ymssp.2026.113923) and Zero-Sample Detection (https://doi.org/10.1080/10589759. 2025.2534429; https://doi.org/10.1016/j.eswa.2025.128592), lightweight explainable detection (10.1088/1361-6501/acd5ef; https://doi.org/10.1007/s11071-024-10157-1; https://ieeexplore.ieee.org/abstract/document/10473026), physics-based prior neural network design (https://doi.org/10.1016/j.ress.2023.109793), etc.

4. The objective function minimizes only the mean absolute deviation between the actual pipeline storage and the target, without incorporating economic or safety factors such as compressor energy consumption, gas procurement costs, or penalties for pressure fluctuations. This may lead to oscillations near the boundary of the target pipeline. It is recommended to discuss the practical feasibility of the solutions obtained without economic objectives, or to indicate future directions for integrating economic scheduling.

5. Although walk-forward validation was employed, were feature selection (MIC), PCA dimensionality reduction, and normalization parameters strictly fitted using only the training set in each iteration?

6. Ablation experiments showed that the contributions of different components vary across sites (Attention-only performs better at stable sites, while CNN+Attention performs better at volatile sites), but this conclusion was not used to guide actual deployment—should different models be selected for different sites?

7. Including site G, which has a 56% data missing rate, in the evaluation and reporting a 91% MAPE—while valuable as a “borderline case”—may mislead readers into believing the model performs poorly.

8. Reporting MAPE, MedianAPE, MAE, and RMSE simultaneously is problematic, as MAPE is prone to being inflated by outliers during low-load periods, whereas MedianAPE is more suitable for asymmetric distributions.

9. There are multiple instances of text repetition (e.g., the title of Figure 9 is repeated nine times in a row), and the MIC matrix in Figure 10 is not clearly interpreted in the main text (it merely states “MIC values are 0.91…” without labeling the variable names).

10. The introduction cites a large number of references on natural gas load forecasting and pipeline network scheduling, but the results and discussion section lacks quantitative comparisons with similar methods (e.g., direct use of LSTM with intraday rolling scheduling, or pipeline inventory optimization based on model predictive control).

Reviewer #2: The manuscript presents a forecast-to-dispatch workflow for day-ahead operation of a regional natural gas transmission network. It integrates station-level daily gas load forecasting, daily-to-hourly demand profile reconstruction, and linepack-oriented target setting for representative pipeline segments. The topic is relevant to practical gas-network dispatch, and the attempt to connect load forecasting results with dispatch-relevant linepack and pressure targets is of engineering interest. Overall, the manuscript has a clear application background and potentially useful practical value. However, several technical aspects still require further clarification, validation, and strengthening before the conclusions can be considered fully convincing.

1.The boundary of the proposed forecast-to-dispatch workflow should be clarified more explicitly.

The manuscript emphasizes that the proposed method is not a fully integrated end-to-end control optimization framework, but rather a sequential workflow consisting of forecasting, hourly demand reconstruction, and linepack target setting. The authors should more clearly distinguish forecast outputs, reconstructed operational inputs, decision variables, and dispatch-support outputs. This clarification is necessary to avoid the impression that the forecasting model and the dispatch optimization module are only loosely connected.

2.More quantitative details should be provided for missing-data handling and anomaly treatment.

The manuscript reports that Station G has a missing-data rate of 56.18%, but the missing-data rates and anomaly proportions of the other stations are not sufficiently documented. The authors should provide a station-wise summary of missing values, outliers, and imputation results. The parameters of the KNN imputation method should also be reported. In addition, the manuscript should explain whether Station G affects the overall performance statistics and why it is retained rather than excluded from the main evaluation.

3.The leakage-control procedure should be described in a more reproducible way.

Although the manuscript states that walk-forward validation with leakage control was adopted, the exact implementation remains insufficiently detailed. The authors should provide a step-by-step workflow, table, or pseudo-code showing how the training, validation, and test windows are constructed. It should also be clearly stated at which stage KNN imputation, normalization, MIC-based feature screening, PCA, and wavelet denoising are fitted and applied. This is important because any preprocessing step using future information may lead to optimistic forecasting results.

4.The suitability of the deep-learning architecture under a limited sample size should be further justified.

Each station appears to contain only about 540 daily observations, whereas the proposed CNN-BiLSTM-Attention model is relatively complex. The authors should provide the number of trainable parameters, training and validation loss curves, early-stopping behavior, or additional evidence showing that overfitting is well controlled. This is particularly important for relatively regular stations, where simpler models may already perform competitively.

5.The comparison with baseline models should be strengthened.

The manuscript compares the proposed model with SARIMAX, XGBoost, BPNN, LSTM, and GRU models. However, the hyperparameter selection procedure for these baselines should be described more rigorously. The authors should clarify the search space, tuning criterion, and whether all models use the same validation protocol. Statistical significance tests, bootstrap confidence intervals, or error-distribution plots over the hold-out period would make the comparison more convincing than reporting only average MAPE, MAE, and RMSE values.

6.The daily-to-hourly demand reconstruction method requires broader validation.

The use of historical intraday allocation coefficients is a key interface between daily forecasting and linepack analysis. At present, the validation is mainly reported for three pipeline segments and 18 validation days. The authors should test the reconstruction rule across different seasons, weekdays, holidays, and abnormal load conditions. In addition, the operational impact of a 1–1.5 h peak-timing error should be analyzed, especially its effect on target linepack and average pressure.

7.The physical constraints in the linepack optimization model should be described more completely.

The current optimization model focuses on target linepack and average pressure, but the treatment of hydraulic feasibility remains relatively simplified. The authors should clarify whether the model is steady-state, quasi-steady-state, or transient. Important elements such as gas-flow equations, pressure-drop relationships, inlet supply constraints, valve operations, flow-direction changes, and nodal boundary conditions should be described in greater detail. Without these details, the dispatch feasibility of the optimized targets is difficult to fully assess.

8.The PSO configuration appears too limited and needs further justification.

The PSO algorithm uses only 20 particles and 10 iterations, which may be insufficient for reliable optimization, even if the decision space is relatively small. The authors should provide convergence curves, repeated-run statistics, and sensitivity tests for swarm size and iteration number. A comparison with grid search or another deterministic search method would also help verify that the reported target linepack values are not artifacts of a particular random optimization run.

9.Uncertainty propagation from forecasting errors to dispatch targets should be added.

The manuscript currently reports point forecasts, point-based hourly reconstruction results, and point estimates of linepack and pressure targets. In practical dispatch, however, operators are more concerned with risk under forecast uncertainty. The authors should conduct scenario or sensitivity analysis, for example by perturbing daily demand forecasts by ±5% and ±10%, or shifting the peak hour by 1–2 h. The analysis should examine whether the optimized linepack and pressure targets remain within safe operating zones under these forecast-error scenarios.

**********

what does this mean?). If published, this will include your full peer review and any attached files.

If you choose “no”, your identity will remain anonymous but your review may still be made public.

**Do you want your identity to be public for this peer review?** For information about this choice, including consent withdrawal, please see our Privacy Policy

Reviewer #1: No

Reviewer #2: No

**********

[NOTE: If reviewer comments were submitted as an attachment file, they will be attached to this email and accessible via the submission site. Please log into your account, locate the manuscript record, and check for the action link "View Attachments". If this link does not appear, there are no attachment files.]

To ensure your figures meet our technical requirements, please review our figure guidelines: https://journals.plos.org/plosone/s/figures

You may also use PLOS’s free figure tool, NAAS, to help you prepare publication quality figures: https://journals.plos.org/plosone/s/figures#loc-tools-for-figure-preparation.

NAAS will assess whether your figures meet our technical requirements by comparing each figure against our figure specifications.

# Author comment: Author response to Decision Letter 1

**DOI:** 10.1371/journal.pone.0349981.r002

22 Aug 2026

Response to the Academic Editor and Reviewers

PONE-D-26-22124

A forecast-to-dispatch workflow for day-ahead operation of a regional natural gas transmission network based on station-level load prediction and linepack target setting

Overview of the revision. The manuscript now presents the contribution as an engineering integration; defines the forecast-to-dispatch boundary; documents a leakage-controlled 30-origin evaluation; separates routine stations from data-quality boundary cases; strengthens baseline and PSO checks; narrows the linepack formulation to its reduced-order quasi-steady scope; and supplies separately uploaded tables, figures, code, and anonymized data. Unsupported seasonal and transient uncertainty values were removed rather than inferred.

Journal requirements

Journal Requirement 1: PLOS ONE style and file naming

Comment: Please ensure that your manuscript meets PLOS ONE's style requirements, including those for file naming.

Response and revision made: The manuscript has been reformatted as a single-column, double-spaced Word file with continuous line numbering and page numbers. The title is in sentence case, a short title has been added, the Methods heading has been standardized, the reference list has been converted to Vancouver style, and Supporting Information legends appear after the references. The submission package uses explicit PLOS upload labels and file names.

Journal Requirement 2: ORCID

Comment: PLOS requires an ORCID iD for the corresponding author in Editorial Manager on papers submitted after December 6th, 2016. Please ensure that you have an ORCID iD and that it is validated in Editorial Manager.

Response and revision made: The corresponding author will verify the ORCID iD through the Fetch/Validate link in Editorial Manager before completing resubmission. Coauthors have also been asked to verify their own ORCID records where available. This account-level action cannot be completed by a coauthor or by the manuscript-preparation software and is listed in the submission checklist.

Journal Requirement 3: AI use statement

Comment: We note you use an AI tool to assist with your submission. Please update your AI use statement to include the name(s) of any tools used, a description of how the authors used the tool(s), how you evaluated the validity of the tool's outputs, and a clear statement of which aspects of the study, article contents, data, or supporting files were affected or generated by AI tool usage.

Response and revision made: The Acknowledgments now name ChatGPT and Codex (OpenAI), describe their use for English editing and for organizing, reviewing, and documenting author-supplied analysis code, explain that the authors checked all outputs against project records and engineering context, and state that the tools did not create source observations or make scientific decisions. The authors accept responsibility for the manuscript, calculations, code, tables, figures, and supporting files.

Journal Requirement 4: Code sharing

Comment: Please note that PLOS ONE has specific guidelines on code sharing for submissions in which author-generated code underpins the findings in the manuscript. In these cases, we expect all author-generated code to be made available without restrictions upon publication of the work. Please review our guidelines and ensure that your code is shared in a way that follows best practice and facilitates reproducibility and reuse.

Response and revision made: S1 Code contains the author-generated scripts used for the released forecasting, preprocessing, ablation, bootstrap, model-parameter, PSO robustness, and plotting workflows. It includes fixed seeds, configuration files, pinned requirements, recorded outputs, smoke tests, and a README. Using the anonymized supporting inputs, the package reproduces the computational workflow and the main analysis procedures. It does not claim to reconstruct restricted absolute telemetry or a transient hydraulic simulator.

Journal Requirement 5: Data availability

Comment: All PLOS journals require all data underlying the findings described in the manuscript to be freely available to other researchers, either in a public repository, within the manuscript itself, or as Supporting Information. If the data cannot be made publicly available for ethical or legal reasons, please explain the reasons on resubmission so that an exemption request can be escalated for approval.

Response and revision made: Raw station telemetry, exact asset identifiers, detailed pressure and flow trajectories, pipeline configuration, and site-specific operating limits are owned by the data provider and are subject to commercial-confidentiality and pipeline-security restrictions. We request approval of this data-policy exemption. S1 Data releases the maximum non-sensitive material available to the authors: anonymized indexed daily station data, 30-day predictions and metrics, ablation and bootstrap outputs, PSO summaries, archived aggregate reconstruction and linepack-target results, a data dictionary, and a restriction statement. The revised Data Availability statement distinguishes recalculable public outputs from restricted archived aggregates.

Journal Requirement 6: Author affiliations

Comment: Please amend your list of authors on the manuscript to ensure that each author is linked to an affiliation. Authors' affiliations should reflect the institution where the work was done.

Response and revision made: The title page now links every author to a numbered affiliation: Ying Liu and Yuanyuan Tian to affiliation 1, Yong Xiao to affiliation 2, Zhiping Tang to affiliation 3, Simin Zhong to affiliation 4, and Chenguang Yong to affiliation 5. The corresponding author is identified separately with an e-mail address.

Journal Requirement 7: Supporting tables

Comment: We notice that your supplementary tables are included in the manuscript file. Please remove them and upload them with the file type 'Supporting Information'. Please ensure that each Supporting Information file has a legend listed in the manuscript after the references list.

Response and revision made: The supplementary tables have been removed from the main manuscript and prepared as five separate Supporting Information files (S1-S5 Tables). Their legends are listed after the references. S1-S4 Tables report station-level metrics, and S5 Table reports model-wise routine-station summaries and bootstrap intervals.

Journal Requirement 8: Supporting figures

Comment: We notice that your supplementary figures are uploaded with the file type 'Figure'. Please amend the file type to 'Supporting Information'. Please ensure that each Supporting Information file has a legend listed in the manuscript after the references list.

Response and revision made: S1-S4 Figs are supplied as separate Supporting Information files rather than main-figure files. Their legends are listed after the references, and the upload manifest identifies the required Supporting Information file type.

Journal Requirement 9: Recommended references

Comment: If the reviewer comments include a recommendation to cite specific previously published works, please review and evaluate these publications to determine whether they are relevant and should be cited. There is no requirement to cite these works unless the editor has indicated otherwise.

Response and revision made: We evaluated the recommended publications individually. A concise Introduction paragraph now cites the seven studies that are methodologically relevant to heterogeneous signal fusion, limited abnormal samples, lightweight interpretability, and prior-informed network design [21-27]. The revised text treats these studies as cross-domain methodological context rather than as direct natural-gas forecasting benchmarks.

Reviewer #1

Reviewer #1 Comment 1: Nature of the contribution

Comment: The main contribution of this paper lies in 'engineering integration' rather than 'algorithmic innovation.' CNN-BiLSTM-Attention, intraday coefficient reconstruction, and PSO optimization are all established technologies; the core value lies in integrating these three into a practical day-ahead scheduling workflow.

Response and revision made: We agree. The Abstract, Introduction, workflow description, Discussion, and Conclusions now identify the contribution as an engineering integration and evaluation of established components. Claims of algorithmic novelty have been removed. Fig 1 and Section 2.1 present the operational interface from daily station forecasts to hourly withdrawals and reduced-order segment targets.

Reviewer #1 Comment 2: Intraday allocation coefficients

Comment: The intraday allocation coefficients are based on historical average patterns and may fail during holidays, sudden changes in industrial load, or accident scenarios. Although this limitation is mentioned in the paper, no quantitative assessment is provided, such as whether reconstruction errors increase significantly during holidays. It is recommended to supplement the analysis with an examination of reconstruction errors on abnormal days or to discuss how to dynamically update the allocation coefficients.

Response and revision made: The manuscript now states that the coefficients were estimated from preceding training data only and should be updated on a rolling basis when recent complete hourly observations become available. It also specifies operator review for holidays, abrupt industrial-load changes, and emergency operation. We retained the traceable archived 18-day segment validation and did not report the earlier placeholder abnormal-day table because the archive does not preserve date-resolved abnormal-day reconstruction records. This limitation and the need for broader seasonal and abnormal-day validation are stated explicitly.

Reviewer #1 Comment 3: Literature review

Comment: The literature review lacks comprehensiveness in its introduction of relevant methodologies. It is recommended to cite the following works: Multimodal Heterogeneous Signal Fusion Detection (https://doi.org/10.1016/j.ymssp.2026.113923) and Zero-Sample Detection (https://doi.org/10.1080/10589759.2025.2534429; https://doi.org/10.1016/j.eswa.2025.128592), lightweight explainable detection (10.1088/1361-6501/acd5ef; https://doi.org/10.1007/s11071-024-10157-1; https://ieeexplore.ieee.org/abstract/document/10473026), physics-based prior neural network design (https://doi.org/10.1016/j.ress.2023.109793), etc.

Response and revision made: The Introduction has been expanded with a concise cross-domain paragraph covering heterogeneous signal fusion, limited or zero abnormal samples, lightweight interpretable networks, and prior-informed prediction [21-27]. The discussion connects these directions to heterogeneous station telemetry, data-quality boundaries, and interpretable engineering deployment without treating fault-diagnosis studies as direct gas-load benchmarks.

Reviewer #1 Comment 4: Objective function

Comment: The objective function minimizes only the mean absolute deviation between the actual pipeline storage and the target, without incorporating economic or safety factors such as compressor energy consumption, gas procurement costs, or penalties for pressure fluctuations. This may lead to oscillations near the boundary of the target pipeline. It is recommended to discuss the practical feasibility of the solutions obtained without economic objectives, or to indicate future directions for integrating economic scheduling.

Response and revision made: Sections 2.5 and 3.6 now define the calculation as reduced-order linepack target setting, not full economic dispatch. Pressure and linepack envelopes are enforced as feasibility conditions, and the resulting targets require operator review. The objective does not include compressor energy, procurement cost, or pressure-fluctuation penalties; this limitation is stated in the Results and Limitations, where integration with economic scheduling or MPC is identified as future work.

Reviewer #1 Comment 5: Leakage control

Comment: Although walk-forward validation was employed, were feature selection (MIC), PCA dimensionality reduction, and normalization parameters strictly fitted using only the training set in each iteration?

Response and revision made: Yes. Sections 2.2.3 and 2.6.1 and Fig 3 now state that imputation, normalization, nonlinear relevance screening, wavelet parameters, PCA loadings, and model parameters are estimated from the available historical model-fitting data at each origin. The fitted transformations are then applied to the chronological validation block and next-day input. The held-out next-day target is used only for scoring. For SARIMAX, known next-day calendar indicators and a one-day persistence forecast of temperature are used; observed test-day weather is not used. S1 Code records these boundaries.

Reviewer #1 Comment 6: Site-specific model deployment

Comment: Ablation experiments showed that the contributions of different components vary across sites (Attention-only performs better at stable sites, while CNN+Attention performs better at volatile sites), but this conclusion was not used to guide actual deployment—should different models be selected for different sites?

Response and revision made: We agree. The ablation interpretation now states that component utility is station dependent. The recalculated baseline comparison likewise shows that the best model differs by station. The deployment recommendation has therefore been revised from a single universal architecture to validation-based station-specific selection, with simpler models retained at regular stations when they perform competitively.

Reviewer #1 Comment 7: Station G

Comment: Including site G, which has a 56% data missing rate, in the evaluation and reporting a 91% MAPE—while valuable as a 'borderline case'—may mislead readers into believing the model performs poorly.

Response and revision made: Station G is now designated a pre-specified data-quality boundary case and is excluded from routine-performance averages, bootstrap inference, and ordinary-station conclusions. Its results are reported separately to show the limit imposed by severe telemetry incompleteness. Station K is excluded from the common 30-day evaluation because its usable history is insufficient. The Abstract, Tables 1 and 8-9, Results, and Conclusions use this separation consistently.

Reviewer #1 Comment 8: Error metrics

Comment: Reporting MAPE, MedianAPE, MAE, and RMSE simultaneously is problematic, as MAPE is prone to being inflated by outliers during low-load periods, whereas MedianAPE is more suitable for asymmetric distributions.

Response and revision made: The revised manuscript assigns distinct roles to the four metrics. MAPE remains the primary relative metric for comparability, MedianAPE is reported as the robust measure of typical percentage error, and MAE and RMSE retain physical-scale information and sensitivity to large errors. Station G is interpreted using both MAPE and MedianAPE and is not included in routine averages. The text no longer treats the metrics as interchangeable.

Reviewer #1 Comment 9: Repetition and MIC interpretation

Comment: There are multiple instances of text repetition (e.g., the title of Figure 9 is repeated nine times in a row), and the MIC matrix in Figure 10 is not clearly interpreted in the main text (it merely states 'MIC values are 0.91...' without labeling the variable names).

Response and revision made: The repeated Figure 9 caption text has been removed. The Fig 10 discussion now identifies the variables explicitly: daily load, average, minimum, and maximum temperature, air pressure, humidity, and calendar variables. It reports the strongest load associations and the redundancy among temperature variables, then links these findings to the relevance-screening and PCA steps.

Reviewer #1 Comment 10: Quantitative comparison

Comment: The introduction cites a large number of references on natural gas load forecasting and pipeline network scheduling, but the results and discussion section lacks quantitative comparisons with similar methods (e.g., direct use of LSTM with intraday rolling scheduling, or pipeline inventory optimization based on model predictive control).

Response and revision made: The forecasting comparison has been recalculated under one common 30-origin walk-forward protocol for SARIMAX, XGBoost, BPNN, LSTM, GRU, and CNN-BiLSTM-Attention. Complete station metrics are provided in S1-S4 Tables and the routine-station summary and paired bootstrap intervals in S5 Table. MPC was not used as a numerical benchmark because the archived target-setting formulation does not contain the closed-loop states and control actions required for a matched comparison. The manuscript now states this scope difference and avoids claiming equivalence to MPC dispatch.

Reviewer #2

Reviewer #2 Comment 1: Workflow boundary

Comment: The boundary of the proposed forecast-to-dispatch workflow should be clarified more explicitly. The manuscript emphasizes that the proposed method is not a fully integrated end-to-end control optimization framework, but rather a sequential workflow consisting of forecasting, hourly demand reconstruction, and linepack target setting. The authors should more clearly distinguish forecast outputs, reconstructed operational inputs, decision variables, and dispatch-support outputs. This clarification is necessary to avoid the impression that the forecasting model and the dispatch optimization module are only loosely connected.

Response and revision made: Fig 1 and Section 2.1 now label the daily station forecast as the forecast output, the reconstructed hourly nodal withdrawal profile as the operational input to target setting, the segment target linepack as the decision variable, and target linepack, average pressure, operating zone, and packing or drafting direction as dispatch-support outputs. The text states that the modules are sequentially coupled through these explicit quantities and that the workflow is not an end-to-end closed-loop controller.

Reviewer #2 Comment 2: Missing data and anomalies

Comment: More quantitative details should be provided for missing-data handling and anomaly treatment. The manuscript reports that Station G has a missing-data rate of 56.18%, but the missing-data rates and anomaly proportions of the other stations are not sufficiently documented. The authors should provide a station-wise summary of missing values, outliers, and imputation results. The parameters of the KNN imputation method should also be reported. In addition, the manuscript should explain whether Station G affects the overall performance statistics and why it is retained rather than excluded from the main evaluation.

Response and revision made: Table 1 reports the archived source-point missing rate and complete-day missing proportion for all 11 stations and now adds a reproducible aggregate-daily audit of outlier screening and imputation. Under the Q1 - 3IQR and Q3 + 3IQR rule, the released daily series contained one flagged record at Station D (0.19%) and none at the other stations; distance-weighted KNN with three neighbors filled all flagged entries, leaving zero residual missing values among archived daily rows. The table note distinguishes these aggregate-daily counters from the source-point telemetry summaries and from the split-specific training-only preprocessing used in evaluation. Station G remains a separately reported data-quality boundary case and is excluded from routine-performance inference; Station K is excluded from the common 30-day evaluation because only 62 daily observations were available.

Reviewer #2 Comment 3: Leakage-control procedure

Comment: The leakage-control procedure should be described in a more reproducible way. Although the manuscript states that walk-forward validation with leakage control was adopted, the exact implementation remains insufficiently detailed. The authors should provide a step-by-step workflow, table, or pseudo-code showing how the training, validation, and test windows are constructed. It should also be clearly stated at which stage KNN imputation, normalization, MIC-based feature screening, PCA, and wavelet denoising are fitted and applied. This is important because any preprocessing step using future information may lead to optimistic forecasting results.

Response and revision made: Sections 2.2.3 and 2.6.1 and Fig 3 specify a 14-day lookback, a fixed final 30-observation hold-out period, and one-day expanding-origin evaluation. At every origin, the most recent 10% of available historical observations, with a minimum of 14, forms the chronological validation block. Outlier thresholds, KNN imputation, normalization, nonlinear relevance screening, wavelet parameters, PCA, and model fitting use no next-day target information. The fitted transformations are applied to validation and next-day inputs. SARIMAX uses known calendar indicators and a one-day persistence temperature forecast, not observed test-day weather. S1 Code records the preprocessing boundary at every origin.

Reviewer #2 Comment 4: Limited sample size and overfitting

Comment: The suitability of the deep-learning architecture under a limited sample size should be further justified. Each station appears to contain only about 540 daily observations, whereas the proposed CNN-BiLSTM-Attention model is relatively complex. The authors should provide the number of trainable parameters, training and validation loss curves, early-stopping behavior, or additional evidence showing that overfitting is well controlled. This is particularly important for relatively regular stations, where simpler models may already perform competitively.

Response and revision made: The Methods now report 116,641 trainable parameters for the selected configuration. Dropout, chronological validation, and early stopping with a patience of 10 epochs were used. S3 Fig presents representative training and validation MSE histories for Stations A, F, and G. The revised Results also report that simpler baselines frequently match or outperform the hybrid model, and deployment guidance now favors station-specific validation rather than universal use of the deepest architecture.

Reviewer #2 Comment 5: Baseline comparison

Comment: The comparison with baseline models should be strengthened. The manuscript compares the proposed model with SARIMAX, XGBoost, BPNN, LSTM, and GRU models. However, the hyperparameter selection procedure for these baselines should be described more rigorously. The authors should clarify the search space, tuning criterion, and whether all models use the same validation protocol. Statistical significance tests, bootstrap confidence intervals, or error-distribution plots over the hold-out period would make the comparison more convincing than reporting only average MAPE, MAE, and RMSE values.

Response and revision made: Table 7 reports the fixed configurations used for all six model families and the chronological validation role. Candidate configurations were evaluated only within the historical development data and were frozen before the common 30-day hold-out evaluation; the public package does not claim an exhaustive search that was not preserved in the archive. All models use identical forecast origins and preprocessing boundaries. SARIMAX uses a one-day persistence forecast for next-day temperature and known calendar indicators, so observed test-day weather does not enter the comparison. S5 Table provides paired station-level bootstrap intervals across the nine routine stations, while S1-S4 Tables give the complete station-level error metrics. After this correction, SARIMAX achieved a mean station-level MAPE of 8.61% across the nine routine stations.

Reviewer #2 Comment 6: Daily-to-hourly reconstruction

Comment: The daily-to-hourly demand reconstruction method requires broader validation. The use of historical intraday allocation coefficients is a key interface between daily forecasting and linepack analysis. At present, the validation is mainly reported for three pipeline segments and 18 validation days. The authors should test the reconstruction rule across different seasons, weekdays, holidays, and abnormal load conditions. In addition, the operational impact of a 1-1.5 h peak-timing error should be analyzed, especially its effect on target linepack and average pressure.

Response and revision made: We retained the traceable archived 18-day validation for LFFX, NGXDD, and DLFX, including hourly MAPE, cumulative daily error, peak-timing error, and peak-load error. The manuscript does not report the earlier placeholder seasonal analysis because date-resolved reconstruction outputs were unavailable. It now states that broader seasonal and abnormal-day validation remains necessary. A quantitative transient pressure effect was also not reported because the archive lacks synchronized hourly boundary data and a calibrated transient model; the corresponding limitation replaces unsupported sensitivity claims.

Reviewer #2 Comment 7: Physical constraints

Comment: The physical constraints in the linepack optimization model should be described more completely. The current optimization model focuses on target linepack and average pressure, but the treatment of hydraulic feasibility remains relatively simplified. The authors should clarify whether the model is steady-state, quasi-steady-state, or transient. Important elements such as gas-flow equations, pressure-drop relationships, inlet supply constraints, valve operations, flow-direction changes, and nodal boundary conditions should be described in greater detail. Without these details, the dispatch feasibility of the optimized targets is difficult to fully assess.

Response and revision made: The formulation is now identified as reduced-order and quasi-steady, not transient. The Methods define the segment-average pressure and linepack relationships, prescribed inlet supply and forecast-derived withdrawals, pressure and linepack envelopes, fixed network configuration, and the representative-segment scope. The text also identifies excluded functions: transient nodal trajectories, valve switching, compressor dispatch, flow reversal, and time-varying nodal boundary optimization. Fig 13 and Table 6 are harmonized with the archived targets and search intervals, and field use is conditioned on operator review and, where available, transient-model verification.

Reviewer #2 Comment 8: PSO configuration

Comment: The PSO configuration appears too limited and needs further justification. The PSO algorithm uses only 20 particles and 10 iterations, which may be insufficient for reliable optimization, even if the decision space is relatively small. The authors should provide convergence curves, repeated-run statistics, and sensitivity tests for swarm size and iteration number. A comparison with grid search or another deterministic search method would also help verify that the reported target linepack values are not artifacts of a particular random optimization run.

Response and revision made: Each PSO budget was repeated 30 times with fixed documented seeds. Table 13 compares 20 particles x 10 iterations, 40 x 10, and 20 x 30 and reports target and objective stability against a deterministic grid over the same bounds. S4 Fig reports the mean best-so-far convergence trajectory and standard deviation for the reference setting. For LFFX, the archived reduced-order objective has an equivalent-minimum interval containing the reported 1.272 x 10^6 m3 target. Run-level outputs and the deterministic check are supplied in S1 Code and S1 Data.

Reviewer #2 Comment 9: Uncertainty propagation

Comment: Uncertainty propagation from forecasting errors to dispatch targets should be added. The manuscript currently reports point forecasts, point-based hourly reconstruction results, and point estimates of linepack and pressure targets. In practical dispatch, however, operators are more concerned with risk under forecast uncertainty. The authors should conduct scenario or sensitivity analysis, for example by perturbing daily demand forecasts by +/-5% and +/-10%, or shifting the peak hour by 1-2 h. The analysis should examine whether the optimized linepack and pressure targets remain within safe operating zones under these forecast-error scenarios.

Response and revision made: We agree that scenario-based uncertainty propagation is required before operational deployment. The available archive does not contain the synchronized date-resolved hydraulic boundaries and calibrated transient model needed to translate these perturbations into defensible pressure and flow trajectories. We therefore did not retain numerical placeholder scenarios. The contribution has been narrowed to point-based reduced-order day-ahead target setting, unsupported sensitivity values have been removed, and the Methods, Results, and Limitations identify forecast-scenario propagation with calibrated transient hydraulics as required future validation.

Attachment Submitted filename: 03_Response_to_Reviewers_PONE-D-26-22124.docx

# Peer review: Decision Letter 1

**DOI:** 10.1371/journal.pone.0349981.r003

**Authors:** Zhengmao Li

6 Sep 2026

Dear Dr. Tian,

Thank you for submitting your manuscript to PLOS One. After careful consideration, we feel that it has merit but does not fully meet PLOS One’s publication criteria as it currently stands. Therefore, we invite you to submit a revised version of the manuscript that addresses the points raised during the review process.

Please submit your revised manuscript by Oct 21 2026 11:59PM. If you will need more time than this to complete your revisions, please reply to this message or contact the journal office at plosone@plos.org. When you're ready to submit your revision, log on to https://www.editorialmanager.com/pone/ and select the 'Submissions Needing Revision' folder to locate your manuscript file.

- A letter that responds to each point raised by the academic editor and reviewer(s). You should upload this letter as a separate file labeled 'Response to Reviewers'.
- A marked-up copy of your manuscript that highlights changes made to the original version. You should upload this as a separate file labeled 'Revised Manuscript with Track Changes'.
- An unmarked version of your revised paper without tracked changes. You should upload this as a separate file labeled 'Manuscript'.

If you would like to make changes to your financial disclosure, please include your updated statement in your cover letter. Guidelines for resubmitting your figure files are available below the reviewer comments at the end of this letter.

If applicable, we recommend that you deposit your laboratory protocols in protocols.io to enhance the reproducibility of your results. Protocols.io assigns your protocol its own identifier (DOI) so that it can be cited independently in the future. For instructions see: https://journals.plos.org/plosone/s/submission-guidelines#loc-laboratory-protocols. Additionally, PLOS One offers an option for publishing peer-reviewed Lab Protocol articles, which describe protocols hosted on protocols.io. Read more information on sharing protocols at https://plos.org/protocols?utm_medium=editorial-email&utm_source=authorletters&utm_campaign=protocols.

As the corresponding author, your ORCID iD is verified in the submission system and will appear in the published article. PLOS supports the use of ORCID, and we encourage all coauthors to register for an ORCID iD and use it as well. Please encourage your coauthors to verify their ORCID iD within the submission system before final acceptance, as unverified ORCID iDs will not appear in the published article. *Only* the individual author can complete the verification step; PLOS staff *cannot* verify ORCID iDs on behalf of authors.

We look forward to receiving your revised manuscript.

Kind regards,

Zhengmao Li

Academic Editor

PLOS One

Journal Requirements:

If the reviewer comments include a recommendation to cite specific previously published works, please review and evaluate these publications to determine whether they are relevant and should be cited. There is no requirement to cite these works unless the editor has indicated otherwise.

Please review your reference list to ensure that it is complete and correct. If you have cited papers that have been retracted, please include the rationale for doing so in the manuscript text, or remove these references and replace them with relevant current references. Any changes to the reference list should be mentioned in the rebuttal letter that accompanies your revised manuscript. If you need to cite a retracted article, indicate the article’s retracted status in the References list and also include a citation and full reference for the retraction notice.

Additional Editor Comments:

Please revise

[Note: HTML markup is below. Please do not edit.]

Reviewers' comments:

Reviewer's Responses to Questions

**Comments to the Author**

Reviewer #3: All comments have been addressed

Reviewer #4: (No Response)

**********

2. Is the manuscript technically sound, and do the data support the conclusions?

Reviewer #3: Yes

Reviewer #4: Yes

**********

3. Has the statistical analysis been performed appropriately and rigorously?

Reviewer #3: Yes

Reviewer #4: Yes

**********

4. Have the authors made all data underlying the findings in their manuscript fully available?

The PLOS Data policy

Reviewer #3: Yes

Reviewer #4: Yes

**********

5. Is the manuscript presented in an intelligible fashion and written in standard English?

Reviewer #3: Yes

Reviewer #4: Yes

**********

Reviewer #3: The revised manuscript has been substantially improved in response to the previous comments, particularly in clarifying the engineering-integration contribution, strengthening the forecasting benchmarks, and narrowing the scope of the linepack model. However, several important issues remain regarding the end-to-end traceability of the forecast-to-dispatch workflow, the consistency of forecasting-model deployment, and the mathematical formulation of the reduced-order linepack target-setting model.

1.The forecasting evaluation uses a 30-day walk-forward test, whereas the temporal-disaggregation validation is based on a separate archived 18-day dataset. It remains unclear whether the reported linepack targets are generated from the same forecast origins evaluated in the forecasting section. At least one fully traceable example from station forecast → hourly reconstruction → segment demand → linepack target should be provided.

2.The manuscript should explain more explicitly how source-point missingness is propagated through hourly imputation, station aggregation, and daily-load construction, and report station-level completeness before and after imputation.

3.The authors should either implement station-specific model selection in the actual forecast-to-dispatch workflow or clearly justify why the hybrid model is retained for downstream target setting.

4.A more fundamental issue exists in Eq. (22). The equation is written as an instantaneous flow/mass-balance constraint but contains no intertemporal linepack accumulation term. Therefore, it is unclear how Eq. (22) can “generate the 24-h linepack trajectory” as stated in Section 2.5.3. A state-transition relation such as V(k+1) = V(k) + Δt × (inflow − outflow), or an equivalent inventory equation, appears necessary.

5.The operating targets reported in Table 12 are not exactly the same as the reference PSO or deterministic results in Table 13. For example, LFFX is reported as 1.272 × 10⁶ m³ in Table 12, whereas the mean PSO target is approximately 1.280 × 10⁶ m³. Although the former lies within an equivalent-minimum interval, the manuscript should specify a unique target-selection rule and consistently use the selected values in the Abstract, figures, tables, and conclusions.

Reviewer #4: This manuscript examines a practical sequence from station-level day-ahead demand forecasting to hourly demand reconstruction and reduced-order linepack targets for the Chongqing regional gas network. I found the chronological model comparison and the transparent reporting of difficult stations useful. Please see my comments below.

1. I could not determine whether the operating targets in Table 12 were actually calculated from the out-of-sample forecasts reported in Section 3.3. The forecasting assessment uses a 30-day hold-out period, while the temporal reconstruction uses a separate archived 18-day case, and the connection between these records is not shown.

2. The intraday coefficients are not defined consistently. Equations 8 and 9 describe them as shares whose total equals one, but the values in Tables 4 and 5 are centered around one.

3. The construction of daily station demand also needs clarification. Section 2.2.2 says that hourly instantaneous flows measured in cubic metres per day were summed to obtain daily load, but a flow rate normally must be integrated over the sampling interval.

4. If inlet supply and withdrawals already determine the linepack trajectory, selecting a constant target that minimizes absolute deviations is essentially a one-dimensional median problem.

5. I think the manuscript should distinguish more carefully between compliance with prescribed envelopes and hydraulic feasibility.

6. The feature-selection results are not sufficiently reproducible from the manuscript. Fig 10 does not identify the station, forecast origin, or training interval, and it contains pressure and humidity variables that are absent from Table 2. Please reconcile the predictor lists and show how the final eight inputs were obtained across stations and forecast origins.

7. In terms of the topic of forecast-to-dispatch, I wouldl ike to recommend a relevant paper, Feature-driven Economic Improvement for Network-constrained Unit Commitment: A Closed-loop Predict-and-optimize Framework, which may help authors enhance your literature section.

**********

what does this mean?). If published, this will include your full peer review and any attached files.

If you choose “no”, your identity will remain anonymous but your review may still be made public.

**Do you want your identity to be public for this peer review?** For information about this choice, including consent withdrawal, please see our Privacy Policy

Reviewer #3: No

Reviewer #4: No

**********

[NOTE: If reviewer comments were submitted as an attachment file, they will be attached to this email and accessible via the submission site. Please log into your account, locate the manuscript record, and check for the action link "View Attachments". If this link does not appear, there are no attachment files.]

To ensure your figures meet our technical requirements, please review our figure guidelines: https://journals.plos.org/plosone/s/figures

You may also use PLOS’s free figure tool, NAAS, to help you prepare publication quality figures: https://journals.plos.org/plosone/s/figures#loc-tools-for-figure-preparation.

NAAS will assess whether your figures meet our technical requirements by comparing each figure against our figure specifications.

# Author comment: Author response to Decision Letter 2

**DOI:** 10.1371/journal.pone.0349981.r004

14 Sep 2026

Response to the Academic Editor and Reviewers

Manuscript ID: PONE-D-26-22124R1

A Forecast-to-Dispatch Workflow for Day-Ahead Operation of a Regional Natural Gas Transmission Network Based on Station-Level Load Prediction and Linepack Target Setting

We appreciate the opportunity to revise the manuscript. This response covers the Academic Editor and Journal Requirements, Reviewer 3 Comments 1-5, and Reviewer 4 Comments 1-7. The revisions clarify the operational evidence chain, units, input features, inventory state equation, and target-reporting convention. Remaining gaps in the archived records are stated explicitly. No forecasting models, full ablation experiments, or repeated-seed PSO studies were rerun in this final revision.

Responses to the Academic Editor and Journal Requirements

Editor

Comment

Please revise

Response

We have revised the manuscript to distinguish the common forecasting benchmark, the independent archived hourly-reconstruction evidence, and the retrospective engineering cases. The revision makes the hourly-rate units, fixed forecasting inputs, intertemporal inventory equation, and historical target-reporting rule explicit. We also added the recommended predict-and-optimize reference and consolidated the language and supporting-file descriptions.

The West-pipeline example is a retrospective reconstruction based on author-confirmed historical inputs and retained engineering records. Its correspondence to the predictions in the common 30-day benchmark remains unverified. The manuscript also reports the independently verified NGXDD inventory target, the separate limitation on replaying its archived pressure conversion, and the unavailable preprocessing records. The unverifiable archived cumulative-error column has been removed from Table 10.

We ask the Editor to consider the narrowed evidential scope and the explicitly identified archival gaps when assessing this revision.

Changes in the manuscript

Sections 2.2-2.6, 3.2-3.6, Engineering implications and limitations, Conclusions, Tables 1, 2, 10, 12-14, and S6 Table.

Submission files

Comment

A letter that responds to each point raised by the academic editor and reviewer(s). You should upload this letter as a separate file labeled 'Response to Reviewers'.

A marked-up copy of your manuscript that highlights changes made to the original version. You should upload this as a separate file labeled 'Revised Manuscript with Track Changes'.

An unmarked version of your revised paper without tracked changes. You should upload this as a separate file labeled 'Manuscript'.

If you would like to make changes to your financial disclosure, please include your updated statement in your cover letter. Guidelines for resubmitting your figure files are available below the reviewer comments at the end of this letter.

Response

The resubmission includes a clean revised manuscript, a cumulative marked-up manuscript showing the revisions from the version reviewed, this point-by-point response, and the updated Supporting Information files. The funding and competing-interest statements remain unchanged.

Changes in the manuscript

Clean revised manuscript; cumulative marked-up manuscript; complete point-by-point response; updated Supporting Information.

Journal Requirement 1

Comment

If the reviewer comments include a recommendation to cite specific previously published works, please review and evaluate these publications to determine whether they are relevant and should be cited. There is no requirement to cite these works unless the editor has indicated otherwise.

Response

We evaluated the recommended Chen et al. study for its relevance to the forecast-to-dispatch interface. It presents closed-loop, cost-oriented prediction for network-constrained unit commitment. We added it to the Introduction as reference [21] and distinguish its economic training objective from the sequential forecasting and inventory-deviation objective used here.

Changes in the manuscript

Introduction, paragraph beginning "Closed-loop predict-and-optimize research"; reference [21].

Revised text

Closed-loop predict-and-optimize research links forecasting to the downstream decision objective. Chen et al. trained a cost-oriented renewable-generation predictor using the objective and constraints of network-constrained unit commitment [21]. Their work distinguishes predictive accuracy from operating cost. The present gas-network workflow uses sequential forecasting and inventory preparation with a linepack-deviation objective.

[21] Chen X, Yang Y, Liu Y, Wu L. Feature-driven economic improvement for network-constrained unit commitment: A closed-loop predict-and-optimize framework. IEEE Transactions on Power Systems. 2022;37(4):3104-3118. doi:10.1109/TPWRS.2021.3128485.

Journal Requirement 2

Comment

Please review your reference list to ensure that it is complete and correct. If you have cited papers that have been retracted, please include the rationale for doing so in the manuscript text, or remove these references and replace them with relevant current references. Any changes to the reference list should be mentioned in the rebuttal letter that accompanies your revised manuscript. If you need to cite a retracted article, indicate the article’s retracted status in the References list and also include a citation and full reference for the retraction notice.

Response

We checked the correspondence between numbered in-text citations and bibliography entries, added Chen et al. as reference [21], and renumbered subsequent citations. Six uncited entries remaining from earlier versions were removed: ElMahdy et al. (2025), Carpenter (2024), Liu et al. (2020), Cafaro et al. (2022), Guzzo et al. (2024), and Vidas et al. (2022). The resulting list contains 30 cited entries.

We checked the 30 retained references against Crossref bibliographic metadata and verified the new Chen citation against the institutional publication record. The metadata check corrected reference [4] to the 2026 volume 6, issue 1 record and removed an extra word from the title of reference [11]. No retraction or withdrawal update was flagged in the returned Crossref records. This check is limited to the available metadata and does not constitute exhaustive publisher-by-publisher clearance.

Changes in the manuscript

Introduction and References; numbering throughout the manuscript.

Revised text

[21] Chen X, Yang Y, Liu Y, Wu L. Feature-driven economic improvement for network-constrained unit commitment: A closed-loop predict-and-optimize framework. IEEE Transactions on Power Systems. 2022;37(4):3104-3118. doi:10.1109/TPWRS.2021.3128485.

Responses to Reviewer 3

Comment 1

Comment

The forecasting evaluation uses a 30-day walk-forward test, whereas the temporal-disaggregation validation is based on a separate archived 18-day dataset. It remains unclear whether the reported linepack targets are generated from the same forecast origins evaluated in the forecasting section. At least one fully traceable example from station forecast → hourly reconstruction → segment demand → linepack target should be provided.

Response

We cannot establish from the retained records that the operating targets in Table 12 were generated from the exact forecast outputs evaluated in Section 3.3. The 30-day forecasting benchmark and 18-day hourly-reconstruction archive are separate evaluations. We have preserved that distinction and added a retrospective West-pipeline example for 22 April 2023 in Section 3.6, Table 14, and S6 Table.

The authors confirmed the case date, the end-of-day forecast origin of 21 April 2023, the five historical station forecast inputs, and the participation rule. The weighted sum is 4,817,675.298713333 m³/d. The archived engineering implementation rounded this to 481.77 × 10⁴ m³/d before hourly disaggregation. Multiplication by the hourly factors used in the reconstruction reproduces all 24 Table 4 withdrawal rates at the displayed precision. With the archived supply and safe-envelope calibration, the inventory recurrence gives 25 boundary states. The selected LFFX preparation value of 1,271,536.975841198 m³ lies in the continuous optimal interval and attains the deterministic minimum.

The example provides a retrospective numerical chain conditional on author-confirmed historical inputs; their correspondence to the current common benchmark remains unverified. Station K contributes to this historical engineering input but remains excluded from the shared performance comparison.

Changes in the manuscript

Sections 2.1, 2.4.3, 3.6; Table 14; S6 Table; Engineering implications and limitations.

Revised text

The West-pipeline case for 22 April 2023 was reconstructed from historical station-demand inputs, participation coefficients, and engineering calculation records. The forecast origin was the end of 21 April 2023. Table 14 shows the calculation sequence for the 22 April 2023 West-pipeline case. S6 Table shows the input sources and full-precision numerical trace. Whether these historical inputs match predictions from the current common 30-day benchmark remains unverified.

The weighted station forecasts gave an unrounded daily aggregate of 4,817,675.298713333 m³/d, or 481.7675298713333 × 10⁴ m³/d. The engineering implementation rounded this aggregate to two decimal places in units of 10⁴ m³/d, giving a baseline of 481.77 × 10⁴ m³/d. Multiplication by the 24 full-precision hourly unevenness factors reproduced the withdrawal rates in Table 4 at the reported precision.

The median-set calculation yielded a continuous optimal interval of approximately 1,269,828.333–1,289,124.243 m³. The PSO-selected preparation value, 1,271,536.975841198 m³, lay within this feasible interval and attained the minimum mean absolute deviation of 64,168.628 m³.

Comment 2

Comment

The manuscript should explain more explicitly how source-point missingness is propagated through hourly imputation, station aggregation, and daily-load construction, and report station-level completeness before and after imputation.

Response

Thank you for this comment. We revised Sections 2.2.1–2.2.3 and Table 1 to clarify the missing-data pathway from the received source-point archive to the station-level daily modeling series.

The revised manuscript distinguishes the data owner’s routine operational quality control from the research-side preprocessing performed after data receipt. Remaining source-point gaps were handled at hourly resolution, hourly flow rates were converted to daily mean rates, source points assigned to the same station were aggregated spatially, and remaining complete-day gaps were handled at daily resolution.

Table 1 now reports the available received-archive source-point missingness summaries by station and a separate audit of the released station-level daily data. The released daily audit contained no residual missing cells after research-side processing.

Exact station-level completeness counts before and after the original hourly-to-daily preprocessing could not be reconstructed because the record-level preprocessing counters were not retained. We have stated this limitation explicitly and report the retained missingness summaries and released-daily audit instead of inferring unsupported counts.

Revision made

Section 2.2.1: clarified data-owner quality control before data delivery.

Section 2.2.1 and the text adjacent to Table 1: clarified received-archive missingness, aggregation, denominators, and the separate released-daily-data audit.

Section 2.2.3: clarified hourly treatment, daily averaging, station aggregation, and daily gap treatment. The unavailable original before/after counters are stated once in Section 2.2.1.

Revised text

Table 1 shows received-archive source-point missingness and the separate audit of the released station-level daily series. The missingness summaries cover records received after data-owner quality control and before research-side imputation. Station missing rates are unweighted means of the constituent source-point rates. Complete-day-gap shares are missing-rate-weighted means of the source-point missing-type proportions, with total missingness as the denominator, not all calendar days.

In the daily-series audit, outlier percentages use the retained daily-row count N as the denominator. The final column reports N, daily-load values imputed after outlier screening, and residual missing cells across eight source/derived columns. These columns are load, three temperatures, month, weekday, weekend, and holiday. No residual missing cells remained. Record-level counters for the original hourly-to-daily preprocessing, including station-level completeness before and after processing, were not retained.

Comment 3

Comment

The authors should either implement station-specific model selection in the actual forecast-to-dispatch workflow or clearly justify why the hybrid model is retained for downstream target setting.

Response

We follow the second option in the comment: the manuscript now explains why the historical forecasting inputs are retained. CNN-BiLSTM-Attention is the focal architecture for the architectural and ablation analysis and formed the original engineering forecasting workflow. Retaining the historical station forecasts preserves the provenance of the retrospective West-pipeline calculation; substituting models selected after the revised benchmark would define a different case.

The benchmark does not support imposing the hybrid model at every station. We recommend station-specific model selection using chronological validation for prospective deployment. This recommendation has not been implemented retrospectively, and the 30-day hold-out outcomes were not used to select new deployed models or regenerate the historical inputs. No forecasting model was retrained during this final revision.

Changes in the manuscript

Sections 2.3, 2.6.2, 3.3.2, and 3.5; Conclusions.

Revised text

CNN-BiLSTM-Attention is the focal hybrid architecture because it formed the original engineering forecasting workflow and supports the architectural ablation analysis. The historical hybrid forecasts were retained to reconstruct the original engineering case.

For prospective deployment, candidate models should be compared by chronological validation within each station. The selected model would then generate that station's day-ahead forecast.

The best-performing model differed by station. SARIMAX and XGBoost were competitive at several regular and volatile stations, while recurrent and hybrid models performed well at other sites. These results support station-specific model selection for prospective deployment, with CNN-BiLSTM-Attention considered alongside the other candidate models.

Comment 4

Comment

A more fundamental issue exists in Eq. (22). The equation is written as an instantaneous flow/mass-balance constraint but contains no intertemporal linepack accumulation term. Therefore, it is unclear how Eq. (22) can “generate the 24-h linepack trajectory” as stated in Section 2.5.3. A state-transition relation such as V(k+1) = V(k) + Δt × (inflow − outflow), or an equivalent inventory equation, appears necessary.

Response

We agree that the previous instantaneous balance did not define an inventory trajectory. In the revised manuscript, Eq. (28) gives the intertemporal state transition and its initial condition. The decision variable is the initial preparation inventory xₑ, with Vₑ,₀(xₑ) = xₑ. Each step adds Δt times the prescribed inlet-minus-withdrawal rate; both rates are in standard m³/d and Δt is 1/24 day.

The 24 hourly intervals generate 25 boundary states, including Vₑ,₂₄. The objective in Eq. (25) retains the 24 interval-start deviations from the hourly reference trajectory. The terminal state is checked for conservation and envelope compliance but is not inserted as an extra objective term. Eqs. (29) and (30) give the cumulative-state representation, and Section 2.5.3 gives the resulting median-set characterization. The NGXDD inventory calculation and all 25 boundary states have been independently verified under its retained segment-specific inputs.

Changes in the manuscript

Section 2.5.2, Eqs. (28)-(30), and Section 2.5.3.

Revised text

The objective averages the absolute deviations of the 24 interval-start states Vₑ,₀(xₑ),...,Vₑ,₂₃(xₑ) from their hourly references. The terminal state Vₑ,₂₄(xₑ) is included in the end-of-day conservation and envelope-feasibility checks, but not in the 24-term objective.

where Qₑ,ₖⁱⁿ and Qₑ,ₖᵒᵘᵗ are the prescribed inlet-supply and aggregate withdrawal rates, standard m³/d; and Δt = 1/24 day is the time step.

The horizon contains Nₕ = 24 hourly intervals and 25 boundary states. Vₑ,ₖ is the segment linepack at boundary k = 0,...,Nₕ, expressed as standard-condition gas volume, m³.

Eq. (23) relates this inventory to segment-average pressure:

Comment 5

Comment

The operating targets reported in Table 12 are not exactly the same as the reference PSO or deterministic results in Table 13. For example, LFFX is reported as 1.272 × 10⁶ m³ in Table 12, whereas the mean PSO target is approximately 1.280 × 10⁶ m³. Although the former lies within an equivalent-minimum interval, the manuscript should specify a unique target-selection rule and consistently use the selected values in the Abstract, figures, tables, and conclusions.

Response

The reporting rule now distinguishes the mathematical minimizing set, the single historical value reported for a case, and repeated-run solver statistics. The retained PSO selection is used as the operating target only when its inventory feasibility and optimal-set membership can be verified under the documented case inputs. Solver means and sampled-grid points remain diagnostics and do not replace that selection.

For LFFX, the archived value is 1,271,536.975841198 m³; the continuous optimal interval is approximately 1,269,828.333-1,289,124.243 m³ and the minimum mean absolute deviation is 64,168.628 m³. Its objective gap is zero. The two sampled-grid minima, 1.2712 and 1.2812 × 10⁶ m³, are distinguished from the complete continuous interval. For DLFX, the retained selection is 1,571,469.794304801 m³ within 1,559,382.189-1,573,735.267 m³, with a minimum mean absolute deviation of 71,567.257 m³ and zero gap. Both cases pass all-25-state inventory and associated pressure-envelope checks. Their displayed operating values are unchanged.

For NGXDD, the previously reported 480/530 discrepancy was resolved by restoring the correct segment-specific daily withdrawal baseline of 480 × 10⁴ m³/d. Both the inlet-supply and withdrawal baselines are therefore 480 × 10⁴ m³/d, and hourly withdrawals use the retained hourly unevenness factors. Independent deterministic verification places the archived selected linepack value of 4,701,756.095919851 m³ within the continuous minimizing interval of 4,695,278.411–4,707,161.027 m³. It attains the minimum mean absolute deviation of 64,746.628 m³, with zero objective gap, and the complete 25-state inventory trajectory satisfies the retained inventory constraints. Table 12 consequently retains 4.702 × 10⁶ m³ as the selected operating target. The reported preparation pressure of 4.86 MPa is retained from the archived engineering record because the matching pressure-conversion worksheet was not preserved and was unavailable for independent replay. This pressure-specific limitation does not invalidate the independent inventory verification.

Table 13 reports solver-returned values and PSO and sampled-grid diagnostics based on retained objective-support data; its numerical values are unchanged and do not replace the historical selected operating targets in Table 12. The Abstract, Section 3.6, Table 12, and Conclusions consistently report independently verified linepack preparation values for all three segments. The text adjacent to Table 12 and the Fig 12 caption identify the NGXDD pressure as an archived reported value. S6 Table records the NGXDD 480 baseline, hourly withdrawal reconstruction, continuous minimizing interval, minimum mean absolute deviation, and V₀–V₂₄ inventory checks, with the unavailable pressure-conversion worksheet identified separately.

Changes in the manuscript

Section 2.5.3 reporting rule; Section 3.6; Fig 12 caption; Table 12 and its adjacent text; Table 13 and its adjacent text (numerical values unchanged); Section 4; Abstract and Conclusions; S6 Table.

Revised text

For each historical case, the archived PSO-selected preparation value is reported as the operating target after confirming inventory feasibility and optimal-set membership. When several minimizers exist, the archived selected member fixes the reported operating value.

Table 12 and Fig 12 show the selected preparation values for the three historical engineering cases. The LFFX, NGXDD, and DLFX linepack targets were 1.272, 4.702, and 1.571 × 10⁶ m³, respectively, and passed case-specific deterministic inventory checks. These targets imply packing for LFFX and drafting for NGXDD and DLFX. The NGXDD pressure of 4.86 MPa is an archived reported value; its conversion could not be independently reproduced from the available records.

Table 13 shows the PSO results for the three computational budgets and the deterministic grid references. PSO results are reported as mean ± standard deviation over 30 runs. Table 12 shows the selected operating targets separately from these solver statistics. Under the 20-particle, 10-iteration setting, mean differences from the sampled-grid objective minima were 0.000%, 0.172%, and 0.008% for LFFX, NGXDD, and DLFX, respectively. The two optimal LFFX grid samples, 1.2712 and 1.2812 × 10⁶ m³, differ from the full continuous minimizing interval of approximately 1.269828333–1.289124243 × 10⁶ m³.

For NGXDD, inlet-supply and daily withdrawal baselines were both 480 × 10⁴ m³/d. The hourly withdrawal profile was reconstructed using the historical unevenness factors. The selected preparation value of 4,701,756.095919851 m³ lay within the continuous minimizing interval of 4,695,278.411–4,707,161.027 m³. It attained the minimum mean absolute deviation of 64,746.628 m³. All 25 inventory states satisfied the inventory constraints, supporting the reported preparation value of 4.702 × 10⁶ m³.

Responses to Reviewer 4

Comment 1

Comment

I could not determine whether the operating targets in Table 12 were actually calculated from the out-of-sample forecasts reported in Section 3.3. The forecasting assessment uses a 30-day hold-out period, while the temporal reconstruction uses a separate archived 18-day case, and the connection between these records is not shown.

Response

The available archive does not establish that Table 12 was calculated from the exact out-of-sample predictions reported in Section 3.3. The manuscript now states the distinct roles of the 30-day benchmark, the independent archived 18-day reconstruction evidence, and the historical engineering records. It does not present them as a single common-benchmark end-to-end validation.

Section 3.6, Table 14, and S6 Table reconstruct the West-pipeline case for 22 April 2023 using the authors' confirmed historical station inputs and participation rule. The five station forecasts aggregate to 4,817,675.298713333 m³/d, which the archived implementation rounded to 481.77 × 10⁴ m³/d. Multiplication by the high-precision hourly factors used in the reconstruction gives 24 LFFX withdrawal rates. The calculation then propagates 25 inventory boundary states and verifies the selected preparation value and pressure conversion. The historical Station K forecast contributes to this case only and is excluded from the shared model-performance statistics.

The example is a retrospective reconstruction with documented numerical conventions; its correspondence to the current benchmark forecasts remains unverified. The eastern cases remain separate records, and the NGXDD inventory target is independently verified while its archived pressure-conversion limitation is reported separately.

Changes in the manuscript

Sections 2.1, 2.4.3, 3.6; Tables 12 and 14; Fig 12 caption; S6 Table; limitations.

Revised text

The West-pipeline case for 22 April 2023 was reconstructed from historical station-demand inputs, participation coefficients, and engineering calculation records. The forecast origin was the end of 21 April 2023. Table 14 shows the calculation sequence for the 22 April 2023 West-pipeline case. S6 Table shows the input sources and full-precision numerical trace. Whether these historical inputs match predictions from the current common 30-day benchmark remains unverified.

The daily mean demand forecasts were 4,097,629.44125, 13,405.20833, 84,157.875, 205,933.9583, and 833,097.631666666 m³/d for Stations J, E, K, H, and I, respectively. Their West-pipeline participation coefficients were 1, 1, 1, 1, and 0.5. The station-level forecasts were first aggregated using this participation rule, after which the aggregated daily demand was temporally disaggregated into the 24-h LFFX withdrawal profile.

The weighted station forecasts gave an unrounded daily aggregate of 4,817,675.298713333 m³/d, or 481.7675298713333 × 10⁴ m³/d. The engineering implementation rounded this aggregate to two decimal places in units of 10⁴ m³/d, giving a baseline of 481.77 × 10⁴ m³/d. Multiplication by the 24 full-precision hourly unevenness factors reproduced the withdrawal rates in Table 4 at the reported precision.

Comment 2

Comment

The intraday coefficients are not defined consistently. Equations 8 and 9 describe them as shares whose total equals one, but the values in Tables 4 and 5 are centered around one.

Response

The revised text defines the coefficients as dimensionless hourly unevenness factors with mean 1 across the 24 hourly intervals, equivalent to a sum of 24. Each hourly flow rate is the daily mean demand rate multiplied by its factor. Hourly interval volume is obtained by multiplication by 1/24 day. Eqs. (11)-(14) and the text introducing Tables 4 and 5 use this definition consistently; the archived hourly values are unchanged.

The West-pipeline example records the additional historical numerical convention: the aggregated daily forecast was rounded to two decimals in units of 10⁴ m³/d before multiplication by the high-precision hourly factors used in the reconstruction. S6 Table gives those factors and reproduces all 24 Table 4 values.

With the observed daily-mean baseline and mean-one factors, Eq. (19) provides the daily-volume conservation diagnostic. The date-level inputs and calculation records underlying the archived cumulative-error values of 2.2%, 1.9%, and 2.5% were not retained, and these values cannot be reconciled with the documented conservation relation. We therefore removed that column and its three entries from Table 10 and removed the associated cumulative-error statements from the main results. The entries were not replaced with zero or relabeled as another metric. Hourly MAPE, peak-timing error, and peak-demand magnitude error remain unchanged. The April intermediate rounding is not offered as an explanation for these percent-level values.

Changes in the manuscript

Sections 2.4.1 and 2.4.3; Tables 4, 5, and 10; Section 3.6; S6 Table.

Revised text

where is the reconstructed station hourly flow rate, m³/d; is the predicted daily mean demand rate, m³/d; and is the dimensionless hourly unevenness factor for station s, hour k, and day type c. The volume assigned to interval k is:

where Δt = 1/24 day, and is the gas volume assigned to interval k, m³.

Because the 24 hourly unevenness factors have a mean value of 1:

Temporal disaggregation therefore preserves the predicted daily mean demand rate. Equivalently, the sum of the 24 hourly interval volumes equals the predicted daily gas volume.

With the observed daily-mean baseline in Eq. (17), the mean-one condition in Eq. (12), and Δt = 1/24 day, the reconstructed daily volume equals the observed daily volume apart from numerical rounding.

Forecast and reconstruction errors were evaluated separately, without quantifying their propagation into transient pressure and flow responses. The effects of the observed 1.0-1.5 h peak-timing errors on preparation linepack and average-pressure targets were not quantified. Broader multi-day and multi-season end-to-end validation requires synchronized forecast, withdrawal, supply, and linepack-state records. At data-impaired sites such as Station G, telemetry repair remains a priority for reliable forecasting.

Comment 3

Comment

The construction of daily station demand also needs clarification. Section 2.2.2 says that hourly instantaneous flows measured in cubic metres per day were summed to obtain daily load, but a flow rate normally must be integrated over the sampling interval.

Response

Section 2.2.2 now distinguishes time aggregation from spatial aggregation. The hourly SSLL records are standard-volume flow rates in m³/d. Their complete-day arithmetic mean is the daily mean rate; summing the 24 hourly rates multiplied by 1/24 day gives daily volume in m³. Daily source-point mean rates are then summed spatially across the source points assigned to each station.

The daily mean rate and one-day volume have equal numerical values for a complete one-day interval but different units and physical meanings. The temporal-disaggregation and linepack sections use the same rate basis and explicit time-step conversion. This corrects the description and unit chain; it does not alter the archived Table 4 schedule.

Changes in the manuscript

Section 2.2.2, Eqs. (1)-(3), Table 2, and Section 2.4.1.

Revised text

The source telemetry contained source identifiers, timestamps, and instantaneous flow-rate records. The operational SSLL records are hourly standard-volume flow rates, m³/d. For a complete day with 24 equally spaced observations, the daily mean flow rate at source point p was calculated as:

where q is the hourly flow rate at source point p on day d at hour h, m³/d.

The corresponding daily gas volume is:

where Δt = 1/24 day. The daily mean rate is expressed in m³/d and the daily volume in m³.

Source-point daily mean flow rates assigned to the same station were then summed to obtain its daily mean demand rate:

where Ps is the set of source points assigned to station s.

Comment 4

Comment

If inlet supply and withdrawals already determine the linepack trajectory, selecting a constant target that minimizes absolute deviations is essentially a one-dimensional median problem.

Response

We agree. The initial preparation inventory is the scalar decision variable; the prescribed supply and withdrawal rates determine cumulative increments Cₑ,ₖ. A candidate therefore generates Vₑ,ₖ(xₑ) = xₑ + Cₑ,ₖ. The hourly reference Rₑ,ₖ is the midpoint of the prescribed safe envelope. Substitution into the archived mean absolute-deviation objective gives the median problem for the 24 transformed references Rₑ,ₖ - Cₑ,ₖ.

Section 2.5.3 states the continuous median interval and its intersection with the feasible candidate interval; if there is no intersection, the nearest feasible boundary minimizes the objective. PSO is retained as the archived numerical implementation, with repeated-run statistics used only as solver diagnostics. The selected historical value is distinguished from a unique mathematical minimizer. The terminal state is checked under the constraints but is excluded from the archived 24-term objective.

Changes in the manuscript

Sections 2.5.2 and 2.5.3; Eq. (25) and Eqs. (29)-(32); Section 3.6 and Tables 12 and 13.

Revised text

The objective averages the absolute deviations of the 24 interval-start states Vₑ,₀(xₑ),...,Vₑ,₂₃(xₑ) from their hourly references. The terminal state Vₑ,₂₄(xₑ) is included in the end-of-day conservation and envelope-feasibility checks, but not in the 24-term objective.

With prescribed inlet-supply and withdrawal profiles, the one-dimensional L1 objective has a deterministic median-set solution.

Let rₑ,ₖ = Rₑ,ₖ - Cₑ,ₖ, with the 24 values ordered from rₑ,₍₁₎ to rₑ,₍₂₄₎. The unconstrained minimizing set is:

If the median set intersects the feasible candidate interval implied by Eqs. (26) and (27), that intersection is optimal. Otherwise, the nearest feasible boundary minimizes the objective. PSO was the historical numerical implementation and was compared with this deterministic reference.

For each historical case, the archived PSO-selected preparation value is reported as the operating target after confirming inventory feasibility and optimal-set membership. When several minimizers exist, the archived selected member fixes the reported operating value.

Comment 5

Comment

I think the manuscript should distinguish more carefully between compliance with prescribed envelopes and hydraulic feasibility.

Response

The manuscript now uses segment-level envelope compliance to describe the reduced-order checks. The model propagates a lumped inventory state and converts it to an associated average-pressure reference under the retained equivalent-pipe assumptions. It does not solve transient nodal pressure redistribution, flow reversal, valve actions, compressor dispatch, or the control trajectory required to establish the initial preparation inventory.

The reported quantities are inventory-preparation and average-pressure references for operator review. Section 4 describes the transient hydraulic verification required before operational implementation. Section 3.6 identifies the independently verified NGXDD linepack preparation value and the archived reported pressure of 4.86 MPa; the matching pressure-conversion worksheet was unavailable. The Fig 12 caption labels the NGXDD pressure as an archived reported value. Its appearance within a plotted band is not presented as proof of transient hydraulic feasibility.

Changes in the manuscript

Sections 2.5.2, 3.6, and 4; Conclusions; the text adjacent to Table 12; Fig 12 caption.

Revised text

The preparation value is restricted to the interior allowable band. Every candidate-generated state is checked against the segment-level linepack and associated average-pressure envelopes.

Inlet supply is prescribed, and segment withdrawals follow the temporal-disaggregation and station-to-node mapping procedures in Section 2.4. The engineering cases use their historical hourly withdrawal inputs, with network topology, valve and compressor status, and nominal flow direction fixed.

Fig 12. Observed and selected preparation points. (A) West pipeline-LFFX. (B) East pipeline-NGXDD. (C) East pipeline-DLFX. Colored bands show the prescribed linepack-pressure envelopes. NGXDD pressure: archived reported value.

The selected linepack is a segment-level preparation reference for the start of the next-day horizon. The reduced-order calculation checks inventory and average-pressure envelopes under fixed boundary conditions. It omits actuator sequences, valve switching, compressor dispatch, flow reversal, nodal pressure redistribution, and transient dynamics. Compressor fuel, procurement cost, and pressure-fluctuation penalties are outside the objective. Operational implementation requires current boundary data, operator review, and transient hydraulic verification. MPC was not benchmarked because the formulation lacks the closed-loop dynamic states and control actions needed for a matched comparison.

Comment 6

Comment

The feature-selection results are not sufficiently reproducible from the manuscript. Fig 10 does not identify the station, forecast origin, or training interval, and it contains pressure and humidity variables that are absent from Table 2. Please reconcile the predictor lists and show how the final eight inputs were obtained across stations and forecast origins.

Response

We removed the earlier MIC figure because its retained matrix did not identify the station, forecast origin, or fitting interval. The former Figs 11-13 are renumbered as Figs 10-12, with corresponding updates to captions and references.

The revised text describes the fixed eight-dimensional input schema used in the implementation. It contains denoised daily load, temperature PC1, sine/cosine encodings for day of week and month, and weekend and holiday indicators. At each origin, the temperature scaling, PCA, and other fitted transformations use the chronological model-fitting subset. Archived preprocessing records support one temperature component meeting the 95% explained-variance criterion. The schema stays fixed across stations and origins; MIC scores do not select columns at individual forecast origins.

Table 2 separates source variables from the final model representation. The sequence models receive 14 × 8 inputs; BPNN and XGBoost use the flattened 112-element window, and SARIMAX uses load history plus the seven exogenous dimensions. Pressure and humidity are not part of this final schema. Sections 2.3.1 and 3.2.2 and the revised workflow/architecture figures describe these actual inputs. The S1 Code documentation is synchronized with the fixed-feature implementation.

Changes in the manuscript

Table 2; Sections 2.2.2, 2.2.3, 2.3.1, and 3.2.2; Figs 3 and 4; deletion of former MIC Fig 10; renumbered Figs 10-12; S1 Code documentation.

Revised text

Candidate predictors comprised historical daily load, meteorological variables, and calendar indicators, with their engineering relevance considered during model development.

The forecasting pipeline used a fixed eight-dimensional feature vector at each daily time step. It comprised the wavelet-denoised station load, temperature PC1, sine and cosine encodings of day of week and month, and weekend and holiday indicators. With a 14-day lookback, CNN-BiLSTM-Attention, LSTM, GRU, and the sequence-based ablation models received a 14 × 8 multivariate sequence. BPNN and XGBoost used the flattened 112-element window. SARIMAX used normalized load history and the seven transformed exogenous dimensions obtained by excluding contemporaneous load. The feature schema remained fixed across stations and forecast origins. MIC-style dependence scores served as an exploratory development-stage diagnostic and did not determine feature columns at individual forecast origins.

Temperature compression reduced the three correlated measurements to a single principal component in the final input, alongside the denoised load and calendar features.

Comment 7

Comment

In terms of the topic of forecast-to-dispatch, I wouldl ike to recommend a relevant paper, Feature-driven Economic Improvement for Network-constrained Unit Commitment: A Closed-loop Predict-and-optimize Framework, which may help authors enhance your literature section.

Response

We added Chen et al. as reference [21] after evaluating its relevance to the forecasting-to-operation interface. Its closed-loop framework trains cost-oriented renewable-generation predictions using the objective and constraints of network-constrained unit commitment. The added Introduction paragraph distinguishes that closed-loop approach from the sequential forecast-guided inventory preparation and linepack-deviation objective used in this study.

The citation metadata were verified against the authors' institutional publication record: Chen X, Yang Y, Liu Y, Wu L; IEEE Transactions on Power Systems; 2022;37(4):3104-3118; doi:10.1109/TPWRS.2021.3128485.

Changes in the manuscript

Introduction, paragraph following the literature on forecasting and dispatch; reference [21].

Revised text

Closed-loop predict-and-optimize research links forecasting to the downstream decision objective. Chen et al. trained a cost-oriented renewable-generation predictor using the objective and constraints of network-constrained unit commitment [21]. Their work distinguishes predictive accuracy from operating cost. The present gas-network workflow uses sequential forecasting and inventory preparation with a linepack-deviation objective.

[21] Chen X, Yang Y, Liu Y, Wu L. Feature-driven economic improvement for network-constrained unit commitment: A closed-loop predict-and-optimize framework. IEEE Transactions on Power Systems. 2022;37(4):3104-3118. doi:10.1109/TPWRS.2021.3128485.

Attachment Submitted filename: Response_to_Reviewers_COMPLETE_Final_Synced.docx

# Peer review: Decision Letter 2

**DOI:** 10.1371/journal.pone.0349981.r005

**Authors:** Zhengmao Li

21 Sep 2026

A Forecast-to-Dispatch Workflow for Day-Ahead Operation of a Regional Natural Gas Transmission Network Based on Station-Level Load Prediction and Linepack Target Setting

PONE-D-26-22124R2

Dear Dr. Tian,

We’re pleased to inform you that your manuscript has been judged scientifically suitable for publication and will be formally accepted for publication once it meets all outstanding technical requirements.

Within one week, you’ll receive an e-mail detailing the required amendments. When these have been addressed, you’ll receive a formal acceptance letter and your manuscript will be scheduled for publication.

An invoice will be generated when your article is formally accepted. Please note, if your institution has a publishing partnership with PLOS and your article meets the relevant criteria, all or part of your publication costs will be covered. Please make sure your user information is up-to-date by logging into Editorial Manager at Editorial Manager® and clicking the ‘Update My Information' link at the top of the page. For questions related to billing, please contact billing support.

If your institution or institutions have a press office, please notify them about your upcoming paper to help maximize its impact. If they’ll be preparing press materials, please inform our press team as soon as possible -- no later than 48 hours after receiving the formal acceptance. Your manuscript will remain under strict press embargo until 2 pm Eastern Time on the date of publication. For more information, please contact onepress@plos.org.

Kind regards,

Zhengmao Li

Academic Editor

PLOS One

Additional Editor Comments (optional):

Reviewers' comments:

Reviewer's Responses to Questions

**Comments to the Author**

Reviewer #3: All comments have been addressed

Reviewer #4: All comments have been addressed

**********

2. Is the manuscript technically sound, and do the data support the conclusions?

Reviewer #3: Yes

Reviewer #4: Yes

**********

3. Has the statistical analysis been performed appropriately and rigorously?

Reviewer #3: Yes

Reviewer #4: Yes

**********

4. Have the authors made all data underlying the findings in their manuscript fully available?

The PLOS Data policy

Reviewer #3: Yes

Reviewer #4: Yes

**********

5. Is the manuscript presented in an intelligible fashion and written in standard English?

Reviewer #3: Yes

Reviewer #4: Yes

**********

Reviewer #3: (No Response)

Reviewer #4: Thank you for your efforts in addressing all my comments.

**********

what does this mean?). If published, this will include your full peer review and any attached files.

If you choose “no”, your identity will remain anonymous but your review may still be made public.

**Do you want your identity to be public for this peer review?** For information about this choice, including consent withdrawal, please see our Privacy Policy

Reviewer #3: No

Reviewer #4: No

**********

# Editor report: Acceptance letter

**DOI:** 10.1371/journal.pone.0349981.r006

**Authors:** Zhengmao Li

PONE-D-26-22124R2

PLOS One

Dear Dr. Tian,

I'm pleased to inform you that your manuscript has been deemed suitable for publication in PLOS One. Congratulations! Your manuscript is now being handed over to our production team.

At this stage, our production department will prepare your paper for publication. This includes ensuring the following:

* All references, tables, and figures are properly cited

* All relevant supporting information is included in the manuscript submission,

* There are no issues that prevent the paper from being properly typeset

You will receive further instructions from the production team, including instructions on how to review your proof when it is ready. Please keep in mind that we are working through a large volume of accepted articles, so please give us a few days to review your paper and let you know the next and final steps.

Lastly, if your institution or institutions have a press office, please let them know about your upcoming paper now to help maximize its impact. If they'll be preparing press materials, please inform our press team within the next 48 hours. Your manuscript will remain under strict press embargo until 2 pm Eastern Time on the date of publication. For more information, please contact onepress@plos.org.

You will receive an invoice from PLOS for your publication fee after your manuscript has reached the completed accept phase. If you receive an email requesting payment before acceptance or for any other service, this may be a phishing scheme. Learn how to identify phishing emails and protect your accounts at https://explore.plos.org/phishing.

If we can help with anything else, please email us at customercare@plos.org.

Thank you for submitting your work to PLOS One and supporting open access.

Kind regards,

PLOS One Editorial Office Staff

on behalf of

Dr Zhengmao Li

Academic Editor

PLOS One
